# Synthesis and biological evaluation of flavonoid-based IP6K2 inhibitors

**DOI:** 10.1080/14756366.2023.2193866

**Published:** 2023-04-04

**Authors:** Myunghwan Ahn, Seung Eun Park, Jiyeon Choi, Jiahn Choi, Doyoung Choi, Dongju An, Hayoung Jeon, Soowhan Oh, Kiho Lee, Jaehoon Kim, Jaebong Jang, Seyun Kim, Youngjoo Byun

**Affiliations:** a College of Pharmacy, Korea University, Sejong, Republic of Korea; b Department of Biological Sciences, Korea Advanced Institute of Science and Technology (KAIST), Daejeon, Republic of Korea; c KAIST Institute for the BioCentury and KAIST Stem Cell Center, KAIST, Daejeon, Republic of Korea; d Institute of Pharmaceutical Science and Translational Research, Korea University, Sejong, Republic of Korea

**Keywords:** Inositol polyphosphates, IP6K2, flavonoid, structure-activity relationship

## Abstract

Inositol polyphosphates (IPs) are a group of inositol metabolites that act as secondary messengers for external signalling cues. They play various physiological roles such as insulin release, telomere length maintenance, cell metabolism, and aging. Inositol hexakisphosphate kinase 2 (IP6K2) is a key enzyme that produces 5-diphosphoinositol 1,2,3,4,6-pentakisphosphate (5-IP7), which influences the early stages of glucose-induced exocytosis. Therefore, regulation of IP6Ks may serve as a promising strategy for treating diseases such as diabetes and obesity. In this study, we designed, synthesised, and evaluated flavonoid-based compounds as new inhibitors of IP6K2. Structure-activity relationship studies identified compound **20s** as the most potent IP6K2 inhibitor with an IC_50_ value of 0.55 μM, making it 5-fold more potent than quercetin, the reported flavonoid-based IP6K2 inhibitor. Compound **20s** showed higher inhibitory potency against IP6K2 than IP6K1 and IP6K3. Compound **20s** can be utilised as a hit compound for further structural modifications of IP6K2 inhibitors.

## Introduction

Myo-inositol (inositol), a carbocyclic sugar with one axial and five equatorial hydroxyl groups, is an essential nutrient for human health.^1^ The discovery of phospholipase C (PLC) has highlighted the biological significance of inositol polyphosphates (IPs), which are bioactive signalling secondary messengers.^2^^,^^3^ For example, inositol 1,4,5-trisphosphate (IP3) mediates cytosolic calcium release by opening an IP3-gated calcium channel.^2^ IP3 is further metabolised into higher phosphorylated IPs, such as IP4, IP5, and IP6 by a group of IP kinases, such as IP3-kinases, IP5-2 kinase, and inositol polyphosphate multikinase (IPMK).^3–7^

Among the many IP species commonly found in eukaryotic cells from yeast to humans, pyrophosphorylated-IPs (PP-IPs) have drawn attention because they have highly energetic phosphoanhydride bonds (pyrophosphates) at specific positions.^3^^,^^7^ PP-IP biosynthesis is catalysed by two groups of IP kinases, IP6 kinases (IP6Ks) and PPIP5 kinases (PPIP5Ks), which phosphorylate IP6 and IP7.^4^^,^^5^ In mammals, IP6Ks (IP6K1/2/3) phosphorylate IP6 at the 5-position to form 5-PP-IP5 (5-IP7),^8^^,^^9^ whereas PPIP5Ks (PPIP5K1/2) phosphorylate IP6 at the 1-position to produce 1-PP-IP5 (designated as 1-IP7).^10^^,^^11^ Compared with 5-IP7, 1-IP7 appears to be a better substrate for PP-IP phosphatases such as DIPP, which establishes higher levels of 5-IP7 than 1-IP7 in mammalian cells.^12^^,^^13^

As a signalling molecule, 5-IP7 modulates specific target proteins to control signalling events *via* different molecular interactions.^3^^,^^7^ For example, 5-IP7 allosterically interacts with the Akt PH domain, thereby inhibiting its recruitment to phosphatidylinositol 3,4,5-trisphosphates (PIP3) in the plasma membrane and its subsequent activation.^14^ Other 5-IP7 binding proteins include synaptotagmin, phosphatidylinositol 3-kinase (PI3K) p85α, and casein kinase.^15–17^ 5-IP7 is also known to non-enzymatically transfer its β-phosphates to serine residues that have been primed by CK2-mediated phosphorylation.^18^^,^^19^ Accumulating evidence has demonstrated that 5-IP7 and IP6 kinases regulate various biological events including growth, apoptosis, male fertility, metabolic homeostasis, blood clotting, immunity, vesicle trafficking, and longevity.^3^^,^^7^^,^^20^ Thus, increasing efforts are being undertaken for developing therapeutic options for managing pathological conditions such as obesity, type II diabetes, and cancer by pharmacologically targeting IP6Ks.^21–26^

**Figure 1.**
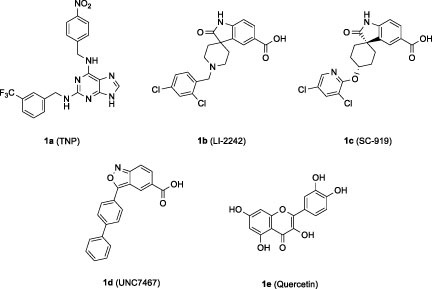
Examples of IP6K inhibitors.

Since IP6K is considered as a potential target for obesity and metabolic diseases, several synthetic and natural IP6K inhibitors have been described to date.^22–25^^,^^27^^,^^28^ One of the synthesised IP6K inhibitors is [*N*^2^−(m−(trifluoromethyl)benzyl) −*N*^6^−(*p*−nitrobenzyl)purine] (TNP) (**1a**), which acts as a competitive inhibitor by interacting with the ATP-binding site of IP6K (Figure 1).^22^ However, TNP has limitations for clinical use, including its inhibition of cytochrome P450 (CYP450), cellular Ca^2+^ fluxes, and several off-target kinases, such as CaMK1 and ERK.^24^^,^^27^^,^^29–31^ The second compound is an oxindole analog, LI-2242 (**1b**), which was reported as a potent IP6K inhibitor (IP6K1 IC_50_: 31 nM, IP6K2 IC_50_: 42 nM, IP6K3 IC_50_: 8.7 nM).^25^ Another oxindole analog, SC-919 (**1c**), was disclosed by Takeda Pharmaceuticals.^32^ The latest IP6K inhibitor is a benzisoxazole analog, UNC7467 (**1d**), which was recently reported as a potent IP6K inhibitor (IP6K1 IC_50_: 8.9 nM, IP6K2 IC_50_: 4.9 nM, IP6K3 IC_50_: 1323 nM) with selectivity of IP6K1 and IP6K2 over IP6K3.^23^

One of the natural IP6K2 inhibitors is quercetin (**1e**), a natural flavonoid.^24^ Gu et. al., reported 17 natural dietary flavonoids with 5-OH or 5,7-di-OH substitutions as IP6K2 inhibitors.^24^ Their inhibitory activity against IP6K2 varied according to the substitution pattern and number of hydroxyl groups in the A ring of the flavonoid scaffold. Generally, flavonoids with dihydroxyl groups at the A ring, such as quercetin and myricetin, exhibited stronger IP6K2 inhibition than monohydroxyl-substituted flavonoids. In addition, compounds with 3′,4′-di-OH substitution at the B ring inhibited IP6K2 more strongly than the corresponding compounds with 4′-OH substitution. Although the previous study identified natural flavonoids as hIP6K2 inhibitors with submicromolar IC_50_ values, a limited number of structure-activity relationship studies have been conducted on the 5- and 7-positions of the A ring. In this study, we designed, synthesised, and evaluated flavonoid-based analogs with a variation in the A ring to identify IP6K2 inhibitors that are more potent than natural flavonoids. Since synthetic IP6K inhibitors **1b**-**1d** contained -COOH functional group, we also included it as a substituent to modify the flavonoid-based IP6K inhibitors.

## Results and discussion

**Figure 2.**
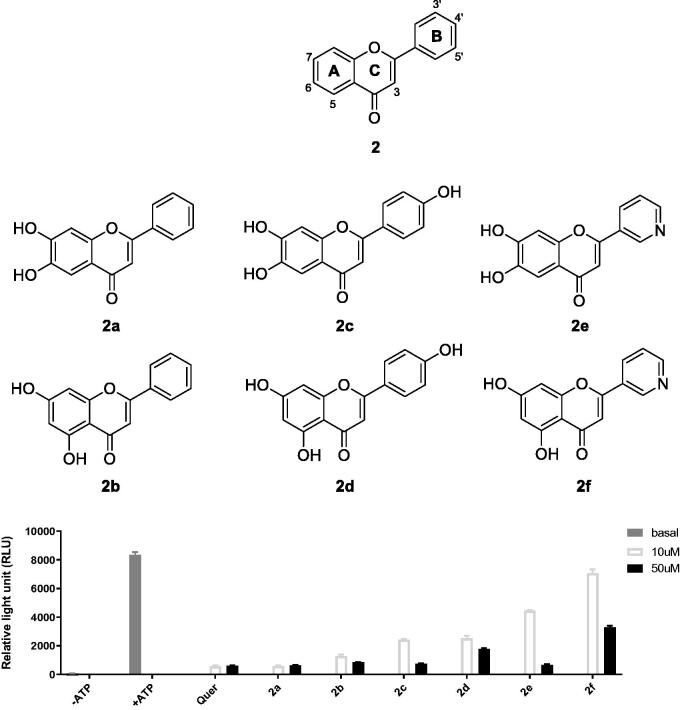
In-house flavonoid compounds evaluated using ADP-Glo assay.

Previously, we reported flavonoid analogs as potential inhibitors of thymic stromal lymphopoietin (TSLP), an alarmin cytokine involved in allergic immune responses.^33^ First, we screened the IP6K2-inhibitory activities of representative compounds (**2a**-**2f**, Figure 2) from an in-house compound library at concentrations of 10 and 50 µM using an *in vitro* ADP-Glo assay. We focussed on determining the effect of 5,7-di-OH substitution on IP6K2 inhibition compared to that of 6,7-di-OH substitution at the A ring, which is not common in natural flavonoids. Compounds (**2a**, **2c**, and **2e**) substituted with 6,7-di-OH were found to inhibit IP6K2 more strongly than the corresponding compounds **(2b**, **2d**, and **2f**) with 5,7-di-OH.

**Scheme 1.**
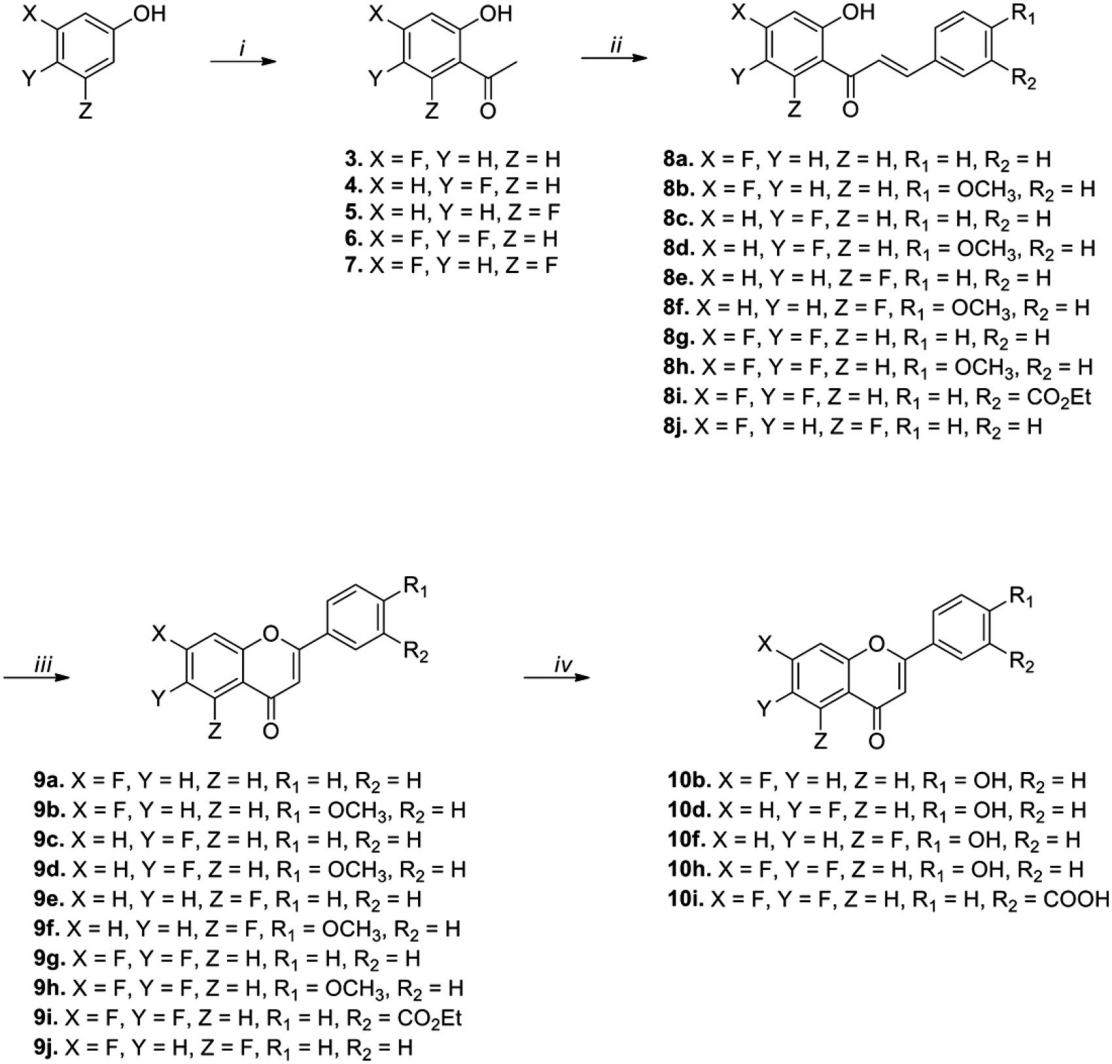
Synthesis of fluoro-substituted flavonoid analogs with a variation of the B ring. ***Reagents and conditions*:** (*i*) (*a*) Acetyl chloride, pyridine, CH_2_Cl_2_, rt, 30 min, (*b*) AlCl_3_, 150 °C, 10 min; (*ii*) appropriate aldehydes, Ba(OH)_2_, MeOH (or EtOH), 50 °C, 1–17 h; (*iii*) I_2_, DMSO, 110 °C, 6–24 h; (*iv*) BBr_3_, CH_2_Cl_2_, 50 °C, 14–18 h.

On the basis of preliminary studies, we designed and synthesised flavonoid analogs by replacing the -OH group of the A ring with the -F group to investigate the necessity of the hydroxyl group for IP6K2 inhibition. Scheme 1 describes the synthesis of fluoro-substituted flavonoid derivatives with various functional groups on the B ring. Commercial acetophenones **3**–**5** were used as starting materials for the synthesis of monofluoro-substituted compounds. In the case of difluoro-substituted compounds, starting materials **6** and **7** were prepared from 3,4-difluorophenol and 3,5-difluorophenol, respectively, by applying a reported synthetic procedure.^34^^,^^35^

The reactions of compounds **3**–**7** and appropriate aldehydes with barium hydroxide in methanol (or ethanol) at 50 °C for 1–17 h afforded chalcone compounds **8a**-**8k** (44-95% yield) *via* the Claisen-Schmidt condensation reaction. The intramolecular cyclisation of compounds **8a**-**8k** with iodine (I_2_) at 110 °C for 6–24 h generated compounds **9a**-**9k** (47-97% yield). Compounds substituted with a methoxy group or an ethyl ester were reacted with BBr_3_ to yield OH-substituted compounds (**10b, 10d, 10f,** and **10h**) and COOH-substituted compound (**10i**), respectively. According to the results of the ADP-Glo assay, compounds (**9a**-**10h**) substituted with the -F group in the A ring showed considerably weaker IP6K2-inhibitory activity than the corresponding compounds with the -OH group at 10 and 50 μM concentrations (See Supporting Information Figures 1 & 2). Therefore, we maintained the -OH substituent in the A ring for further structural modification of the flavonoid-based IP6K2 inhibitors.

**Scheme 2.**
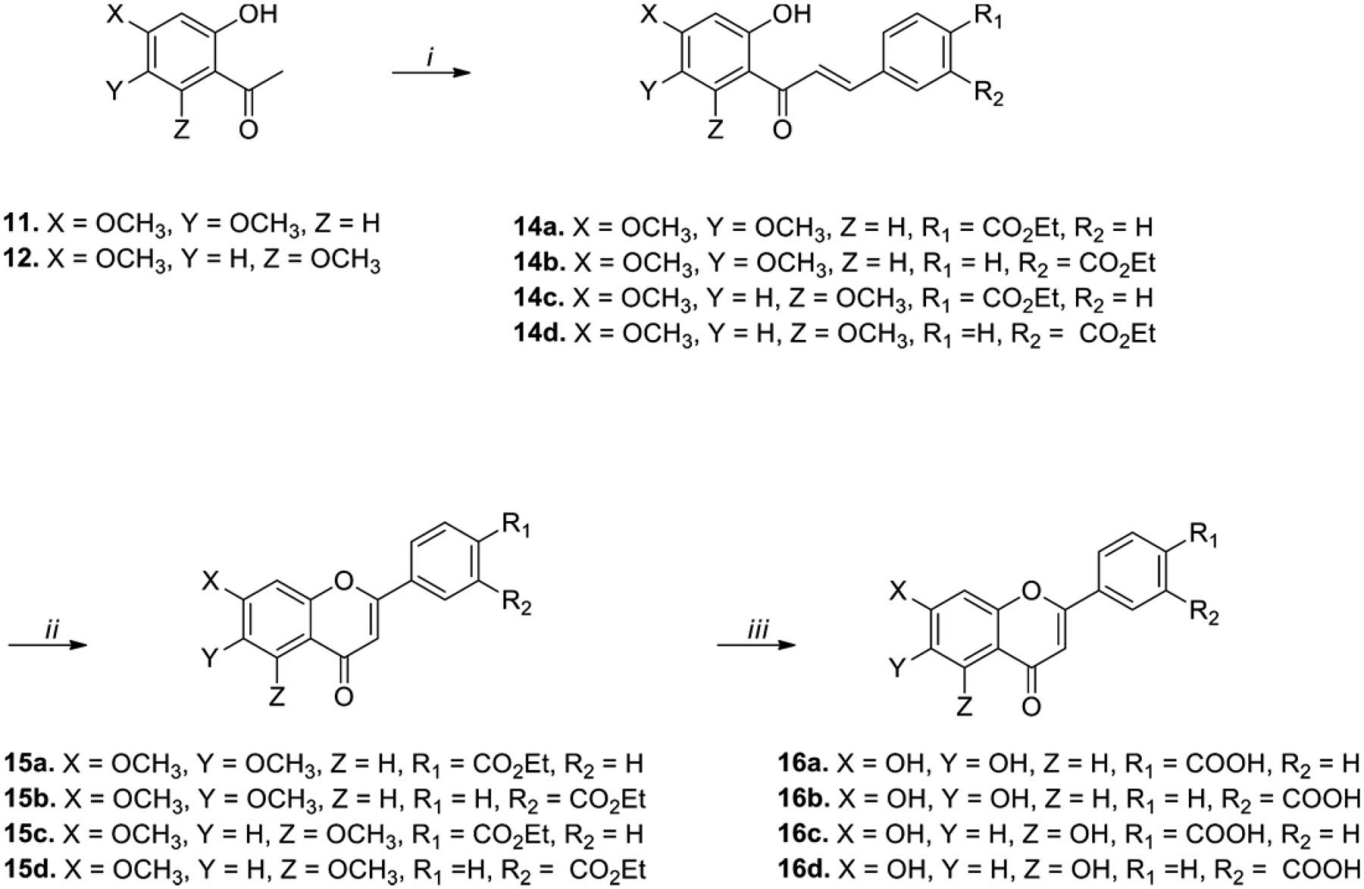
Synthesis of dihydroxy-substituted flavonoid analogs with a variation of the B ring. ***Reagents and conditions:*** (*i*) Appropriate aldehydes, Ba(OH)_2_, MeOH (or EtOH), 50 °C, 11–20 h; (*ii*) I_2_, DMSO, 110 °C, 11–17 h; (*iii*) BBr_3_, CH_2_Cl_2_, 50 °C, 5–18 h.

Next, we focussed on the structural modification of the B ring while maintaining the dihydroxyl substituents of the A ring at either the 5,7- or 6,7-positions. We introduced a polar carboxylic acid substituent at the *meta*- and *para*-positions of the B ring to generate potential ionic or hydrogen-bonding interactions. Scheme 2 describes the synthesis of flavonoid derivatives (**16a**-**16d**) with a carboxylic acid functional group in the B ring. The synthetic strategy for **16a**-**16d**, which was similar to that for the F-substituted compounds (Scheme 1), included Claisen-Schmidt condensation and intramolecular cyclisation starting from methoxy-substituted acetophenones. The global dealkylation step was achieved using boron tribromide in dichloromethane at 50 °C, affording the final compounds **16a-16d** (50-87% yield).

The ADP-Glo kinase assay was conducted using the synthesised compounds **16a**-**16d** (Table 1). Carboxylic-acid substitution at the *meta* position of the B-ring (**16b** and **16d**) led to superior IP6K2 inhibition compared to *para* substitution (**16a** and **16c**). Consistent with the results of the primary screening with **2a**-**2f**, compounds **16a**-**16b** with 6,7-dihydroxyl groups exhibited a stronger inhibitory effect on IP6K2 than the corresponding compounds **16c**-**16d** with 5,7-dihydroxyl groups. Compounds **16a** and **16b** were as potent as quercetin, which possesses an -OH group at the 3-position of the C-ring.

**Table 1.** IC_50_ values of the synthesised compounds **16a**-**16d** against IP6K2.

Comp.	IP6K2 IC_50_ (μM)	Comp.	IP6K2 IC_50_ (μM)
**16a**	9.07	**16c**	41.9
**16b**	1.67	**16d**	2.89

**Scheme 3.**
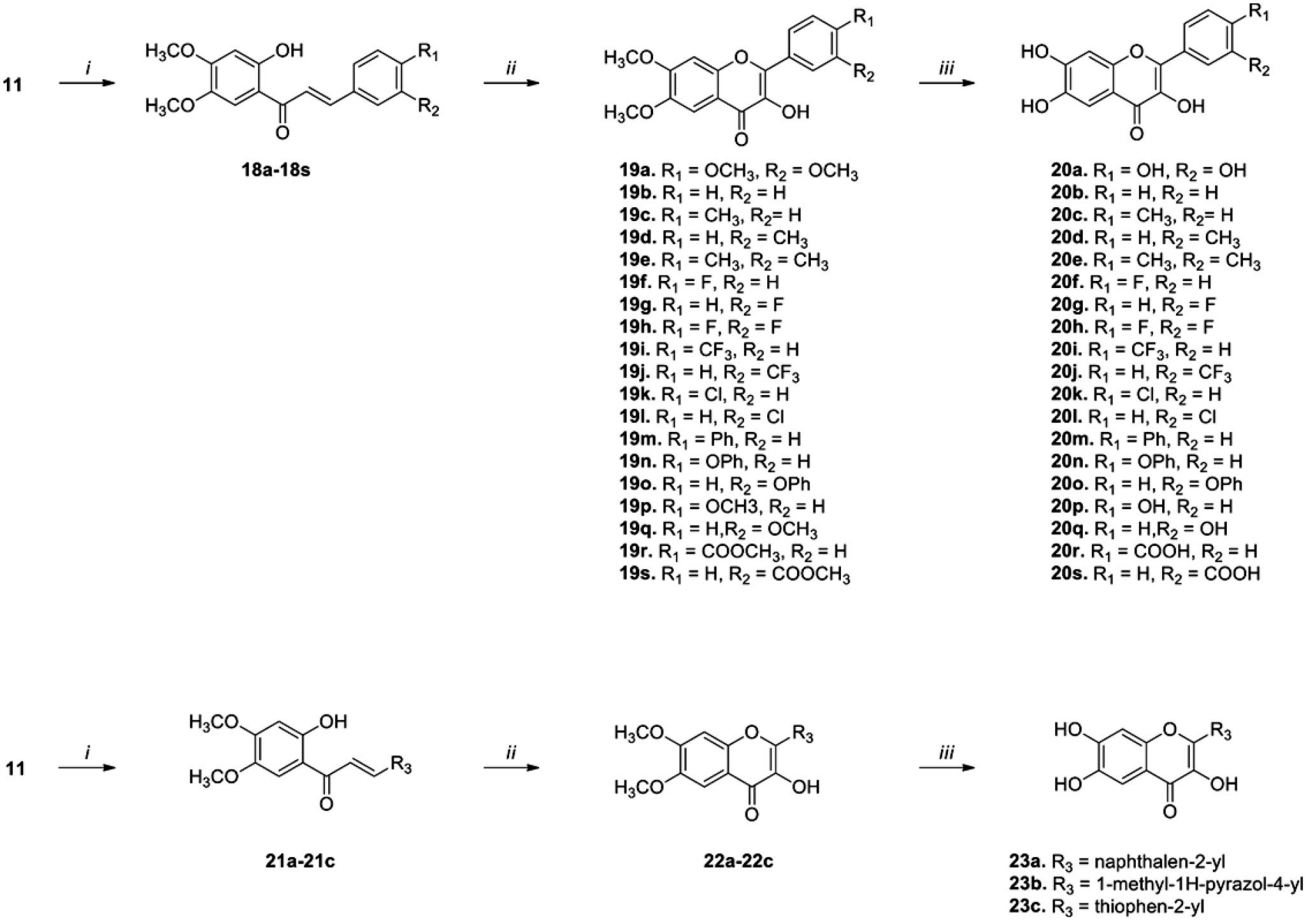
Synthesis of flavonoid analogs with a 3-OH group on the C ring. *Reagents and conditions*: (*i*) Appropriate aldehydes, NaOMe, THF, rt, 16–20 h; (*ii*) a. H_2_O_2_, NaOH (or NaOMe), EtOH (or MeOH), 40 °C, b. HCl, 16–20 h; (*iii*) BBr_3_, CH_2_Cl_2_, 50 °C, 6-16 h.

To increase IP6K2-inhibitory activity, we introduced a hydroxyl group at the 3-position of the C-ring. Scheme 3 describes the synthesis of compounds with an -OH group at the 3-position of the C ring. The reaction of **11** with the appropriate benzaldehydes under basic conditions (NaOMe in MeOH/THF) provided high yields of chalcone compounds (**18a-18s** and **21a-21c)**. The cyclized compounds (**19a-19q** and **22a-22c)** were obtained *via* the Algar-Flynn-Oyamada (AFO) reaction of chalcone compounds with hydrogen peroxide (35% aq.) and sodium hydroxide (3 M aq.) in ethanol (or methanol) at 40 °C for 16-20 h. In the case of methylester compounds **19r** and **19s**, sodium methoxide was applied in the AFO reaction instead of sodium hydroxide. Reaction of the cyclized compounds with boron tribromide in dichloromethane at 50 °C for 6–16 h afforded compounds (**20a-20s** and **23a-23c**) with substitution at the 3-OH of the C ring. The chemical structures and purities of the final compounds were confirmed using ^1^H NMR, LC/MS, and HPLC.

For accurate quantification of the IP6K2-inhibitory activity, the IC_50_ values of the synthesised compounds **20a-20s** and **23a-23c** were determined by performing an ADP-Glo kinase assay in a dose-dependent manner (Table 2). We confirmed the positional effect of OH groups in the A ring on IP6K2 inhibition by comparing compound **20a** with quercetin. The IC_50_ value of compound **20a** was 1.77 µM, while that of quercetin was 3.31 µM under the same experimental condition, consistently indicating that 6,7-substitution is preferred to 5,7-substitution in this series. The IC_50_ value of quercetin was reported as 0.70 µM.^24^ However, under our experimental conditions, it was 3.31 µM. The difference could be due to the different experimental conditions including the IP6K2 enzyme concentration and reaction temperature.

**Table 2.** IC_50_ values of the synthesised compounds against IP6K2.

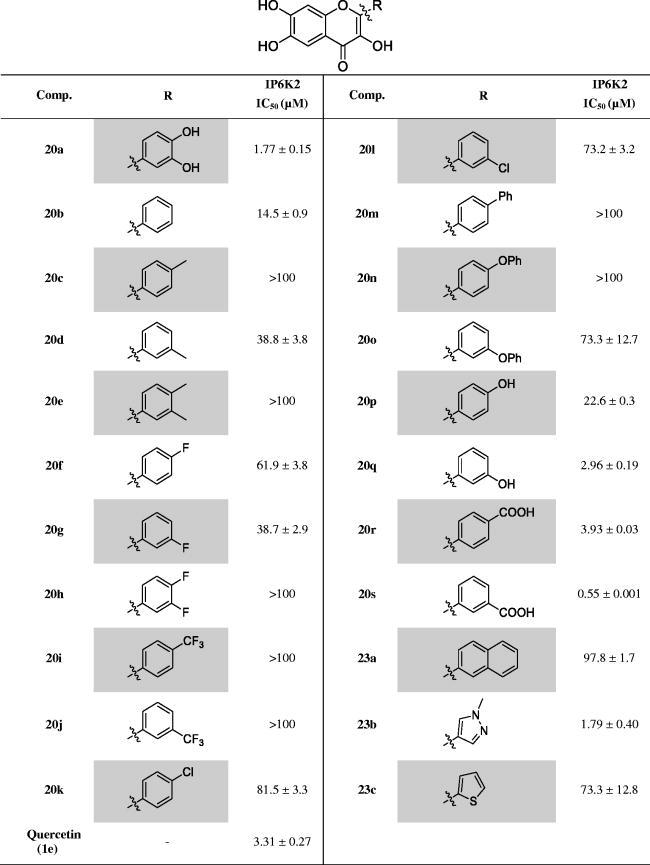

For the IC_50_ experiments, the compounds were tested in duplicates from 0.017 to 100 µM in eleven three-fold dilution steps. Values shown are the mean ± SD.

We examined the effects of substituents at the *meta*- or *para-*position of the B ring on IP6K2 inhibition. Compound **20b**, with no substituents on the B-ring, was synthesised as a control compound. Compounds **20c**-**20e** with a -CH_3_ group and compounds **20k**-**20l** with a -Cl group were less potent than compound **20b**. This result suggests that hydrophobic substituents on the B ring decrease IP6K2 inhibition. In addition, compounds **20f**-**20h** with the -F group and the CF_3_-substituted compounds **20i**-**20j** were less potent than **20b**. In particular, the disubstituted compounds (e.g. **20e** and **20h**) completely lost their inhibitory activity against IP6K2. This may be due to the increased volume of the hydrophobic substituents. This negative effect of increased volume due to lipophilic substituents was confirmed for compounds (**20m**-**20o**) with bulky and hydrophobic substituents, such as the -Ph and -OPh groups.

**Figure 3.**
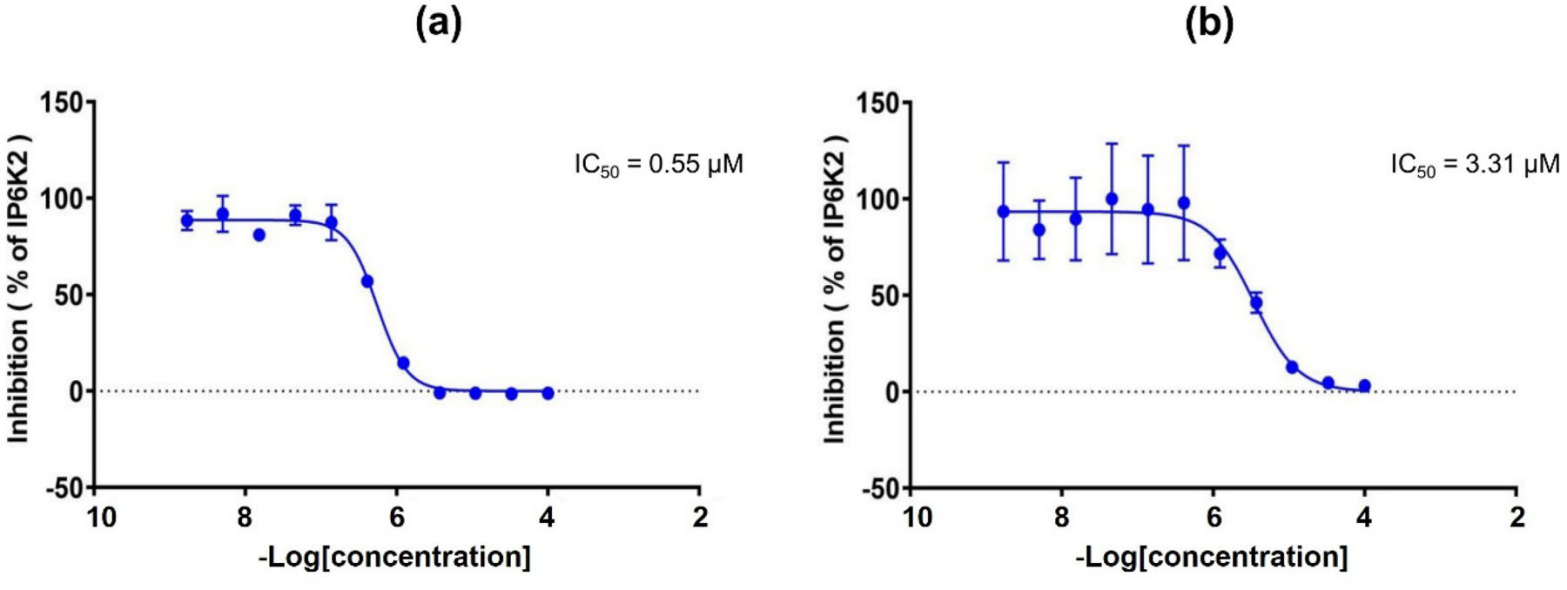
(a) Dose-response curve of compound **20s** against IP6K2. (b) Dose-response curve of quercetin against IP6K2.

In contrast, compound **20q**, with a hydrophilic -OH group at the *meta* position in the B ring (**20q**), strongly inhibited IP6K2 with an IC_50_ value of 2.96 μM and showed a 10-fold greater inhibition than compound **20p,** with the -OH group at the *para* position. Consistent with the result for analogs with a carboxylic acid substituent **16a-16d**, this result implies that the introduction of a hydrophilic substituent, particularly at the *m-*position of the B ring, contributes to increased IP6K2 inhibition. As expected, the substitution of the carboxylic acid group in the B ring resulted in an increase in IP6K2 inhibition. Compounds **20r** and **20s** showed strong inhibitory activity against IP6K2. In particular, the carboxylic compound **20s** was the most potent with an IC_50_ value of 0.55 µM, showing 5-fold more potent IP6K2 inhibition than quercetin (Table 2 and Figure 3). Compound **20s** has a carboxylic acid moiety at the *meta* position in the B ring, which is approximately 7-fold more potent than compound **20r** with the -COOH group at the *para* position. We additionally introduced heteroaromatic rings such as pyrazole (**23b**) and thiophene (**23c**). The pyrazole compound **23b** exhibited an IC_50_ value of 1.79 µM, rendering it 2-fold more potent than quercetin.

To figure out the selectivity for IP6K2 over IP6K1 and IP6K3, we decided to evaluate the inhibition rate of quercetin and the most potent compound **20s**. The IC_50_ value and the ratio of IP6K2 and other IP6Ks were shown in Table 3 (See Supporting Information Figure 3). Compound **20s** showed IC_50_ value of 2.87 µM against IP6K1 resulting 5.22-fold IC_50_ ratio of IP6K1/IP6K2 and IC_50_ value of 3.56 µM against IP6K3, resulting 6.47-fold IC_50_ ratio of IP6K3/IP6K2. When the final concentration of ATP and IP6 was adjusted to 10 μM, compound **20s** showed 4.65-fold and 2.32-fold IC_50_ ratio of IP6K1/IP6K2 and IP6K3/IP6K2, respectively (See Supporting Information Figure 4). This result represented that compound **20s** had selectivity against IP6K2 rather than IP6K1 or IP6K3 compared with quercetin. Overall, quercetin is a pan-IP6K inhibitor while compound **20s** is a more selective IP6K2 inhibitor. Next, we determined the membrane permeability of **20s** by an *in vitro* PAMPA permeability assay.^36^ Compound **20s** exhibited very low permeability with an apparent permeability coefficient of <1.8 nm/s while quercetin did with 5.4 nm/s (See Supporting Information Table 1). We further examined whether **20s** can inhibit IP7 synthesis in human colorectal cell line (HCT116) after treatment of the compound (10 μM) for 6 h since IP6K2 has been reported as a major enzyme for cellular IP7 synthesis among three isoforms of IP6Ks in HCT116.^37^ However, **20s** failed to reduce cellular IP7 levels (See Supporting Information Figure 5), suggesting its low cell permeability.

**Table 3.** Inhibition data of quercetin and compound **20s** against IP6K1/2/3.

Comp.	IP6K1 IC_50_ (μM)^a^	IP6K2 IC_50_ (μM)^b^	IP6K3 IC_50_ (μM)^c^	IP6K1 /IP6K2	IP6K3 /IP6K2
Quercetin	1.10	3.31	4.35	0.33	1.31
**20s**	2.87	0.55	3.56	5.22	6.47

^a^Final concentration of IP6K1 60 nM, substrate (IP6) 100 µM, ATP 1 mM; ^b^Final concentration of IP6K2 80 nM, substrate (IP6) 10 µM, ATP 10 µM; ^c^Final concentration of IP6K3 120 nM, substrate (IP6) 100 µM, ATP 1 mM.

**Figure 4.**
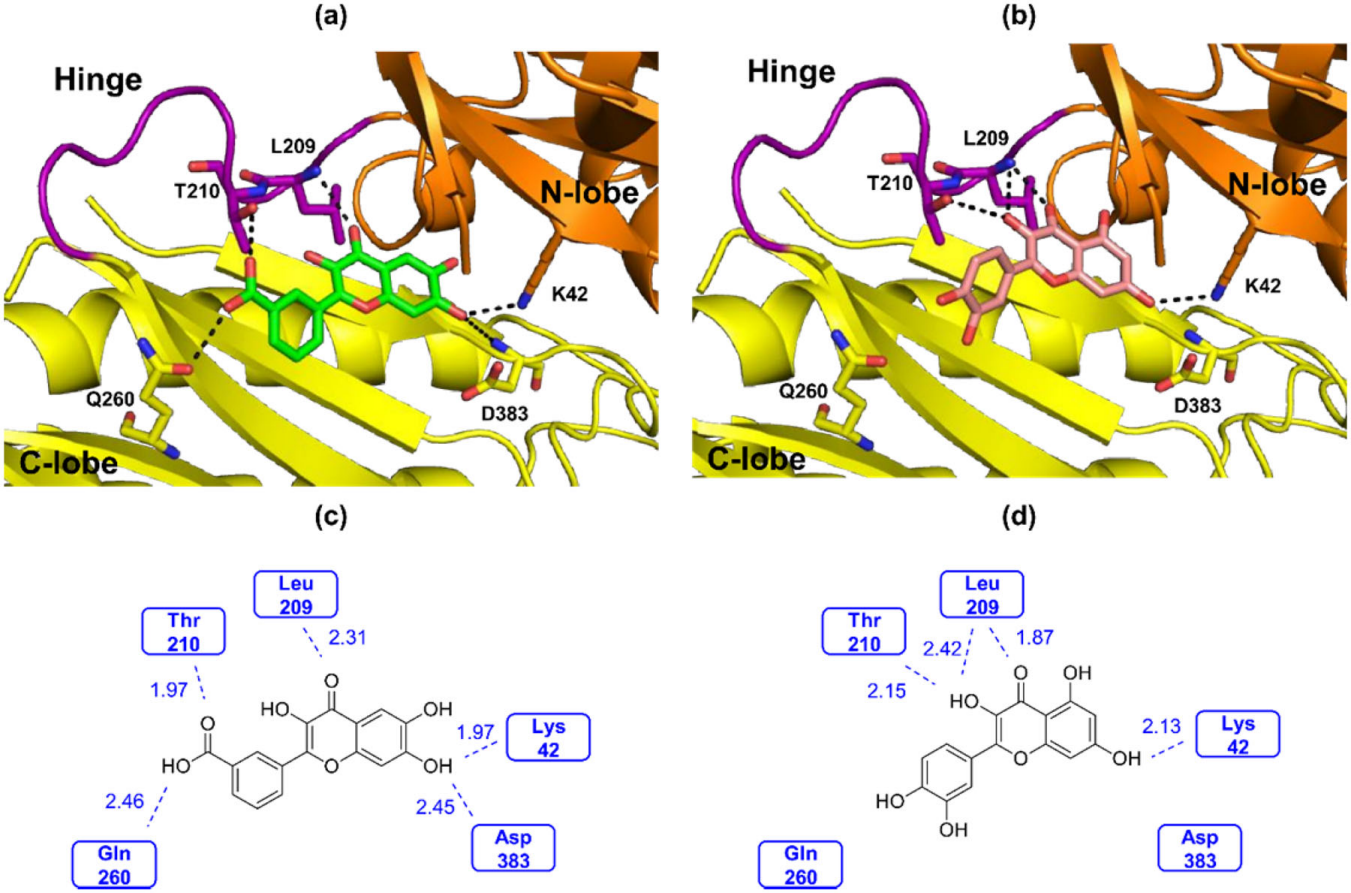
(a) The best-docked pose of compound **20s** with the 3D model of IP6K2. (b) The best-docked pose of quercetin with the 3D model of IP6K2. (c) Interaction between compound **20s** and IP6K2. (d) Interaction between quercetin and IP6K2.

*In silico* molecular docking studies of compound **20s** and quercetin were performed using the IP6K2 homology model. The homology model of human IP6K2 (UniProt: Q9UHH9) was downloaded from AlphaFold2 structure datable (https://alphafold.ebi.ac.uk). Quercetin has been reported as a ligand in the 3D crystal structure of IPMK (PDB ID: 6m89) and with that of DAPK1 (PDB ID: 5auw).^24^^,^^38^ According to the reported quercetin-bound protein crystal structures, quercetin can exist in two conformational isomers, designated 3,3′,4′,5,7-pentahydroxylflavone and 3,4′,5′,5,7-pentahydroxyflavone. Therefore, we performed the docking studies by using 3,3′,4′,5,7-pentahydroxylflavone and 3,4′,5′,5,7-pentahydroxyflavone for quercetin and 3′-(3,6,7-trihydroxy-4-oxo-4H-chromen-2-yl)benzoic acid and 5′-(3,6,7-trihydroxy-4-oxo-4H-chromen-2-yl)benzoic acid for compound **20s** as initial ligand structures. Although two conformers of **20s** were used as input files in the docking studies, only the docked pose exhibited with a conformation of 3′-(3,6,7-trihydroxy-4-oxo-4H-chromen-2-yl)benzoic acid (Figure 4(a,c)). On the contrary, two different conformers of quercetin were observed. The total score of the best-docked pose **20s** was 4.3908 while that of quercetin was 3.7869. Both compound **20s** and quercetin formed hydrogen bonds with Lys42 and with Leu209 (Figure 4). However, compound **20s** formed an additional hydrogen bond with Asp383 *via* the 7-OH group of the A ring compared to quercetin. In addition, the -COOH group in the B ring of compound **20s** formed a hydrogen bond with Thr210 and additional hydrogen bond with Gln260, whereas the 3-OH group of quercetin formed one hydrogen bond with Thr210. This result supports a stronger IP6K2 inhibition of compound **20s** than quercetin.

## Conclusion

In the present study, new flavonoid-based IP6K2 inhibitors were designed, synthesised, and evaluated *in vitro*. Systemic structure-activity relationship studies for IP6K2 inhibition revealed the substituent preference for each ring in the flavonoid backbone as follows: -OH groups at the 6- and 7-positions in the A ring, -COOH group at the *meta* position of the B ring, and -OH group at the 3-position of the C ring. Furthermore, hydrophilic substituents such as -OH and -COOH in the B ring imparted stronger IP6K2-inhibitory activity than hydrophobic ones, including -CH_3_, -Cl, CF_3_, -Ph, and -OPh. Additionally, compounds with a substituent at the *meta* position of the B ring were more effective than the corresponding compounds with a substituent at the *para* position. Among the compounds synthesised, compound **20s** was the most potent inhibitor with an IC_50_ value of 0.55 µM against IP6K2, which renders it 5-fold more potent than quercetin. In addition, compound **20s** showed higher inhibitory potency against IP6K2 than IP6K1 and IP6K3. The molecular docking study showed that compound **20s** formed additional hydrogen bonds with Gln260 and Asp383 compared to quercetin. Although compound **20s** showed low membrane permeability, its physicochemical properties can be improved by a prodrug approach. Overall, compound **20s** has the potential to be used as a hit compound for the structural optimisation of flavonoid-based IP6K2 inhibitors and as a tool compound for studying IP6K2 signalling pathways.

## Experimental section

### General

All chemicals and solvents used in the reaction were purchased from Sigma-Aldrich, TCI, and Acros and were used without further purification. Reaction progress was monitored by TLC on pre-coated silica gel plates with silica gel 60 F_254_ (Merck; Darmstadt, Germany) and visualised by UV254 light and/or KMnO_4_ staining for detection purposes. Column chromatography was performed on silica gel (Silica gel 60; 230–400 mesh ASTM, Merck, Darmstadt, Germany). A quantity of ∼1 mg was used for HSM experiment. Heating rate was 2 °C/minute and images were captured automatically every 1 min throughout the melting periods. Nuclear magnetic resonance (NMR) spectra were recorded at room temperature on a Bruker Ultrashield 600 MHz Plus (^1^H, 600 MHz; ^13^C, 150 MHz) spectrometer. All chemical shifts are reported in parts per million (ppm) from tetramethylsilane (*δ* = 0) and were measured relative to the solvent in which the sample was analysed (CDCl_3_: *δ* 7.26 for ^1^H NMR, *δ* 77.0 for ^13^C NMR; MeOH-*d*_4_: *δ* 3.31 for ^1^H NMR, *δ* 49.0 for ^13^C NMR; DMSO-*d*_6_: *δ* 2.50 for ^1^H NMR, *δ* 39.5 for ^13^C NMR). The ^1^H NMR shift values are reported as chemical shift (*δ*), the corresponding integral, multiplicity (s = singlet, br = broad, d = doublet, t = triplet, q = quartette, m = multiplet, dd = doublet of doublets, td = triplet of doublets, qd = quartette of doublets), coupling constant (*J* in Hz) and assignments. High-resolution mass spectra (HRMS) were recorded on an Agilent 6530 Accurate Mass Q-TOF LC/MS spectrometer. The purity of all final compounds was measured by analytical reverse-phase HPLC on an Agilent 1260 Infinity (Agilent) with a C18 column (Phenomenex, 150 mm × 4.6 mm, 3 μm, 110 Å). RP-HPLC was performed using various isocratic conditions: for method A, mobile phase was acetonitrile and water (55:45, v/v, 0.1% trifluoroacetic acid); for method B, mobile phase was acetonitrile and water (50:50, v/v, 0.1% trifluoroacetic acid); for method C, mobile phase was acetonitrile and water (45:55, v/v, 0.1% trifluoroacetic acid); for method D, mobile phase was acetonitrile and water (40:60, v/v, 0.1% trifluoroacetic acid); for method E, mobile phase was acetonitrile and water (35:65, v/v, 0.1% trifluoroacetic acid); for method F, mobile phase was acetonitrile and water (30:70, v/v, 0.1% trifluoroacetic acid); for method G, mobile phase was acetonitrile and water (25:75, v/v, 0.1% trifluoroacetic acid); for method H, mobile phase was acetonitrile and water (20:80, v/v, 0.1% trifluoroacetic acid); for method I, mobile phase was acetonitrile and water (15:85, v/v, 0.1% trifluoroacetic acid); for method J, mobile phase was acetonitrile and water (12:88, v/v, 0.1% trifluoroacetic acid). All compounds were eluted with a flow rate of 1 mL/min and monitored at a UV detector (220 nm or 254 nm). The purity of all tested compounds was >95%.

### Chemical synthesis

#### *General* Procedure A *for the synthesis of compounds 8a–8n and 14a–14d*

To a stirred solution of substituted acetophenone (**3–7** and **11–13**) in methanol (MeOH) or ethanol (EtOH) was added barium hydroxide (2.0 eq) and appropriate aldehyde (1.2 eq) at room temperature. The reaction mixture was stirred under argon at 40–50 °C until complete conversion monitored by TLC analysis (typically 1–20 h), quenched with acetic acid, and extracted with EtOAc and H_2_O. The organic layer was washed with saturated aqueous NaHCO_3_, dried over MgSO_4_, filtered, and concentrated under reduced pressure. The crude residue was purified by column chromatography on a silica gel to furnish compounds **8a–8n** and **14a–14d**.

##### (E)-1–(4-Fluoro-2-hydroxyphenyl)-3-phenylprop-2-en-1-one (8a)

Compound **8a** was prepared in 72% yield as a yellow powder, following the same procedure as described in the general procedure A with *1–(4-Fluoro-2-hydroxyphenyl)ethan-1-one* (**3**) (200 mg, 1.3 mmol), benzaldehyde (159 μL, 1.2 eq), barium hydroxide (446 mg, 2.0 eq) in methanol (10 mL), stirring for 1 h at 50 °C. The crude residue was purified by column chromatography on silica gel (petroleum ether/ether = 50:1 to 20:1). R_f_ = 0.34 (petroleum ether/ether = 20:1). ^1^H NMR (600 MHz, CDCl_3_) *δ* 13.2 (s, 1H), 7.97 − 7.90 (m, 2H), 7.70 − 7.64 (m, 2H), 7.58 (d, *J* = 15.6 Hz, 1H), 7.48 − 7.43 (m, 3H), 6.71 (dd, *J* = 2.6 and 10.3 Hz, 1H), 6.69 − 6.65 (m, 1H).

##### (E)-1–(4-Fluoro-2-hydroxyphenyl)-3–(4-methoxyphenyl)prop-2-en-1-one (8b)

Compound **8b** was prepared in 44% yield as a yellow powder, following the same procedure as described in the general procedure A with *1–(4-Fluoro-2-hydroxyphenyl)ethan-1-one* (**3**) (200 mg, 1.30 mmol), p-anisaldehyde (190 μL, 1.2 eq), barium hydroxide (445 mg, 2.0 eq) in methanol (10 mL), stirring for 17 h at 50 °C. The crude residue was purified by column chromatography on silica gel (hexane/EtOAc = 10:1 to 1:1). R_f_ = 0.50 (hexane/EtOAc = 4:1). ^1^H NMR (600 MHz, CDCl_3_) *δ* 13.33 (s, 1H), 7.96 − 7.89 (m, 2H), 7.63 (d, *J* = 8.7 Hz, 2H), 7.45 (d, *J* = 15.3 Hz, 1H), 6.96 (d, *J* = 8.6 Hz, 2H), 6.70 (dd, *J* = 2.5 and 10.3 Hz, 1H), 6.68 − 6.63 (m, 1H), 3.87 (s, 3H).

##### (E)-1–(5-Fluoro-2-hydroxyphenyl)-3-phenylprop-2-en-1-one (8c)

Compound **8c** was prepared in quantitative yield as a yellow powder, following the same procedure as described in the general procedure A with *1–(5-Fluoro-2-hydroxyphenyl)ethan-1-one* (**4**) (200 mg, 1.3 mmol), benzaldehyde (159 μL, 1.2 eq), barium hydroxide (446 mg, 2.0 eq) in methanol (8 mL), stirring for 1 h at 50 °C. The crude residue was purified by column chromatography on silica gel (petroleum ether/ether = 50:1 to 20:1). R_f_ = 0.45 (petroleum ether/ether = 20:1). ^1^H NMR (600 MHz, CDCl_3_) *δ* 12.53 (s, 1H), 7.95 (d, *J* = 15.4 Hz, 1H), 7.72 − 7.66 (m, 2H), 7.59 (dd, *J* = 3.0 and 9.0 Hz, 1H), 7.55 (d, *J* = 15.4 Hz, 1H), 7.47 − 7.44 (m, 3H), 7.29 − 7.22 (m, 2H), 7.0 (dd, *J* = 4.6 and 9.1 Hz, 1H).

##### (E)-1–(5-Fluoro-2-hydroxyphenyl)-3–(4-methoxyphenyl)prop-2-en-1-one (8d)

Compound **8d** was prepared in 76% yield as a yellow powder, following the same procedure as described in the general procedure A with *1–(4-Fluoro-2-hydroxyphenyl)ethan-1-one* (**4**) (200 mg, 1.30 mmol), p-anisaldehyde (190 μL, 2 eq), barium hydroxide (445 mg, 2.0 eq) in methanol (10 mL), stirring for 4 h at 40 °C. The crude residue was purified by column chromatography on silica gel (hexane/EtOAc = 8:1 to 4:1). R_f_ = 0.56 (hexane/EtOAc = 1:1). ^1^H NMR (600 MHz, CDCl_3_) *δ* 12.65 (s, 1H), 7.93 (d, *J* = 15.3 Hz, 1H), 7.65 (d, *J* = 8.6 Hz, 2H), 7.58 (dd, *J* = 2.9 and 9.1 Hz, 1H), 7.43 (d, *J* = 15.3 Hz, 1H), 7.25 − 7.21 (m, 1H), 7.02 − 6.94 (m, 3H), 3.88 (s, 3H).

##### (E)-1–(2-Fluoro-6-hydroxyphenyl)-3-phenylprop-2-en-1-one (8e)

Compound **8e** was prepared in quantitative yield as a yellow powder, following the same procedure as described in the general procedure A with *1–(2-Fluoro-6-hydroxyphenyl)ethan-1-one* (**5**) (166 μL, 1.3 mmol), benzaldehyde (159 μL, 1.2 eq), barium hydroxide (446 mg, 2.0 eq) in methanol (8 mL), stirring for 3 h at 50 °C. The crude residue was purified by column chromatography on silica gel (petroleum ether/ether = 50:1 to 20:1). R_f_ = 0.67 (hexane/EtOAc = 5:1). ^1^H NMR (600 MHz, CDCl_3_) *δ* 7.94 (dd, *J* = 3.5 and 16.0 Hz, 1H), 7.72 − 7.64 (m, 3H), 7.49 − 7.38 (m, 4H), 6.58 (d, *J* = 8.5 Hz, 1H), 6.71 − 6.61 (m, 1H); ^13^C NMR (150 MHz, CDCl_3_) *δ* 192.49, 164.68, 163.74, 162.05, 145.72, 136.07, 135.98, 134.66, 131.00, 129.04, 128.86, 125.39, 125.28, 114.47, 106.40, 106.23. HRMS *m*/*z* calculated for C_15_H_11_FO_2_ [M – H]^–^: 241.0670; found: 241.0681.

##### (E)-1–(2-Fluoro-6-hydroxyphenyl)-3–(4-methoxyphenyl)prop-2-en-1-one (8f)

Compound **8f** was prepared in quantitative yield as a yellow powder, following the same procedure as described in the general procedure A with *1–(2-Fluoro-6-hydroxyphenyl)ethan-1-one* (**5**) (200 mg, 1.30 mmol), p-anisaldehyde (190 μL, 1.2 eq), barium hydroxide (445 mg, 2.0 eq) in methanol (10 mL), stirring for 6 h at 50 °C. The crude residue was purified by column chromatography on silica gel (hexane/EtOAc = 10:1 to 4:1). R_f_ = 0.33 (hexane/EtOAc = 4:1). ^1^H NMR (600 MHz, CDCl_3_) *δ* 13.17 (s, 1H), 7.93 (dd, *J* = 3.4 and 15.2 Hz, 1H), 7.62 (d, *J* = 8.7 Hz, 2H), 7.56 (dd, *J* = 1.8 and 15.4 Hz, 1H), 7.42 − 7.36 (m, 1H), 6.95 (d, *J* = 8.7 Hz, 2H), 6.81 (d, *J* = 8.4 Hz, 1H), 6.63 (dd, *J* = 8.3 and 12.1 Hz, 1H), 3.87 (s, 3H); ^13^C NMR (150 MHz, CDCl_3_) *δ* 192.35, 164.68, 163.72, 162.13, 162.02, 145.77, 135.75, 135.67, 130.80, 130.78, 127.45, 122.99, 122.88, 114.55, 114.53, 114.40, 110.73, 110.63, 106.34, 106.18, 55.47. HRMS *m*/*z* calculated for C_16_H_16_F_1_O_3_ [M – H]^–^: 271.0776; found: 271.0780.

##### (E)-1–(4,5-Difluoro-2-hydroxyphenyl)-3-phenylprop-2-en-1-one (8g)

Compound **8g** was prepared in 84% yield as a yellow powder, following the same procedure as described in the general procedure A with *1–(4,5-Difluoro-2-hydroxyphenyl)ethan-1-one* (**6**) (200 mg, 1.16 mmol), benzaldehyde (142 μL, 1.2 eq), barium hydroxide (398 mg, 2.0 eq) in methanol (8 mL), stirring for 4 h at 50 °C. The crude residue was purified by column chromatography on silica gel (hexane/EtOAc = 12:1 to 6:1). R_f_ = 0.64 (hexane/EtOAc = 4:1). ^1^H NMR (600 MHz, CDCl_3_) *δ* 7.96 (d, *J* = 15.4 Hz, 1H), 7.76 − 7.70 (m, 1H), 7.69 − 7.65 (m, 2H), 7.51 − 7.42 (m, 4H), 6.83 (dd, *J* = 6.5 and 11.4 Hz, 1H); ^13^C NMR (150 MHz, CDCl_3_) *δ* 192.02, 161.41, 156.36, 156.27, 146.61, 134.23, 131.35, 129.16, 128.83, 119.36, 116.97, 116.95, 116.85, 107.11, 106.99. HRMS *m*/*z* calculated for C_15_H_10_F_2_O_2_ [M – H]^–^: 259.0576; found: 259.0587.

##### (E)-1–(4,5-Difluoro-2-hydroxyphenyl)-3–(4-methoxyphenyl)prop-2-en-1-one (8h)

Compound **8h** was prepared in 83% yield as a yellow powder, following the same procedure as described in the general procedure A with *1–(4,5-Difluoro-2-hydroxyphenyl)ethan-1-one* (**6**) (200 mg, 1.16 mmol), p-anisaldehyde (169 μL, 1.2 eq), barium hydroxide (398 mg, 2.0 eq) in methanol (8 mL), stirring for 4 h at 50 °C. The crude residue was purified by column chromatography on silica gel (hexane/EtOAc = 10:1 to 4:1). R_f_ = 0.43 (hexane/EtOAc = 4:1). ^1^H NMR (600 MHz, CDCl_3_) *δ* 13.09 (s, 1H), 7.93 (d, *J* = 15.1 Hz, 1H), 7.71 (dd, *J* = 8.6 and 10.8 Hz, 1H), 7.64 (d, *J* = 8.6 Hz, 2H), 7.34 (d, *J* = 15.4 Hz, 1H), 6.97 (d, *J* = 8.6 Hz, 2H), 6.81 (dd, *J* = 7.1 and 11.7 Hz, 1H), 3.88 (s, 3H); ^13^C NMR (150 MHz, CDCl_3_) *δ* 192.02, 161.41, 156.36, 156.27, 146.61, 134.23, 131.35, 129.16, 12.83, 119.36, 116.97, 116.95, 116.5, 107.11, 106.9. HRMS *m*/*z* calculated for C_16_H_12_F_2_O_3_ [M – H]^–^: 289.0682; found: 289.0695.

##### (E)-Ethyl 3–(3-(4,5-difluoro-2-hydroxyphenyl)-3-oxoprop-1-en-1-yl)benzoate (8i)

Compound **8i** was prepared in 64% yield as a yellow powder, following the same procedure as described in the general procedure A with *1–(4,5-Difluoro-2-hydroxyphenyl)ethan-1-one* (**6**) (150 mg, 0.87 mmol), methyl 3-formylbenzoate (172 mg, 1.2 eq), barium hydroxide (298 mg, 2.0 eq) in ethanol (10 mL), stirring for 16 h at 50 °C. The crude residue was purified by column chromatography on silica gel (CH_2_Cl_2_/MeOH = 500:1 to 5:1). R_f_ = 0.56 (hexane/EtOAc = 4:1). ^1^H NMR (600 MHz, CDCl_3_) *δ* 13.08 (s, 1H), 7.94 (d, *J* = 15.1 Hz, 2H), 7.71 (dd, *J* = 2.2 and 8.6 Hz, 1H), 7.64 (d, *J* = 8.64 Hz, 2H), 7.34 (d, *J* = 15.4 Hz, 1H), 6.98 (d, *J* = 8.64 Hz, 2H), 6.81 (dd, *J* = 4.6 and 7.1 Hz, 1H), 3.88 (s, 3H). HRMS *m*/*z* calculated for C_18_H_14_F_2_O_4_ [M – H]^–^: 331.0787; found: 331.0801.

##### (E)-1–(2,4-Difluoro-6-hydroxyphenyl)-3-phenylprop-2-en-1-one (8j)

Compound **8j** was prepared in 95% yield as a yellow powder, following the same procedure as described in the general procedure A with *1–(2,4-Difluoro-6-hydroxyphenyl)ethan-1-one* (**7**) (200 mg, 1.16 mmol), benzaldehyde (142 μL, 1.2 eq), barium hydroxide (398 mg, 2.0 eq) in methanol (8 mL), stirring for 2 h at 50 °C. The crude residue was used in the next step without further purification. R_f_ = 0.74 (hexane/EtOAc = 4:1). ^1^H NMR (600 MHz, CDCl_3_) *δ* 7.98 (dd, *J* = 3.3 and 15.4 Hz, 1H), 7.71 − 7.66 (m, 2H), 7.64 (dd, *J* = 2.2 and 15.4 Hz, 1H), 7.50 − 7.44 (m, 3H), 6.59 − 6.53 (m, 1H), 6.47 − 6.41 (m, 1H); ^13^C NMR (150 MHz, CDCl_3_) *δ* 191.64, 191.60, 167.64, 166.78, 166.71, 165.09, 164.98, 163.39, 163.28, 146.20, 134.53, 131.13, 129.07, 128.88, 124.78, 124.67, 101.59, 101.43, 101.41, 96.27, 96.08, 95.90. HRMS *m*/*z* calculated for C_15_H_10_F_2_O_2_ [M – H]^–^: 259.0576; found: 259.0587.

##### (E)-1–(2,4-Difluoro-6-hydroxyphenyl)-3–(4-methoxyphenyl)prop-2-en-1-one (8k)

Compound **8k** was prepared in 85% yield as a yellow powder, following the same procedure as described in the general procedure A with *1–(2,4-Difluoro-6-hydroxyphenyl)ethan-1-one* (**7**) (200 mg, 1.16 mmol), p-anisaldehyde (169 μL, 1.2 eq), barium hydroxide (398 mg, 2.0 eq) in methanol (8 mL), stirring for 3 h at 50 °C. The crude residue was purified by column chromatography on silica gel (hexane/EtOAc = 10:1 to 6:1). R_f_ = 0.60 (hexane/EtOAc = 4:1). ^1^H NMR (600 MHz, CDCl_3_) *δ* 7.94 (dd, *J* = 3.5 and 15.4 Hz, 1H), 7.62 (d, *J* = 8.7 Hz, 2H), 7.50 (dd, *J* = 1.9 and 15.3 Hz, 1H), 6.95 (d, *J* = 8.8 Hz, 2H), 6.55 − 6.50 (m, 1H), 6.43 − 6.36 (m, 1H), 3.87 (s, 3H); ^13^C NMR (150 MHz, DMSO) *δ* 191.39, 162.23, 146.22, 130.82, 127.30, 122.29, 122.17, 114.55, 101.47, 101.34, 96.13, 95.94, 55.46. HRMS *m*/*z* calculated for C_16_H_12_F_2_O_5_ [M – H]^–^: 289.0682; found: 289.0696.

##### (E)-Ethyl 4–(3-(2-hydroxy-4,5-dimethoxyphenyl)-3-oxoprop-1-en-1-yl)benzoate (14a)

Compound **14a** was prepared in 70% yield as a yellow powder, following the same procedure as described in the general procedure A with *1–(2-Hydroxy-4,5-dimethoxyphenyl)ethan-1-one* (**11**) (200 mg, 1.02 mmol), methyl terephthalaldehydate (251 mg, 1.5 eq), barium hydroxide (350 mg, 2.0 eq) in ethanol (10 mL), stirring for 17 h at 70 °C. The crude residue was purified by column chromatography on silica gel (hexane/EtOAc = 8:1 to 4:1). R_f_ = 0.21 (hexane/EtOAc = 4:1). ^1^H NMR (600 MHz, CDCl_3_) *δ* 13.30 (s, 1H), 8.09 (d, *J* = 8.2 Hz, 2H), 7.88 (d, *J* = 15.4 Hz, 1H), 7.70 (d, *J* = 8.2 Hz, 2H), 7.57 (d, *J* = 15.5 Hz, 1H), 7.24 (s, 1H), 6.50 (s, 1H), 4.40 (q, *J* = 7.1 Hz, 2H), 3.93 (s, 3H), 3.92 (s, 3H), 1.41 (t, *J* = 7.1 Hz, 3H); ^13^C NMR (150 MHz, CDCl_3_) *δ* 191.01, 165.91, 162.01, 157.38, 142.93, 142.07, 138.86, 131.98, 130.12, 128.30, 122.46, 111.93, 110.88, 100.84, 61.26, 56.94, 56.24, 14.31. HRMS *m*/*z* calculated for C_20_H_20_O_6_ [M – H]^–^: 355.1187; found: 355.1203.

#### (E)-Ethyl 3–(3-(2-hydroxy-4,5-dimethoxyphenyl)-3-oxoprop-1-en-1-yl)benzoate (14b)

Compound **14b** was prepared in 26% yield as a yellow powder, following the same procedure as described in the general procedure A with *1–(2-Hydroxy-4,5-dimethoxyphenyl)ethan-1-one* (**11**) (200 mg, 1.02 mmol), methyl 3-formylbenzoate (200 mg, 1.2 eq), barium hydroxide (350 mg, 2.0 eq) in ethanol (10 mL), stirring for 20 h at 40 °C. The crude residue was purified by column chromatography on silica gel (hexane/EtOAc = 8:1 to 3:1). R_f_ = 0.28 (hexane/EtOAc = 4:1). ^1^H NMR (600 MHz, CDCl_3_) *δ* 13.33 (s, 1H), 8.35 (s, 1H), 8.09 (d, *J* = 7.7 Hz, 1H), 7.93 (d, *J* = 15.5 Hz, 1H), 7.81 (d, *J* = 7.7 Hz, 1H), 7.58 (d, *J* = 15.5 Hz, 1H), 7.52 (t, *J* = 7.7 Hz, 1H), 7.27 (d, *J* = 4.5 Hz, 2H), 6.53 (s, 1H), 4.43 (q, *J* = 7.1 Hz, 2H), 3.95 (s, 3H), 3.94 (s, 3H), 1.43 (t, *J* = 7.1 Hz, 3H); ^13^C NMR (150 MHz, CDCl_3_) *δ* 191.21, 166.08, 161.96, 157.32, 143.31, 142.07, 135.13, 132.88, 131.36, 131.32, 129.17, 129.11, 121.53, 111.95, 111.01, 100.86, 61.39, 57.04, 56.25, 14.34. HRMS *m*/*z* calculated for C_20_H_20_O_6_ [M + H]^+^: 357.1333; found: 357.1333.

##### (E)-Ethyl 4–(3-(2-hydroxy-4,6-dimethoxyphenyl)-3-oxoprop-1-en-1-yl)benzoate (14c)

Compound **14c** was prepared in 16% yield as a yellow powder, following the same procedure as described in the general procedure A with *1–(2-Hydroxy-4,6-dimethoxyphenyl)ethan-1-one* (**12**) (200 mg, 1.02 mmol), methyl terephthalaldehydate (200 mg, 1.2 eq), barium hydroxide (350 mg, 2.0 eq) in ethanol (10 mL), stirring for 18 h at 40 °C. The crude residue was purified by column chromatography on silica gel (hexane/EtOAc = 8:1 to 3:1). R_f_ = 0.24 (hexane/EtOAc = 4:1). ^1^H NMR (600 MHz, CDCl_3_) *δ* 14.18 (s, 1H), 8.08 (d, *J* = 8.3 Hz, 2H), 7.95 (d, *J* = 15.6 Hz, 1H), 7.76 (d, *J* = 15.6 Hz, 1H), 7.65 (d, *J* = 8.2 Hz, 2H), 6.12 (d, *J* = 1.9 Hz, 1H), 5.98 (d, *J* = 2.3 Hz, 1H), 4.40 (q, *J* = 7.9 and 15.1 Hz, 2H), 3.93 (s, 3H), 3.85 (s, 3H), 1.41 (t, *J* = 7.1 Hz, 3H); ^13^C NMR (150 MHz, MeOD) *δ* 192.29, 168.48, 166.52, 166.12, 162.52, 140.61, 139.80, 131.40, 130.06, 129.76, 128.07, 106.33, 93.83, 91.37, 61.18, 55.93, 55.65, 14.32. HRMS *m*/*z* calculated for C_20_H_20_O_6_ [M + H]^+^: 357.1333; found: 357.1343.

##### (E)-Ethyl 3–(3-(2-hydroxy-4,6-dimethoxyphenyl)-3-oxoprop-1-en-1-yl)benzoate (14d)

Compound **14d** was prepared in 83% yield as a yellow powder, following the same procedure as described in the general procedure A with *1–(2-Hydroxy-4,6-dimethoxyphenyl)ethan-1-one* (**12**) (200 mg, 1.02 mmol), methyl 3-formylbenzoate (200 mg, 1.2 eq), barium hydroxide (350 mg, 2.0 eq) in ethanol (10 mL), stirring for 17 h at 40 °C. The crude residue was purified by column chromatography on silica gel (CH_2_Cl_2_/MeOH = 200:1 to 100:1). R_f_ = 0.35 (hexane/EtOAc = 4:1). ^1^H NMR (600 MHz, CDCl_3_) *δ* 14.22 (s, 1H), 8.30 (s, 1H), 8.05 (d, *J* = 7.2 Hz, 1H), 7.97 (d, *J* = 15.6 Hz, 1H), 7.82 − 7.74 (m, 2H), 7.49 (t, *J* = 7.9 Hz, 1H), 6.12 (d, *J* = 2.0 Hz, 1H), 5.98 (d, *J* = 2.1 Hz, 1H), 4.41 (q, *J* = 7.1 Hz, 2H), 3.94 (s, 3H), 3.85 (s, 3H), 1.43 (t, *J* = 7.1 Hz, 3H); ^13^C NMR (150 MHz, CDCl_3_) *δ* 192.40, 1686.47, 166.43, 166.15, 162.53, 140.89, 135.93, 132.48, 131.19, 130.73, 129.12, 128.95, 128.85, 128.76, 93.82, 91.32, 61.23, 55.88, 55.63, 14.33. HRMS *m*/*z* calculated for C_20_H_20_O_6_ [M + H]^+^: 357.1333; found: 357.1328.

#### *General* Procedure B *for compounds 9a–9k and 15a–15d*

To a stirred solution of chalcone compound (**8a–8k** and **14a–14d**.) in dimethylsulphoxide (DMSO) was added iodine (0.1 eq) at 25 °C. The reaction mixture was stirred under argon at 110 °C until complete conversion monitored by TLC analysis (typically 6–24h), quenched with 1M sodium thiosulphate solution, and extracted with EtOAc, and H_2_O. The organic layer was dried over MgSO_4_, filtered, and concentrated under reduced pressure. The crude residue was purified by column chromatography on a silica gel (hexane/EtOAc or CH_2_Cl_2_/MeOH)to furnish compounds **9a–9k** and **15a–15d**.

##### 7-Fluoro-2-phenyl-4H-chromen-4-one (9a)

Compound **9a** was prepared in 90% yield as a white powder, following the same procedure as described in the general procedure B *(E)-1–(4-Fluoro-2-hydroxyphenyl)-3-phenylprop-2-en-1-one* (**8a**) (194 mg, 0.80 mmol), Iodine (20.3 mg, 0.1 eq) in DMSO (6 mL), stirring for 24 h. The crude residue was purified by column chromatography on silica gel (hexane/EtOAc = 10:1 to 3:1). R_f_ = 0.27 (hexane/EtOAc = 4:1). m.p: 96–98 °C. ^1^H NMR (600 MHz, CDCl_3_) *δ* 8.26 (dd, *J* = 6.4 and 8.8 Hz, 1H), 7.91 (dd, *J* = 1.3 and 7.8 Hz, 2H), 7.60 − 7.51 (m, 3H), 7.28 (d, *J* = 2.2 Hz, 1H), 7.20 − 7.14 (m, 1H), 6.82 (s, 1H); ^13^C NMR (150 MHz, CDCl_3_) *δ* 177.48, 166.60, 164.91, 163.75, 157.31, 157.23, 131.80, 131.46, 129.14, 128.30, 126.28, 120.87, 114.09, 113.94, 107.69, 104.92, 104.75. HRMS *m*/*z* calculated for C_15_H_9_FO_2_ [M + H]^+^: 241.0660; found: 241.0654. >95% purity (as determined by RP-HPLC, method C, *t*_R_ = 11.30 min).

##### 7-Fluoro-2–(4-methoxyphenyl)-4H-chromen-4-one (9b)

Compound **9b** was prepared in quantitative yield as a white powder, following the same procedure as described in the general procedure B with *(E)-1–(4-Fluoro-2-hydroxyphenyl)-3–(4-methoxyphenyl)prop-2-en-1-one* (**8b**) (137 mg, 0.50 mmol), iodine (12.7 mg, 0.1 eq) in DMSO (7 mL), stirring for 16 h. The crude residue was purified by column chromatography on silica gel (hexane/EtOAc = 5:1 to 1:1 and then CH_2_Cl_2_/MeOH = 10:1). R_f_ = 0.40 (hexane/EtOAc = 1:1). m.p: 228–230 °C. ^1^H NMR (600 MHz, CDCl_3_) *δ* 8.24 (dd, *J* = 6.4 and 8.8 Hz, 1H), 7.87 (d, *J* = 8.9 Hz, 2H), 7.24 (dd, *J* = 2.3 and 9.1 Hz, 1H), 7.17 − 7.12 (m, 1H), 7.04 (d, *J* = 8.9 Hz, 2H), 6.73 (s, 1H), 3.90 (s, 3H); ^13^C NMR (150 MHz, CDCl_3_) *δ* 177.45, 166.49, 164.80, 163.77, 162.57, 157.22, 128.21, 128.14, 128.01, 123.67, 120.82, 114.56, 113.88, 113.74, 106.21, 104.80, 104.64, 55.55. HRMS *m*/*z* calculated for C_16_H_11_FO_3_ [M + H]^+^: 271.0765; found: 271.0753. >95% purity (as determined by RP-HPLC, method B, *t*_R_ = 7.28 min).

##### 6-Fluoro-2-phenyl-4H-chromen-4-one (9c)

Compound **9c** was prepared in 91% yield as a white powder, following the same procedure as described in the general procedure B with *(E)-1–(5-Fluoro-2-hydroxyphenyl)-3-phenylprop-2-en-1-one* (**8c**) (304 mg, 1.25 mmol), iodine (31.7 mg, 0.1 eq) in DMSO (6 mL), stirring for 15 h. The crude residue was purified by column chromatography on silica gel (hexane/EtOAc = 10:1 to 1:1). R_f_ = 0.46 (hexane/EtOAc = 4:1). m.p: 131–133 °C. ^1^H NMR (600 MHz, CDCl_3_) *δ* 7.93 (d, *J* = 7.1 Hz, 2H), 7.88 (dd, *J* = 3.2 and 8.1 Hz, 1H), 7.61 − 7.58 (m, 1H), 7.57 − 7.52 (m, 3H), 7.46 − 7.41 (m, 1H), 6.83 (s, 1H); ^13^C NMR (150 MHz, CDCl_3_) *δ* 177.63, 163.71, 160.44, 158.81, 152.47, 131.82, 131.54, 129.12, 126.34, 125.21, 125.16, 122.01, 121.85, 120.22, 120.16, 110.74, 110.59, 106.92. HRMS *m*/*z* calculated for C_15_H_9_FO_2_ [M + H]^+^: 241.0660; found: 241.0648. >95% purity (as determined by RP-HPLC, method C, *t*_R_ = 11.20 min).

##### 6-Fluoro-2–(4-methoxyphenyl)-4H-chromen-4-one (9d)

Compound **9d** was prepared in 97% yield as a white powder, following the same procedure as described in the general procedure B with *(E)-1–(5-Fluoro-2-hydroxyphenyl)-3–(4-methoxyphenyl)prop-2-en-1-one* (**8d**) (240 mg, 0.88 mmol), iodine (22.3 mg, 0.1 eq) in DMSO (10 mL), stirring for 15 h. The crude residue was purified by column chromatography on silica gel (hexane/EtOAc = 6:1 to 1:1). R_f_ = 0.23 (hexane/EtOAc = 4:1). m.p: 160–162 °C. ^1^H NMR (600 MHz, CDCl_3_) *δ* 7.90 − 7.85 (m, 3H), 7.56 (dd, *J* = 4.1 and 9.1 Hz, 1H), 7.44 − 7.39 (m, 1H), 7.04 (d, *J* = 8.9 Hz, 2H), 6.74 (s, 1H), 3.90 (s, 3H); ^13^C NMR (150 MHz, CDCl_3_) *δ* 177.55, 163.72, 162.58, 160.38, 158.74, 152.39, 128.07, 125.18, 123.75, 121.74, 121.57, 120.04, 119.99, 114.54, 110.72, 110.57, 105.52, 55.54. HRMS *m*/*z* calculated for C_16_H_11_FO_3_ [M + H]^+^: 271.0765; found: 271.0751. >95% purity (as determined by RP-HPLC, method B, *t*_R_ = 7.12 min).

##### 5-Fluoro-2-phenyl-4H-chromen-4-one (9e)

Compound **9e** was prepared in 86% yield as a white powder, following the same procedure as described in the general procedure B with *(E)-1–(2-Fluoro-6-hydroxyphenyl)-3-phenylprop-2-en-1-one* (**8e**) (200 mg, 0.83 mmol), iodine (21.1 mg, 0.1 eq) in DMSO (5 mL), stirring for 20 h. The crude residue was purified by column chromatography on silica gel (hexane/EtOAc = 10:1 to 1:1). R_f_ = 0.27 (hexane/EtOAc = 4:1). m.p: 144–146 °C. ^1^H NMR (600 MHz, CDCl_3_) *δ* 7.91 (d, *J* = 7.8 Hz, 2H), 7.66 − 7.61 (m, 1H), 7.58 − 7.51 (m, 3H), 7.39 (d, *J* = 8.5 Hz, 1H), 7.12 − 7.05 (m, 1H), 6.78 (s, 1H); ^13^C NMR (150 MHz, CDCl_3_) *δ* 176.79, 162.46, 133.74, 133.67, 131.81, 131.23, 129.13, 126.27, 114.06, 114.03, 112.23, 112.09, 108.69. HRMS *m*/*z* calculated for C_15_H_9_FO_2_ [M + H]^+^: 241.0660; found: 241.0647. >95% purity (as determined by RP-HPLC, method C, *t*_R_ = 7.86 min).

##### 5-Fluoro-2–(4-methoxyphenyl)-4H-chromen-4-one (9f)

Compound **9f** was prepared in quantitative yield as a white powder, following the same procedure as described in the general procedure B with *(E)-1–(2-Fluoro-6-hydroxyphenyl)-3–(4-methoxyphenyl)prop-2-en-1-one* (**8f**) (380 mg, 1.40 mmol), iodine (35.5 mg, 0.1 eq) in DMSO (10 mL), stirring for 16 h. The crude residue was purified by column chromatography on silica gel (hexane/EtOAc = 5:1 to 1:1 and then CH_2_Cl_2_/MeOH = 10:1). R_f_ = 0.40 (hexane/EtOAc = 1:1). m.p: 178–180 °C. ^1^H NMR (600 MHz, CDCl_3_) *δ* 7.87 − 7.84 (m, 2H), 7.63 − 7.58 (m, 1H), 7.36 (d, *J* = 8.5 Hz, 1H), 7.08 − 7.04 (m, 1H), 7.04 − 7.01 (m, 2H), 6.69 (s, 1H), 3.90 (s, 3H); ^13^C NMR (150 MHz, CDCl_3_) *δ* 176.63, 162.52, 162.35, 161.47, 159.72, 157.14, 157.12, 133.50, 133.43, 127.88, 123.20, 114.48, 114.28, 114.21, 113.94, 113.91, 112.02, 111.88, 107.04, 55.50. HRMS *m*/*z* calculated for C_16_H_11_FO_3_ [M + H]^+^: 271.0765; found: 271.0773. >95% purity (as determined by RP-HPLC, method C, *t*_R_ = 7.46 min).

##### 6,7-Difluoro-2-phenyl-4H-chromen-4-one (9g)

Compound **9 g** was prepared in 88% yield as a white powder, following the same procedure as described in the general procedure B with *(E)-1–(4,5-Difluoro-2-hydroxyphenyl)-3-phenylprop-2-en-1-one* (**8 g**) (145 mg, 0.56 mmol), iodine (14.1 mg, 0.1 eq) in DMSO (8 mL), stirring for 14 h. The crude residue was purified by column chromatography on silica gel (hexane/EtOAc = 10:1 to 4:1). R_f_ = 0.38 (hexane/EtOAc = 4:1). m.p: 182–184 °C. ^1^H NMR (600 MHz, CDCl_3_) *δ* 8.04 − 7.98 (m, 1H), 7.93 − 7.87 (m, 2H), 7.59 − 7.52 (m, 3H), 7.42 (dd, *J* = 6.1 and 9.8 Hz, 1H), 6.81 (s, 1H); ^13^C NMR (150 MHz, CDCl_3_) *δ* 176.68, 164.06, 154.93, 154.82, 153.21, 153.11, 152.49, 152.42, 149.49, 149.40, 147.83, 147.74, 131.98, 131.21, 129.18, 126.27, 120.97, 120.95, 113.14, 113.13, 113.02, 113.01, 107.30, 107.15, 107.09. HRMS *m*/*z* calculated for C_15_H_8_F_2_O_2_ [M – H]^–^: 259.0565; found: 259.0564. >95% purity (as determined by RP-HPLC, method C, *t*_R_ = 14.19 min).

##### 6,7-Difluoro-2–(4-methoxyphenyl)-4H-chromen-4-one (9h)

Compound **9h** was prepared in quantitative yield as a white powder, following the same procedure as described in the general procedure B with *(E)-1–(4,5-Difluoro-2-hydroxyphenyl)-3–(4-methoxyphenyl)prop-2-en-1-one* (**8h**) (220 mg, 0.76 mmol), iodine (19.2 mg, 0.1 eq) in DMSO (7 mL), stirring for 15 h. The crude residue was purified by column chromatography on silica gel (hexane/EtOAc = 5:1 to 1:1 and then CH_2_Cl_2_/MeOH = 10:1). R_f_ = 0.20 (hexane/EtOAc = 4:1). m.p: 200–202 °C. ^1^H NMR (600 MHz, DMSO) *δ* 8.10 − 8.02 (m, 3H), 7.93 (t, *J* = 9.2 Hz, 1H), 7.12 (d, *J* = 8.9 Hz, 2H), 6.99 (s, 1H), 3.85 (s, 3H); ^13^C NMR (150 MHz, DMSO) *δ* 176.05, 163.81, 162.85, 152.69, 128.80, 123.28, 121.12, 115.13, 112.65, 112.52, 108.87, 108.73, 105.39, 56.07. HRMS *m*/*z* calculated for C_16_H_10_F_2_O_3_ [M + H]^+^: 289.0671; found: 289.0656. >95% purity (as determined by RP-HPLC, method B, *t*_R_ = 8.87 min).

##### Ethyl 3–(6,7-difluoro-4-oxo-4H-chromen-2-yl)benzoate (9i)

Compound **9i** was prepared in quantitative yield as a white powder, following the same procedure as described in the general procedure B with *(E)-Ethyl 3–(3-(4,5-difluoro-2-hydroxyphenyl)-3-oxoprop-1-en-1-yl)benzoate* (**8i**) (155 mg, 0.47 mmol), iodine (22.8 mg, 0.2 eq) in DMSO (8 mL), stirring for 6 h. The crude residue was purified by column chromatography on silica gel (CH_2_Cl_2_/MeOH = 50:1 to 20:1). R_f_ = 0.40 (hexane/EtOAc = 4:1). ^1^H NMR (600 MHz, CDCl_3_) *δ* 8.59 − 8.55 (m, 1H), 8.26 − 8.21 (m, 1H), 8.09 − 8.05 (m, 1H), 8.04 − 7.99 (m, 1H), 7.63 (t, *J* = 7.8 Hz, 1H), 7.47 (dd, *J* = 6.2 and 9.8 Hz, 1H), 6.87 (s, 1H)., 4.46 (q, *J* = 7.1 Hz, 2H), 1.45 (t, *J* = 7.1 Hz, 3H); ^13^C NMR (150 MHz, CDCl_3_) *δ* 176.60, 165.59, 163.02, 155.05, 154.95, 153.33, 153.23, 152.48, 152.41, 149.61, 149.52, 147.94, 147.86, 132.72, 131.67, 131.62, 130.20, 129.37, 127.39, 120.93, 113.20, 113.19, 113.07, 107.61, 107.44, 107.29, 61.63, 14.34. HRMS *m*/*z* calculated for C_18_H_12_F_2_O_4_ [M + H]^+^: 331.0777; found: 331.0791.

##### 6,7-Difluoro-2-phenyl-4H-chromen-4-one (9j)

Compound **9j** was prepared in 88% yield as a white powder, following the same procedure as described in the general procedure B with *(E)-1–(2,4-Difluoro-6-hydroxyphenyl)-3-phenylprop-2-en-1-one* (**8j**) (280 mg, 0.96 mmol), iodine (24.4 mg, 0.1 eq) in DMSO (10 mL), stirring for 14 h. The crude residue was purified by column chromatography on silica gel (hexane/EtOAc = 10:1 to 1:1). R_f_ = 0.25 (hexane/EtOAc = 4:1). m.p: 158–160 °C. ^1^H NMR (600 MHz, CDCl_3_) *δ* 7.88 (d, *J* = 7.0 Hz, 2H), 7.59 − 7.51 (m, 3H), 7.10 (d, *J* = 8.8 Hz, 1H), 6.88 − 6.83 (m, 1H), 6.75 (s, 1H); ^13^C NMR (150 MHz, CDCl_3_) *δ* 175.75, 165.74, 165.64, 164.05, 163.95, 162.78, 162.68, 162.56, 161.01, 160.91, 158.07, 158.03, 157.96, 157.93, 131.97, 130.78, 129.17, 126.18, 111.58, 111.53, 111.51, 108.62, 102.19, 102.03, 101.86, 101.41, 101.38, 101.25, 101.22. HRMS *m*/*z* calculated for C_15_H_8_F_2_O_2_ [M – H]^–^: 259.0565; found: 259.0562. >95% purity (as determined by RP-HPLC, method C, *t*_R_ = 9.89 min).

##### 5,7-Difluoro-2–(4-methoxyphenyl)-4H-chromen-4-one (9k)

Compound **9k** was prepared in 47% yield as a white powder, following the same procedure as described in the general procedure B with *(E)-1–(2,4-Difluoro-6-hydroxyphenyl)-3–(4-methoxyphenyl)prop-2-en-1-one* (**8k**) (270 mg, 0.93 mmol), iodine (23.6 mg, 0.1 eq) in DMSO (6 mL), stirring for 10 h. The crude residue was purified by column chromatography on silica gel (hexane/EtOAc = 5:1 to 1:1). R_f_ = 0.20 (hexane/EtOAc = 4:1). m.p: 256–258 °C. ^1^H NMR (600 MHz, DMSO) *δ* 8.03 (d, *J* = 8.9 Hz, 2H), 7.62 (d, *J* = 9.4 Hz, 1H), 7.37 − 7.31 (m, 1H), 7.11 (d, *J* = 8.9 Hz, 2H), 6.88 (s, 1H), 3.85 (s, 3H); ^13^C NMR (150 MHz, DMSO) *δ* 175.01, 165.48, 162.88, 162.34, 128.69, 123.01, 115.14, 106.97, 102.55, 102.46, 102.43, 102.38, 102.29, 102.26, 102.21, 56.08. HRMS *m*/*z* calculated for C_16_H_10_F_2_O_3_ [M + H]^+^: 289.0671; found: 289.0657. >95% purity (as determined by RP-HPLC, method B, *t*_R_ = 6.73 min).

##### Ethyl 4–(6,7-dimethoxy-4-oxo-4H-chromen-2-yl)benzoate (15a)

Compound **15a** was prepared in 43% yield as a white powder, following the same procedure as described in the general procedure B with *(E)-Ethyl 4–(3-(2-hydroxy-4,5-dimethoxyphenyl)-3-oxoprop-1-en-1-yl)benzoate* (**14a**) (241 mg, 0.70 mmol), iodine (18 mg, 0.1 eq) in DMSO (5 mL), stirring for 11 h. The crude residue was purified by column chromatography on silica gel (hexane/EtOAc = 5:1 to 1:1 and then CH_2_Cl_2_/MeOH = 50:1 to 30:1). R_f_ = 0.34 (hexane/EtOAc = 1:1). ^1^H NMR (600 MHz, CDCl_3_) *δ* 8.15 (d, *J* = 8.7 Hz, 2H), 7.94 (d, *J* = 8.6 Hz, 2H), 7.52 (s, 1H), 6.99 (s, 1H), 6.82 (s, 1H), 4.42 (q, *J* = 7.1 Hz, 2H), 4.03 (s, 3H), 3.97 (s, 3H), 1.44 (t, *J* = 6.7 Hz, 3H); ^13^C NMR (150 MHz, CDCl_3_) *δ* 177.44, 165.74, 161.44, 154.72, 152.33, 147.88, 135.85, 132.78, 130.12, 125.96, 117.40, 108.21, 104.36, 99.80, 61.45, 56.55, 56.39, 14.31. HRMS *m*/*z* calculated for C_20_H_18_O_6_ [M + H]^+^: 355.1176; found: 355.1184.

##### Ethyl 3–(6,7-dimethoxy-4-oxo-4H-chromen-2-yl)benzoate (15b)

Compound **15b** was prepared in quantitative yield as a white powder, following the same procedure as described in the general procedure B with *(E)-Ethyl 3–(3-(2-hydroxy-4,5-dimethoxyphenyl)-3-oxoprop-1-en-1-yl)benzoate* (**14b**) (70 mg, 0.19 mmol), iodine (5 mg, 0.1 eq) in DMSO (8 mL), stirring for 12 h. The crude residue was purified by column chromatography on silica gel (hexane/EtOAc = 2:1 to 1:1 and then CH_2_Cl_2_/MeOH = 10:1). R_f_ = 0.19 (hexnae/EtOAc = 1:1). ^1^H NMR (600 MHz, CDCl_3_) *δ* 8.61 (s, 1H), 8.20 (d, *J* = 7.8 Hz, 1H), 8.08 (d, *J* = 8.0 Hz, 1H), 7.61 (t, *J* = 7.9 Hz, 1H), 7.58 (s, 1H), 7.06 (s, 1H), 6.86 (s, 1H), 4.46 (q, *J* = 7.1 Hz, 2H), 4.04 (s, 3H), 4.01 (s, 3H), 1.45 (t, *J* = 7.1 Hz, 3H). HRMS *m*/*z* calculated for C_20_H_18_O_6_ [M + H]^+^: 355.1176; found: 355.1184.

##### Ethyl 4–(5,7-dimethoxy-4-oxo-4H-chromen-2-yl)benzoate (15c)

Compound **15c** was prepared in 87% yield as a white powder, following the same procedure as described in the general procedure B with *(E)-Ethyl 4–(3-(2-hydroxy-4,6-dimethoxyphenyl)-3-oxoprop-1-en-1-yl)benzoate* (**14c**) (116 mg, 0.34 mmol), iodine (8.7 mg, 0.1 eq) in DMSO (8 mL), stirring for 11 h. The crude residue was purified by column chromatography on silica gel (CH_2_Cl_2_/MeOH = 100:1 to 10:1). R_f_ = 0.28 (hexane/EtOAc = 1:1). ^1^H NMR (600 MHz, CDCl_3_) *δ* 8.2 (d, *J* = 8.3 Hz, 2H), 7.94 (d, *J* = 8.3 Hz, 2H), 6.74 (s, 1H), 6.60 (d, *J* = 2.1 Hz, 1H), 6.40 (d, *J* = 2.1 Hz, 1H), 4.43 (q, *J* = 7.1 Hz, 2H), 3.97 (s, 3H), 3.93 (s, 3H), 1.43 (t, *J* = 7.1 Hz, 3H). HRMS *m*/*z* calculated for C_20_H_18_O_6_ [M + H]^+^: 355.1176; found: 355.1169.

##### Ethyl 3–(5,7-dimethoxy-4-oxo-4H-chromen-2-yl)benzoate (15d)

Compound **15d** was prepared in 81% yield as a white powder, following the same procedure as described in the general procedure B with *(E)-Ethyl 3–(3-(2-hydroxy-4,6-dimethoxyphenyl)-3-oxoprop-1-en-1-yl)benzoate* (**14d**) (271 mg, 0.76 mmol), iodine (19.3 mg, 0.1 eq) in DMSO (10 mL), stirring for 16 h. The crude residue was purified by column chromatography on silica gel (CH_2_Cl_2_/MeOH = 200:1 to 60:1). R_f_ = 0.37 (CH_2_Cl_2_/MeOH = 20:1). ^1^H NMR (600 MHz, CDCl_3_) *δ* 8.56 (s, 1H), 8.19 (d, *J* = 7.8 Hz, 1H), 8.05 (d, *J* = 7.9 Hz, 1H), 7.59 (t, *J* = 7.8 Hz, 1H), 6.75 (s, 1H), 6.63 (d, *J* = 2.2 Hz, 1H), 6.40 (d, *J* = 2.1 Hz, 1H), 4.45 (q, *J* = 7.1 Hz, 2H), 3.99 (s, 3H), 3.93 (s, 3H), 1.45 (t, *J* = 7.3 Hz, 3H); ^13^C NMR (150 MHz, CDCl_3_) *δ* 177.36, 165.75, 164.19, 160.87, 159.83, 159.49, 131.94, 131.86, 131.35, 129.87, 129.08, 126.98, 109.49, 109.23, 96.34, 92.88, 61.47, 56.42, 55.84, 14.35. HRMS *m*/*z* calculated for C_20_H_18_O_6_ [M + H]^+^: 355.1176; found: 355.1174.

#### *General* Procedure C *for compounds 10b–10k, 16a–16d, 20a–20s*

To a stirred solution of cyclized compound (**9b–9k, 15a–15d** and **19a–19t**) in dichloromethane (CH_2_Cl_2_) was added boron tribromide (5–12 eq) at 0 °C. The reaction mixture was stirred under argon at 50 °C until complete conversion monitored by TLC analysis (typically 14–20 h), quenched with ice water, and concentrated CH_2_Cl_2._ The crude residue was extracted with EtOAc. The organic layer was washed with saturated NaHCO_3_ solution, dried over MgSO_4_, filtered, and concentrated under reduced pressure. The crude was purified by column chromatography on a silica gel (CH_2_Cl_2_/MeOH or hexane/EtOAc) or washed with organic solvents such as ether or hexane to furnish compounds **10b–10k, 16a–16d** and **20a–20s**.

##### 7-Fluoro-2–(4-hydroxyphenyl)-4H-chromen-4-one (10b)

Compound **10b** was prepared in quantitative yield as a brown powder, following the same procedure as described in the general procedure C with *7-Fluoro-2–(4-methoxyphenyl)-4H-chromen-4-one* (**9b**) (76 mg, 0.28 mmol) and boron tribromide (3.36 mL, 12 eq) in dichloromethane (30 mL), stirring for 18 h at 50 °C. The crude residue was purified by column chromatography on silica gel (CH_2_Cl_2_/MeOH = 50:1 to 10:1). R_f_ = 0.38 (CH_2_Cl_2/_MeOH = 10:1). m.p: 310–312 °C. ^1^H NMR (600 MHz, DMSO) *δ* 10.32 (s, 1H), 8.07 (dd, *J* = 6.5 and 8.8 Hz, 1H), 7.97 − 7.92 (m, 2H), 7.69 (dd, *J* = 2.4 and 9.6 Hz, 1H), 7.37 − 7.32 (m, 1H), 6.95 − 6.90 (m, 2H), 6.86 (s, 1H); ^13^C NMR (150 MHz, DMSO) *δ* 176.54, 166.18, 164.51, 163.87, 161.56, 157.15, 157.06, 128.86, 128.03, 127.96, 121.75, 120.94, 116.43, 114.36, 114.20, 105.79, 105.62, 105.24. HRMS *m*/*z* calculated for C_15_H_9_FO_3_ [M + H]^+^: 257.0609; found: 257.0616. >95% purity (as determined by RP-HPLC, method D, *t*_R_ = 7.67 min).

##### 6-Fluoro-2–(4-hydroxyphenyl)-4H-chromen-4-one (10d)

Compound **10d** was prepared in 97% yield as a yellow powder, following the same procedure as described in the general procedure C with *6-Fluoro-2–(4-methoxyphenyl)-4H-chromen-4-one* (**9d**) (100 mg, 0.37 mmol) and boron tribromide (4.44 mL, 12 eq) in dichloromethane (15 mL), stirring for 11 h. The crude residue was purified by column chromatography on silica gel (CH_2_Cl_2_/MeOH = 10:1). R_f_ = 0.21 (hexane/EtOAc = 4:1). m.p: 292–294 °C. ^1^H NMR (600 MHz, MeOD) *δ* 7.96 − 7.89 (m, 2H), 7.81 − 7.74 (m, 2H), 7.63 − 7.55 (m, 1H), 6.98 − 6.93 (m, 2H), 6.81 (s, 1H); ^13^C NMR (150 MHz, DMSO) *δ* 176.79, 164.00, 160.19, 158.62, 152.49, 128.98, 124.97, 122.56, 122.39, 121.65, 121.59, 116.47, 109.94, 109.79, 104.59. HRMS *m*/*z* calculated for C_15_H_9_FO_3_ [M **−** H]**^−^**: 257.0609; found: 257.0599. >95% purity (as determined by RP-HPLC, method D, *t*_R_ = 6.26 min).

##### 5-Fluoro-2–(4-hydroxyphenyl)-4H-chromen-4-one (10f)

Compound **10f** was prepared in 96% yield as a white powder, following the same procedure as described in the general procedure C with *5-Fluoro-2–(4-methoxyphenyl)-4H-chromen-4-one* (**9f**) (100 mg, 0.37 mmol) and boron tribromide (4.44 mL, 12 eq) in dichloromethane (30 mL), stirring for 18 h at 50 °C. The crude residue was purified by column chromatography on silica gel (CH_2_Cl_2_/MeOH = 50:1 to 10:1). R_f_ = 0.38 (CH_2_Cl_2/_MeOH = 10:1). m.p: 272–274 °C. ^1^H NMR (600 MHz, DMSO) *δ* 10.31 (brs, 1H), 7.93 (d, *J* = 8.8 Hz, 2H), 7.81 − 7.73 (m, 1H), 7.57 (d, *J* = 8.5 Hz, 1H), 7.21 (dd, *J* = 8.3 and 10.7 Hz, 1H), 6.92 (d, *J* = 8.8 Hz, 2H), 6.79 (s, 1H); ^13^C NMR (150 MHz, DMSO) *δ* 175.81, 162.53, 161.56, 160.89, 159.16, 157.13, 134.81, 134.74, 128.84, 121.47, 116.44, 115.03, 115.01, 113.99, 113.92, 112.54, 112.41, 106.28. HRMS *m*/*z* calculated for C_15_H_9_FO_3_ [M + H]^+^: 257.0609; found: 257.0618. >95% purity (as determined by RP-HPLC, method D, *t*_R_ = 6.12 min).

##### 6,7-Difluoro-2–(4-hydroxyphenyl)-4H-chromen-4-one (10h)

Compound **10h** was prepared in quantitative yield as a white powder, following the same procedure as described in the general procedure C with *6,7-Difluoro-2–(4-methoxyphenyl)-4H-chromen-4-one* (**9h**) (123 mg, 0.43 mmol) and boron tribromide (5.12 mL, 12 eq) in dichloromethane (30 mL), stirring for 17 h at 50 °C. The crude residue was purified by column chromatography on silica gel (CH_2_Cl_2_/MeOH = 50:1 to 3:1). R_f_ = 0.60 (CH_2_Cl_2/_MeOH = 10:1). m.p: 302–304 °C. ^1^H NMR (600 MHz, DMSO) *δ* 10.34 (brs, 1H), 8.03 (dd, *J* = 6.5 and 10.8 Hz, 1H), 7.96 − 7.90 (m, 2H), 6.94 − 6.91 (m, 2H), 6.89 (s, 1H); ^13^C NMR (150 MHz, DMSO) *δ* 175.93, 164.19, 161.69, 152.52, 128.92, 121.59, 121.09, 116.45, 112.60, 112.47, 108.76, 108.61, 104.72. HRMS *m*/*z* calculated for C_15_H_8_F_2_O_3_ [M + H]^+^: 275.0515; found: 275.0527. >95% purity (as determined by RP-HPLC, method D, *t*_R_ = 9.11 min).

##### 3–(6,7-Difluoro-4-oxo-4H-chromen-2-yl)benzoic acid (10i)

Compound **10i** was prepared in 31% yield as a white powder, following the same procedure as described in the general procedure C with *ethyl 3–(6,7-Difluoro-4-oxo-4H-chromen-2-yl)benzoate* (**9i**) (74 mg, 0.23 mmol) and boron tribromide (2.70 mL, 12 eq) in dichloromethane (15 mL), stirring for 18 h at 50 °C. The reaction mixture was filtered through a filter paper and concentrated under reduced pressure. The crude residue was purified by column chromatography on silica gel (CH_2_Cl_2_/MeOH = 50:1 to 3:1). R_f_ = 0.51 (CH_2_Cl_2/_MeOH = 10:1). m.p: 272–274 °C. ^1^H NMR (600 MHz, MeOD) *δ* 8.66 (s, 1H), 8.29 − 8.23 (m, 2H), 7.99 (t, *J* = 8.8 Hz, 1H), 7.85 (dd, *J* = 6.4 and 10.4 Hz, 1H), 7.71 (t, *J* = 7.9 Hz, 1H), 7.0 (s, 1H). HRMS *m*/*z* calculated for C_16_H_8_F_2_O_4_ [M **−** H]**^−^**: 301.0318; found: 301.0298. >95% purity (as determined by RP-HPLC, method D, *t*_R_ = 8.00 min).

##### 5,7-Difluoro-2–(4-hydroxyphenyl)-4H-chromen-4-one (10k)

Compound **10k** was prepared in 94% yield as a white powder, following the same procedure as described in the general procedure C with *5,7-Difluoro-2–(4-methoxyphenyl)-4H-chromen-4-one* (**9k**) (100 mg, 0.35 mmol) and boron tribromide (4.16 mL, 12 eq) in dichloromethane (30 mL), stirring for 14 h at 50 °C. The crude residue was purified by column chromatography on silica gel (CH_2_Cl_2_/MeOH = 50:1 to 10:1). R_f_ = 0.60 (CH_2_Cl_2/_MeOH = 10:1). m.p: 228–230 °C. ^1^H NMR (600 MHz, DMSO) *δ* 10.34 (brs, 1H), 7.92 (d, *J* = 8.5 Hz, 2H), 7.58 (d, *J* = 9.0 Hz, 1H), 7.35 − 7.28 (m, 1H), 6.91 (d, *J* = 8.8 Hz, 2H), 6.78 (s, 1H); ^13^C NMR (150 MHz, DMSO) *δ* 175.07, 162.66, 161.77, 157.95, 128.84, 121.08, 116.47, 111.49, 106.15, 102.38, 49.07F10. HRMS *m*/*z* calculated for C_15_H_8_F_2_O_3_ [M + H]^+^: 275.0515; found: 275.0524. >95% purity (as determined by RP-HPLC, method E, *t*_R_ = 8.99 min).

##### 4–(6,7-Dihydroxy-4-oxo-4H-chromen-2-yl)benzoic acid (16a)

Compound **16a** was prepared in 87% yield as a yellow powder, following the same procedure as described in the general procedure C with *Ethyl 4–(6,7-dimethoxy-4-oxo-4H-chromen-2-yl)benzoate* (**15a**) (82 mg, 0.23 mmol) and boron tribromide (2.78 mL, 12 eq) in dichloromethane (30 mL), stirring for 18 h at 50 °C. The reaction mixture was filtered through a filter paper and concentrated under reduced pressure. The crude residue was washed with ether, hexane and methanol. R_f_ = 0.19 (CH_2_Cl_2/_MeOH = 10:1). m.p: 500–502 °C. ^1^H NMR (600 MHz, MeOD) *δ* 8.18 (d, *J* = 8.5 Hz, 2H), 8.11 (d, *J* = 8.5 Hz, 2H), 7.42 (s, 1H), 7.08 (s, 1H), 6.90 (s, 1H). HRMS *m*/*z* calculated for C_16_H_10_O_6_ [M **−** H]**^−^**: 297.0404; found: 297.0418. >95% purity (as determined by RP-HPLC, method H, *t*_R_ = 14.84 min).

##### 3–(6,7-Dihydroxy-4-oxo-4H-chromen-2-yl)benzoic acid (16b)

Compound **16b** was prepared in 87% yield as a yellow powder, following the same procedure as described in the general procedure C with *Ethyl 3–(6,7-dimethoxy-4-oxo-4H-chromen-2-yl)benzoate* (**15b**) (50.4 mg, 0.14 mmol) and boron tribromide (1.71 mL, 12 eq) in dichloromethane (15 mL), stirring for 17 h at 50 °C. The reaction mixture was filtered through a filter paper and concentrated under reduced pressure. The crude residue was washed with ether, hexane, and methanol. R_f_ = 0.24 (CH_2_Cl_2/_MeOH = 10:1). m.p: 416–418 °C. ^1^H NMR (600 MHz, MeOD) *δ* 8.64 (t, *J* = 1.6 Hz, 1H), 8.27 − 8.21 (m, 2H), 7.71 (t, *J* = 7.9 Hz, 1H), 7.45 (s, 1H), 7.12 (s, 1H), 6.92 (s, 1H); ^13^C NMR (150 MHz, MeOD) *δ* 178.28, 167.30, 162.78, 153.77, 152.44, 145.33, 132.24, 132.03, 131.75, 130.16, 129.19, 126.93, 115.79, 106.95, 105.41, 102.53. HRMS *m*/*z* calculated for C_16_H_10_O_6_ [M **−** H]**^−^**: 297.0404; found: 297.0399. >95% purity (as determined by RP-HPLC, method G, *t*_R_ = 6.70 min).

##### 4–(5,7-Dihydroxy-4-oxo-4H-chromen-2-yl)benzoic acid (16c)

Compound **16c** was prepared in 50% yield as a yellow powder, following the same procedure as described in the general procedure C with *Ethyl 4–(5,7-dimethoxy-4-oxo-4H-chromen-2-yl)benzoate* (**15c**) (30 mg, 0.08 mmol) and boron tribromide (1.02 mL, 12 eq) in dichloromethane (15 mL), stirring for 18 h at 50 °C. The reaction mixture was filtered through a filter paper and concentrated under reduced pressure. The crude residue was washed with ether, hexane and methanol. R_f_ = 0.26 (CH_2_Cl_2/_MeOH = 10:1). m.p: 337–339 °C. ^1^H NMR (600 MHz, MeOD) *δ* 8.08 (d, *J* = 7.9 Hz, 2H), 8.00 (d, *J* = 7.5 Hz, 2H), 6.75 (s, 1H), 6.42 (s, 1H), 6.15 (s, 1H). HRMS *m*/*z* calculated for C_16_H_10_O_6_ [M **−** H]**^−^**: 297.0404; found: 297.0419. >95% purity (as determined by RP-HPLC, method E, *t*_R_ = 7.99 min).

##### 3–(5,7-Dihydroxy-4-oxo-4H-chromen-2-yl)benzoic acid (16d)

Compound **16d** was prepared in 70% yield as a yellow powder, following the same procedure as described in the general procedure C with *Ethyl 3–(5,7-dimethoxy-4-oxo-4H-chromen-2-yl)benzoate* (**15d**) (104 mg, 0.29 mmol) and boron tribromide (3.54 mL, 12 eq) in dichloromethane (15 mL), stirring for 18 h at 50 °C. The reaction mixture was filtered through a filter paper and concentrated under reduced pressure. The crude residue was purified by column chromatography on silica gel (CH_2_Cl_2_/MeOH = 10:1 to 3:1). R_f_ = 0.40 (CH_2_Cl_2/_MeOH = 10:1). m.p: 450–452 °C. ^1^H NMR (600 MHz, DMSO) *δ* 12.79 (s, 1H), 8.50 (s, 1H), 8.31 (d, *J* = 5.4 Hz, 1H), 8.15 (d, *J* = 6.0 Hz, 1H), 7.71 (s, 1H), 7.03 (s, 1H), 6.54 (s, 1H), 6.24 (s, 1H); ^13^C NMR (150 MHz, DMSO) *δ* 182.28, 167.22, 165.07, 162.78, 161.96, 157.95, 132.92, 132.57, 131.70, 131.06, 130.06, 127.26, 106.37, 104.48, 99.58, 94.62. HRMS *m*/*z* calculated for C_16_H_10_O_6_ [M **−** H]**^−^**: 297.0404; found: 297.0398. >95% purity (as determined by RP-HPLC, method E, *t*_R_ = 7.18 min).

##### 2–(3,4-Dihydroxyphenyl)-3,6,7-trihydroxy-4H-chromen-4-one (20a)

Compound **20a** was prepared in 89% yield as a brown powder, following the same procedure as described in the general procedure C with *2–(3,4-dimethoxyphenyl)-3-hydroxy-6,7-dimethoxy-4H-chromen-4-one* (**19a**) (120 mg, 0.34 mmol) and boron tribromide (5.04 mL, 15 eq) in dichloromethane (20 mL), stirring for 15 h at 50 °C. The reaction mixture was filtered through a filter paper and concentrated under reduced pressure. The crude residue was washed with ether, hexane and methanol. R_f_ = 0.13 (CH_2_Cl_2/_MeOH = 10:1). ^1^H NMR (600 MHz, DMSO) δ 10.41 (s, 1H), 9.71 (s, 1H), 9.46 (s, 1H), 9.24 (s, 1H), 8.85 (s, 1H), 7.66 (d, *J* = 1.3 Hz, 1H), 7.52 (d, *J* = 8.4 Hz, 1H), 7.30 (s, 1H), 6.92 (s, 1H), 6.88 (d, *J* = 8.4 Hz, 1H). HRMS *m*/*z* calculated for C_15_H_10_O_7_ [M + H]^+^: 301.0354; found: 301.0351.

##### 3,6,7-Trihydroxy-2-phenyl-4H-chromen-4-one (20b)

Compound **20b** was prepared in 41% yield as a brown powder, following the same procedure as described in the general procedure C with *3-hydroxy-6,7-dimethoxy-2-phenyl-4H-chromen-4-one* (**19b**) (40 mg, 0.11 mmol) and boron tribromide (1.13 mL, 10 eq) in dichloromethane (15 mL), stirring for 6 h at 50 °C. The crude residue was purified by column chromatography on silica gel (CH_2_Cl_2_/MeOH = 50:1 to 10:1). R_f_ = 0.39 (CH_2_Cl_2/_MeOH = 10:1). ^1^H NMR (600 MHz, DMSO) δ 8.15 (d, *J* = 7.5 Hz, 2H), 7.54 (t, *J* = 7.7 Hz, 2H), 7.46 (t, *J* = 7.3 Hz, 1H), 7.32 (s, 1H), 6.97 (s, 1H); ^13^C NMR (150 MHz, DMSO) δ 171.83, 152.64, 150.27, 144.42, 143.70, 138.05, 131.71, 129.34, 128.46, 127.26, 114.04, 106.91, 102.65. HRMS *m*/*z* calculated for C_15_H_10_O_5_ [M + H]^+^: 269.0455; found: 269.0464. >95% purity (as determined by RP-HPLC, method F, *t*_R_ = 13.617 min).

##### 3,6,7-Trihydroxy-2-(p-tolyl)-4H-chromen-4-one (20c)

Compound **20c** was prepared in 29% yield as a yellow powder, following the same procedure as described in the general procedure C with *3-hydroxy-6,7-dimethoxy-2-(p-tolyl)-4H-chromen-4-one* (**19c**) (90 mg, 0.29 mmol) and boron tribromide (3.46 mL, 12 eq) in dichloromethane (20 mL), stirring for 6 h at 50 °C. The crude residue was purified by column chromatography on silica gel (CH_2_Cl_2_/MeOH = 10:1 to 4:1). R_f_ = 0.23 (CH_2_Cl_2/_MeOH = 10:1). ^1^H NMR (600 MHz, MeOD) δ 8.12 (d, *J* = 8.3 Hz, 2H), 7.40 (s, 1H), 7.33 (d, *J* = 8.1 Hz, 2H), 6.98 (s, 1H), 2.41 (s, 3H). ^13^C NMR (150 MHz, DMSO) δ 171.75, 152.60, 150.20, 144.39, 143.94, 139.11, 137.73, 129.07, 128.94, 127.18, 114.00, 106.90, 102.64, 21.01. HRMS *m*/*z* calculated for C_16_H_12_O_5_ [M + H]^+^: 285.0758; found: 285.0766. >95% purity (as determined by RP-HPLC, method E, *t*_R_ = 12.49 min).

##### 3,6,7-Trihydroxy-2-(m-tolyl)-4H-chromen-4-one (20d)

Compound **20d** was prepared in 20% yield as a brown powder, following the same procedure as described in the general procedure C with *3-hydroxy-6,7-dimethoxy-2-(m-tolyl)-4H-chromen-4-one* (**19d**) (70 mg, 0.25 mmol) and boron tribromide (3.69 mL, 15 eq) in dichloromethane (20 mL), stirring for 13 h at 50 °C. The crude residue was purified by column chromatography on silica gel (CH_2_Cl_2_/MeOH = 50:1 to 10:1). R_f_ = 0.50 (CH_2_Cl_2_/MeOH = 10:1). ^1^H NMR (600 MHz, MeOD) δ 8.06 − 7.97 (m, 2H), 7.38 (dd, *J* = 15.1, 7.4 Hz, 2H), 7.26 (d, *J* = 7.3 Hz, 1H), 6.98 (s, 3H), 2.42 (s, 3H); ^13^C NMR (150 MHz, MeOD) δ 174.31, 154.43, 152.87, 146.49, 145.91, 139.21, 133.01, 131.37, 129.36, 129.34, 129.03, 125.93, 115.45, 107.76, 103.46, 21.63. HRMS *m*/*z* calculated for C_16_H_12_O_5_ [M – H]^–^: 283.0612; found: 283.0606. >95% purity (as determined by RP-HPLC, method D, *t*_R_ = 11.64 min).

##### 2–(3,4-Dimethylphenyl)-3,6,7-trihydroxy-4H-chromen-4-one (20e)

Compound **20e** was prepared in 85% yield as a brown powder, following the same procedure as described in the general procedure C with *2–(3,4-dimethylphenyl)-3-hydroxy-6,7-dimethoxy-4H-chromen-4-one* (**19e**) (40 mg, 0.12 mmol) and boron tribromide (1.22 mL, 10 eq) in dichloromethane (20 mL), stirring for 16 h at 50 °C. The crude residue was purified by column chromatography on silica gel (CH_2_Cl_2_/MeOH = 30:1 to 4:1). R_f_ = 0.45 (CH_2_Cl_2/_MeOH = 10:1). ^1^H NMR (600 MHz, DMSO) δ 7.94 (s, 1H), 7.89 (d, *J* = 8.0 Hz, 1H), 7.32 (s, 1H), 7.30 (d, *J* = 8.0 Hz, 1H), 6.98 (s, 1H), 2.31 (d, *J* = 7.0 Hz, 3H), 2.29 (s, 3H); ^13^C NMR (150 MHz, DMSO) δ 171.72, 152.51, 150.20, 144.35, 144.12, 137.99, 137.70, 136.27, 129.58, 129.27, 128.03, 124.92, 114.03, 106.93, 102.67, 40.06, 39.94, 39.80, 39.66, 39.52, 39.38, 39.24, 39.10, 19.65, 19.39. HRMS *m*/*z* calculated for C_17_H_14_O_5_ [M + H]^+^: 299.0914; found: 299.0922. >95% purity (as determined by RP-HPLC, method D, *t*_R_ = 10.22 min).

##### 2–(4-Fluorophenyl)-3,6,7-trihydroxy-4H-chromen-4-one (20f)

Compound **20f** was prepared in 66% yield as a brown powder, following the same procedure as described in the general procedure C with *2–(4-fluorophenyl)-3-hydroxy-6,7-dimethoxy-4H-chromen-4-one* (**19f**) (40 mg, 0.13 mmol) and boron tribromide (1.26 mL, 10 eq) in dichloromethane (15 mL), stirring for 16 h at 50 °C. The crude residue was purified by column chromatography on silica gel (CH_2_Cl_2_/MeOH = 50:1 to 10:1). R_f_ = 0.37 (CH_2_Cl_2/_MeOH = 10:1). ^1^H NMR (600 MHz, DMSO) δ 8.20 − 8.15 (m, 2H), 7.40 − 7.32 (m, 2H), 7.31 (s, 1H), 6.99 (s, 1H); ^13^C NMR (150 MHz, DMSO) δ 172.19, 168.24, 163.61, 161.96, 152.79, 150.65, 148.27, 144.63, 143.59, 137.96, 130.16, 130.11, 128.43, 116.04, 115.89, 114.43, 107.24, 103.01, 79.36. HRMS *m*/*z* calculated for C_15_H_9_FO_5_ [M – H]^–^: 287.0361; found: 287.0375. >95% purity (as determined by RP-HPLC, method E, *t*_R_ = 9.18 min).

##### 2–(3-Fluorophenyl)-3,6,7-trihydroxy-4H-chromen-4-one (20g)

Compound **20 g** was prepared in 92% yield as a brown powder, following the same procedure as described in the general procedure C with *2–(3-fluorophenyl)-3-hydroxy-6,7-dimethoxy-4H-chromen-4-one* (**19 g**) (110 mg, 0.35 mmol) and boron tribromide (5.2 mL, 15 eq) in dichloromethane (20 mL), stirring for 16 h at 50 °C. The crude residue was purified by column chromatography on silica gel (CH_2_Cl_2_/MeOH = 50:1 to 10:1). R_f_ = 0.35 (CH_2_Cl_2/_MeOH = 10:1). ^1^H NMR (600 MHz, DMSO) δ 10.53 (s, 1H), 9.81 (s, 1H), 9.47 (s, 1H), 8.01 (d, *J* = 8.0 Hz, 1H), 7.96 (d, *J* = 10.8 Hz, 1H), 7.58 (dd, *J* = 14.5, 7.9 Hz, 1H), 7.36 − 7.25 (m, 2H), 7.02 (s, 1H); ^13^C NMR (150 MHz, DMSO) δ 171.87, 162.87, 161.26, 152.85, 150.28, 144.55, 142.14, 138.64, 133.99, 130.61, 130.56, 123.25, 116.16, 116.02, 114.07, 113.89, 113.73, 106.90, 102.77. HRMS *m*/*z* calculated for C_15_H_9_FO_5_ [M – H]^–^: 287.0361; found: 287.0367. >95% purity (as determined by RP-HPLC, method E, *t*_R_ = 10.17 min).

##### 2–(3,4-Difluorophenyl)-3,6,7-trihydroxy-4H-chromen-4-one (20h)

Compound **20h** was prepared in 40% yield as a brown powder, following the same procedure as described in the general procedure C with *2–(3,4-difluorophenyl)-3-hydroxy-6,7-dimethoxy-4H-chromen-4-one* (**19h**) (40 mg, 0.12 mmol) and boron tribromide (1.20 mL, 10 eq) in dichloromethane (15 mL), stirring for 16 h at 50 °C. The crude residue was purified by column chromatography on silica gel (CH_2_Cl_2_/MeOH = 50:1 to 10:1). R_f_ = 0.42 (CH_2_Cl_2/_MeOH = 10:1). ^1^H NMR (600 MHz, DMSO) δ 8.18 (s, 1H), 8.04 (s, 1H), 7.61 (d, *J* = 7.5 Hz, 1H), 7.31 (s, 1H), 7.07 − 6.96 (m, 1H); ^13^C NMR (150 MHz, DMSO) 171.81, 152.81, 150.2, 144.54, 141.47, 138.40, 129.29, 124.53, 117.87, 117.76, 116.32, 116.19, 114.05, 106.89, 102.78. HRMS *m*/*z* calculated for C_15_H_8_F_2_O_5_ [M – H]^–^: 305.0267; found: 305.0281. >95% purity (as determined by RP-HPLC, method E, *t*_R_ = 12.85 min,).

##### 3,6,7-Trihydroxy-2–(4-(trifluoromethyl)phenyl)-4H-chromen-4-one (20i)

Compound **20i** was prepared in 35% yield as a brown powder, following the same procedure as described in the general procedure C with *3-hydroxy-6,7-dimethoxy-2–(4-(trifluoromethyl)phenyl)-4H-chromen-4-one* (**19i**) (27 mg, 0.07 mmol) and boron tribromide (1.10 mL, 15 eq) in dichloromethane (20 mL), stirring for 13 h at 50 °C. The crude residue was purified by column chromatography on silica gel (CH_2_Cl_2_/MeOH = 50:1 to 4:1). R_f_ = 0.26 (CH_2_Cl_2/_MeOH = 10:1). ^1^H NMR (600 MHz, DMSO) δ 10.57 (s, 1H), 9.83 (s, 1H), 9.62 (s, 1H), 8.37 (d, *J* = 8.3 Hz, 2H), 7.90 (d, *J* = 8.4 Hz, 2H), 7.33 (s, 1H), 7.00 (s, 1H). ^13^C NMR (150 MHz, DMSO) δ 171.91, 152.97, 150.37, 144.62, 144.60, 141.84, 139.18, 135.71, 129.01, 128.81, 127.79, 125.39, 125.37, 114.10, 106.91, 102.67. HRMS *m*/*z* calculated for C_16_H_9_F_3_O_5_ [M – H]^–^: 337.0329; found: 337.0343. >95% purity (as determined by RP-HPLC, method D, *t*_R_ = 13.83 min).

##### 3,6,7-Trihydroxy-2–(3-(trifluoromethyl)phenyl)-4H-chromen-4-one (20j)

Compound **20j** was prepared in 57% yield as a brown powder, following the same procedure as described in the general procedure C with *3-hydroxy-6,7-dimethoxy-2–(3-(trifluoromethyl)phenyl)-4H-chromen-4-one* (**19j**) (40 mg, 0.11 mmol) and boron tribromide (1.64 mL, 15 eq) in dichloromethane (20 mL), stirring for 15 h at 50 °C. The reaction mixture was filtered through a filter paper and concentrated under reduced pressure. The crude residue was purified by column chromatography on silica gel (CH_2_Cl_2_/MeOH = 50:1 to 4:1). R_f_ = 0.21 (CH_2_Cl_2/_MeOH = 10:1). ^1^H NMR (600 MHz, MeOD) δ 8.57 (s, 1H), 8.47 (s, 1H), 7.76 − 7.69 (m, 2H), 7.42 (s, 1H), 7.01 (s, 1H). ^13^C NMR (150 MHz, DMSO) δ 171.85, 152.89, 150.31, 144.57, 141.82, 138.81, 132.78, 130.71, 129.78, 125.65, 125.02, 123.66, 123.22, 114.11, 106,90, 102.77. HRMS *m*/*z* calculated for C_16_H_9_F_3_O_5_ [M – H]^–^: 337.0329; found: 337.0332. >95% purity (as determined by RP-HPLC, method A, *t*_R_ = 6.91 min).

##### 2–(4-Chlorophenyl)-3,6,7-trihydroxy-4H-chromen-4-one (20k)

Compound **20k** was prepared in 55% yield as a brown powder, following the same procedure as described in the general procedure C with *2–(4-chlorophenyl)-3-hydroxy-6,7-dimethoxy-4H-chromen-4-one* (**19k**) (40 mg, 0.12 mmol) and boron tribromide (1.20 mL, 10 eq) in dichloromethane (15 mL), stirring for 16 h at 50 °C. The crude residue was purified by column chromatography on silica gel (CH_2_Cl_2_/MeOH = 50:1 to 10:1). R_f_ = 0.48 (CH_2_Cl_2/_MeOH = 10:1). ^1^H NMR (600 MHz, DMSO) δ 8.18 (d, *J* = 8.7 Hz, 2H), 7.60 (d, *J* = 8.7 Hz, 2H), 7.31 (s, 1H), 6.97 (s, 1H); ^13^C NMR (150 MHz, DMSO) δ 171.79, 152.85, 150.24, 144.53, 142.50, 138.35, 133.82, 130.62, 128.93, 128.59, 114.01, 106.87, 102.63. HRMS *m*/*z* calculated for C_15_H_9_ClO_5_ [M – H]^–^: 303.0066; found: 303.0078. >95% purity (as determined by RP-HPLC, method D, *t*_R_ = 9.11 min).

##### 2–(3-Chlorophenyl)-3,6,7-trihydroxy-4H-chromen-4-one (20l)

Compound **20 l** was prepared in 66% yield as a brown powder, following the same procedure as described in the general procedure C with *2–(3-chlorophenyl)-3-hydroxy-6,7-dimethoxy-4H-chromen-4-one* (**19 l**) (65 mg, 0.20 mmol) and boron tribromide (2.92 mL, 15 eq) in dichloromethane (20 mL), stirring for 16 h at 50 °C. The crude residue was purified by column chromatography on silica gel (CH_2_Cl_2_/MeOH = 50:1 to 10:1). R_f_ = 0.39 (CH_2_Cl_2/_MeOH = 10:1). ^1^H NMR (600 MHz, DMSO) δ 8.21 (s, 1H), 8.11 (d, *J* = 7.5 Hz, 1H), 7.57 (t, *J* = 7.7 Hz, 1H), 7.53 (d, *J* = 7.3 Hz, 1H), 7.31 (s, 1H), 7.01 (s, 1H); ^13^C NMR (150 MHz, DMSO) δ 171.83, 152.88, 150.31, 144.56, 141.93, 138.67, 133.77, 133.28, 130.44, 129.03, 126.75, 125.62, 114.06, 106.86, 102.74. HRMS *m*/*z* calculated for C_15_H_9_ClO_5_ [M – H]^–^: 303.0066; found: 303.0077. >95% purity (as determined by RP-HPLC, method D, *t*_R_ = 8.87).

##### 2-([1,1'-Biphenyl]-4-yl)-3,6,7-trihydroxy-4H-chromen-4-one (20m)

Compound **20 m** was prepared in 87% yield as a brown powder, following the same procedure as described in the general procedure C with *2-([1,1′-biphenyl]-4-yl)-3-hydroxy-6,7-dimethoxy-4H-chromen-4-one* (**19 m**) (300 mg, 0.80 mmol) and boron tribromide (4.0 mL, 5 eq) in dichloromethane (20 mL), stirring for 16 h at 50 °C. The crude residue was purified by column chromatography on silica gel (CH_2_Cl_2_/MeOH = 30:1 to 10:1). R_f_ = 0.33 (CH_2_Cl_2/_MeOH = 10:1). ^1^H NMR (600 MHz, DMSO) δ 10.49 (s, 1H), 9.78 (s, 1H), 9.28 (s, 1H), 8.27 (d, *J* = 8.6 Hz, 2H), 7.86 (d, *J* = 8.6 Hz, 2H), 7.77 (d, *J* = 7.3 Hz, 2H), 7.52 (t, *J* = 7.7 Hz, 2H), 7.42 (t, *J* = 7.4 Hz, 1H), 7.33 (s, 1H), 7.01 (s, 1H); ^13^C NMR (150 MHz, DMSO) δ 172.24, 153.11, 150.72, 144.89, 143.91, 141.16, 139.73, 138.72, 131.22, 129.53, 128.42, 128.25, 127.20, 127.11, 114.57, 107.41, 103.16, 31.16. HRMS *m*/*z* calculated for C_21_H_14_O_5_ [M – H]^–^: 345.0708; found: 345.0739. >95% purity (as determined by RP-HPLC, method C, *t*_R_ = 13.94 min).

##### 3,6,7-Trihydroxy-2–(4-phenoxyphenyl)-4H-chromen-4-one (20n)

Compound **20n** was prepared in 41% yield as a brown powder, following the same procedure as described in the general procedure C with *3-hydroxy-6,7-dimethoxy-2–(4-phenoxyphenyl)-4H-chromen-4-one* (**19n**) (70 mg, 0.19 mmol) and boron tribromide (2.29 mL, 12 eq) in dichloromethane (20 mL), stirring for 6 h at 50 °C. The crude residue was purified by column chromatography on silica gel (CH_2_Cl_2_/MeOH = 50:1 to 4:1). R_f_ = 0.28 (CH_2_Cl_2/_MeOH = 10:1). ^1^H NMR (600 MHz, DMSO) δ 10.49 (s, 1H), 9.77 (s, 1H), 9.14 (s, 1H), 8.17 (d, *J* = 8.9 Hz, 2H), 7.44 (t, *J* = 7.9 Hz, 2H), 7.32 (s, 1H), 7.20 (t, *J* = 7.4 Hz, 1H), 7.12 (dd, *J* = 20.6, 8.4 Hz, 4H), 6.97 (s, 1H); ^13^C NMR (150 MHz, DMSO) δ 171.74, 157.63, 155.91, 152.54, 150.17, 144.41, 143.60, 137.61, 130.27, 130.25, 129.34, 126.66, 124.13, 119.25, 118.07, 114.10, 106.99, 102.66. HRMS *m*/*z* calculated for C_21_H_14_O_6_ [M – H]^–^: 361.0717; found: 361.0716. >95% purity (as determined by RP-HPLC, method C, *t*_R_ = 11.28 min).

##### 3,6,7-Trihydroxy-2–(3-phenoxyphenyl)-4H-chromen-4-one (20o)

Compound **20o** was prepared in 22% yield as a brown powder, following the same procedure as described in the general procedure C with *3-hydroxy-6,7-dimethoxy-2–(3-phenoxyphenyl)-4H-chromen-4-one* (**19o**) (180 mg, 0.46 mmol) and boron tribromide (2.29 mL, 12 eq) in dichloromethane (20 mL), stirring for 16 h at 50 °C. The crude residue was purified by column chromatography on silica gel (CH_2_Cl_2_/MeOH = 50:1 to 10:1). R_f_ = 0.48 (CH_2_Cl_2/_MeOH = 10:1). ^1^H NMR (600 MHz, DMSO) δ 7.94 (d, *J* = 8.0 Hz, 1H), 7.88 − 7.81 (m, 1H), 7.54 (t, *J* = 7.9 Hz, 1H), 7.42 (t, *J* = 8.0 Hz, 2H), 7.30 (d, *J* = 8.0 Hz, 1H), 7.17 (t, 1H), 7.08 (dd, *J* = 14.0, 4.9 Hz, 3H), 6.94 (s, 1H); ^13^C NMR (150 MHz, DMSO) δ 171.80, 156.57, 156.55, 152.83, 150.24, 144.51, 142.68, 138.40, 133.61, 130.26, 130.19, 130.14, 124.12, 123.61, 122.31, 119.48, 118.57, 117.55, 113.97, 106.86, 102.62. HRMS *m*/*z* calculated for C_21_H_14_O_6_ [M – H]^–^: 361.0717; found: 361.0727. >95% purity (as determined by RP-HPLC, method C, *t*_R_ = 11.54 min).

##### 3,6,7-Trihydroxy-2–(4-hydroxyphenyl)-4H-chromen-4-one (20p)

Compound **20p** was prepared in 89% yield as a brown powder, following the same procedure as described in the general procedure C with *3-hydroxy-6,7-dimethoxy-2–(4-methoxyphenyl)-4H-chromen-4-one* (**19p**) (180 mg, 0.633 mmol) and boron tribromide (6.33 mL, 10 eq) in dichloromethane (20 mL), stirring for 15 h at 50 °C. The reaction mixture was filtered through a filter paper and concentrated under reduced pressure. The crude residue was purified by column chromatography on silica gel (CH_2_Cl_2_/MeOH = 20:1 to 4:1). R_f_ = 0.30 (CH_2_Cl_2/_MeOH = 10:1). ^1^H NMR (600 MHz, DMSO) δ 10.41 (s, 1H), 9.98 (s, 1H), 9.71 (s, 1H), 8.87 (s, 1H), 8.01 (d, *J* = 7.9 Hz, 2H), 7.30 (s, 1H), 6.99 − 6.87 (m, 3H). HRMS *m*/*z* calculated for C_15_H_10_O_6_ [M – H]^–^: 285.0404; found: 285.0399.

##### 3,6,7-Trihydroxy-2–(3-hydroxyphenyl)-4H-chromen-4-one (20q)

Compound **20q** was prepared in 87% yield as a brown powder, following the same procedure as described in the general procedure C with *3-hydroxy-6,7-dimethoxy-2–(3-methoxyphenyl)-4H-chromen-4-one* (**19q**) (96 mg, 0.292 mmol) and boron tribromide (4.38 mL, 15 eq) in dichloromethane (20 mL), stirring for 15 h at 50 °C. The crude residue was purified by column chromatography on silica gel (CH_2_Cl_2_/MeOH = 20:1 to 4:1). R_f_ = 0.32 (CH_2_Cl_2/_MeOH = 10:1). ^1^H NMR (600 MHz, DMSO) δ 7.61 − 7.58 (m, 2H), 7.32 (d, *J* = 14.4 Hz, 2H), 6.91 (s, 1H), 6.85 (d, *J* = 6.0 Hz 1H). HRMS *m*/*z* calculated for C_15_H_10_O_6_ [M – H]^–^: 285.0404; found: 285.0410.

##### 4–(3,6,7-Trihydroxy-4-oxo-4H-chromen-2-yl)benzoic acid (20r)

Compound **20r** was prepared in 9% yield as a brown powder, following the same procedure as described in the general procedure C with *methyl 4–(3-hydroxy-6,7-dimethoxy-4-oxo-4H-chromen-2-yl)benzoate* (**19r**) (80 mg, 0.22 mmol) and boron tribromide (3.36 mL, 15 eq) in dichloromethane (20 mL), stirring for 16 h at 50 °C. The crude residue was recrystallized from methanol. R_f_ = 0.05 (CH_2_Cl_2/_MeOH = 10:1). ^1^H NMR (600 MHz, MeOD) δ 8.33 (d, *J* = 8.4 Hz, 2H), 8.13 (d, *J* = 8.6 Hz, 2H), 7.41 (s, 1H), 7.01 (s, 1H); ^13^C NMR (150 MHz, DMSO) δ 171.83, 165.84, 153.50, 150.53, 144.77, 142.02, 139.24, 136.22, 129.53, 129.25, 127.26, 113.81, 106.64, 102.49. HRMS *m*/*z* calculated for C_15_H_10_Cl_2_O_5_ [M – H]^–^: 313.0354; found: 313.0342. >95% purity (as determined by RP-HPLC, method G, *t*_R_ = 8.65 min).

##### 3–(3,6,7-Trihydroxy-4-oxo-4H-chromen-2-yl)benzoic acid (20s)

Compound **20s** was prepared in 26% yield as a brown powder, following the same procedure as described in the general procedure C with *methyl methyl 3–(3-hydroxy-6,7-dimethoxy-4-oxo-4H-chromen-2-yl)benzoate* (**19s**) (76 mg, 0.21 mmol) and boron tribromide (3.20 mL, 15 eq) in dichloromethane (20 mL), stirring for 16 h at 50 °C. The crude residue was recrystallized from methanol. R_f_ = 0.08 (CH_2_Cl_2/_MeOH = 10:1). ^1^H NMR (600 MHz, MeOD) δ 8.87 (t, *J* = 1.6 Hz, 1H), 8.51 − 8.42 (m, 1H), 8.13 − 8.05 (m, 1H), 7.63 (t, *J* = 7.8 Hz, 1H), 7.41 (d, *J* = 4.3 Hz, 1H), 7.02 (s, 1H); ^13^C NMR (150 MHz, DMSO) δ 171.86, 167.06, 152.79, 150.29, 144.52, 142.70, 138.50, 132.13, 131.09, 131.05, 129.93, 128.95, 128.26, 114.11, 106.96, 102.65. HRMS *m*/*z* calculated for C_16_H_10_O_7_ [M – H]^–^: 313.0354; found: 313.0365. >95% purity (as determined by RP-HPLC, method G, *t*_R_ = 10.22 min).

##### 3,6,7-Trihydroxy-2-(naphthalen-2-yl)-4H-chromen-4-one (23a)

Compound **23a** was prepared in 57% yield as a brown powder, following the same procedure as described in the general procedure C with *3-hydroxy-6,7-dimethoxy-2-(naphthalen-2-yl)-4H-chromen-4-one* (**22a**) (35 mg, 0.11 mmol) and boron tribromide (1.07 mL, 10 eq) in dichloromethane (10 mL), stirring for 16 h at 50 °C. The crude residue was purified by column chromatography on silica gel (CH_2_Cl_2_/MeOH = 30:1 to 4:1). R_f_ = 0.45 (CH_2_Cl_2/_MeOH = 10:1). ^1^H NMR (600 MHz, DMSO) δ 8.75 (s, 1H), 8.29 (dd, *J* = 8.7, 1.6 Hz, 1H), 8.06 (d, *J* = 8.8 Hz, 2H), 8.02 − 7.96 (m, 1H), 7.66 − 7.52 (m, 2H), 7.36 (s, 1H), 7.06 (s, 1H); ^13^C NMR (150 MHz, DMSO) δ 172.29, 153.14, 150.84, 144.93, 144.17, 138.91, 133.38, 133.00, 129.72, 129.23, 128.36, 128.03, 127.75, 127.70, 127.17, 124.74, 114.60, 107.43, 103.20. HRMS *m*/*z* calculated for C_19_H_12_O_5_ [M – H]^–^: 319.0612; found: 319.0609. >95% purity (as determined by RP-HPLC, method C, *t*_R_ = 12.43 min).

##### 3,6,7-Trihydroxy-2–(1-methyl-1H-pyrazol-4-yl)-4H-chromen-4-one (23b)

Compound **23b** was prepared in 27% yield as a brown powder, following the same procedure as described in the general procedure C with *3-hydroxy-6,7-dimethoxy-2–(1-methyl-1H-pyrazol-4-yl)-4H-chromen-4-one* (**22b**) (177 mg, 0.59 mmol) and boron tribromide (5.85 mL, 10 eq) in dichloromethane (10 mL), stirring for 16 h at 50 °C. The crude residue was purified by column chromatography on silica gel (CH_2_Cl_2_/MeOH = 30:1 to 4:1). R_f_ = 0.34 (CH_2_Cl_2/_MeOH = 10:1). ^1^H NMR (600 MHz, DMSO) δ 10.35 (s, 1H), 9.65 (s, 1H), 9.10 (s, 1H), 8.30 (s, 1H), 8.01 (s, 1H), 7.30 (s, 1H), 6.91 (s, 1H), 3.94 (s, 3H); ^13^C NMR (150 MHz, DMSO) δ 171.29, 152.32, 150.05, 144.53, 142.53, 137.77, 135.76, 130.99, 114.99, 114.20, 107.65, 103.06, 49.07. HRMS *m*/*z* calculated for C_13_H_10_N_2_O_5_ [M – H]^–^: 273.0524; found: 273.0523. >95% purity (as determined by RP-HPLC, method H, t_R_ = 5.97 min).

##### 3,6,7-Trihydroxy-2-(thiophen-2-yl)-4H-chromen-4-one (23c)

Compound **23c** was prepared in 39% yield as a brown powder, following the same procedure as described in the general procedure C with *3-hydroxy-6,7-dimethoxy-2-(thiophen-2-yl)-4H-chromen-4-one* (**22c**) (75 mg, 0.25 mmol) and boron tribromide (2.46 mL, 10 eq) in dichloromethane (10 mL), stirring for 16 h at 50 °C. The crude residue was purified by column chromatography on silica gel (CH_2_Cl_2_/MeOH = 30:1 to 4:1). R_f_ = 0.45 (CH_2_Cl_2/_MeOH = 10:1). ^1^H NMR (600 MHz, DMSO) δ 10.47 (s, 1H), 9.79 (s, 1H), 9.75 (s, 1H), 7.91 − 7.85 (m, 1H), 7.83 (dd, *J* = 5.0, 0.8 Hz, 1H), 7.32 (s, 1H), 7.27 (dd, *J* = 4.9, 3.9 Hz, 1H), 6.95 (s, 1H); ^13^C NMR (150 MHz, DMSO) δ 171.55, 152.85, 150.13, 144.75, 142.36, 136.32, 133.29, 130.33, 128.13, 127.88, 114.93, 107.65, 103.04. HRMS *m*/*z* calculated for [M – H]^–^: 275.0019; found: 275.0026. >95% purity (as determined by RP-HPLC, method F, t_R_ = 10.00 min).

#### General Procedure D for compounds 18a–18s

To a stirred solution of substituted acetophenone compound in tetrahydrofuran (THF) was added appropriate aldehyde (1.2 eq or 1.5 eq) at room temperature. The reaction mixture was added sodium methoxide (5.4 M, 1.2 eq) in methanol solution at 0 °C and stirred for 5 min. The reaction mixture was warmed to room temperature and stirred at room temperature until complete conversion monitored by TLC analysis (typically 12–16 h), quenched with acetic acid, and extracted with EtOAc and H_2_O. The organic layer was washed with saturated aqueous NaHCO_3_, dried over MgSO_4_, filtered, and concentrated under reduced pressure. The crude residue was purified by column chromatography on a silica gel to furnish compounds **18a–18t.**

##### (E)-3–(3,4-Dimethoxyphenyl)-1–(2-hydroxy-4,5-dimethoxyphenyl)prop-2-en-1-one (18a)

Compound **18a** was prepared in 53% yield as a yellow powder, following the same procedure as described in the general procedure D with *1–(2-hydroxy-4,5-dimethoxyphenyl)ethanone* **(11)** (200 mg, 1.02 mmol), 3,4-dimethoxybenzaldehyde (202 mg, 1.2 eq), sodium methoxide solution (0.23 mL, 1.2 eq) in THF (20 mL), stirring for 16 h at room temperature. The crude residue was purified by column chromatography on a silica gel (hexane/EtOAc = 4:1 to 1:1). R_f_ = 0.54 (hexane/EtOAc = 2:1). ^1^H NMR (600 MHz, CDCl_3_) δ 7.87 (d, *J* = 15.3 Hz, 1H), 7.37 (d, *J* = 15.3 Hz, 1H), 7.28 (t, *J* = 4.1 Hz, 2H), 7.16 (s, 1H), 6.93 (d, *J* = 8.3 Hz, 1H), 6.52 (s, 1H), 3.97 (s, 3H), 3.94 (d, *J* = 3.1 Hz, 6H), 3.92 (s, 3H). HRMS *m*/*z* calculated for C_19_H_20_O_6_ [M + H]^+^: 345.1333; found: 345.1336.

##### (E)-1–(2-Hydroxy-4,5-dimethoxyphenyl)-3-phenylprop-2-en-1-one (18b)

Compound **18b** was prepared in 97% yield as a yellow powder, following the same procedure as described in the general procedure D with *1–(2-hydroxy-4,5-dimethoxyphenyl)ethanone* **(11)** (300 mg, 1.53 mmol), benzaldehyde (187 μL, 1.2 eq), sodium methoxide solution (0.33 mL, 1.2 eq) in THF (20 mL), stirring for 16 h at room temperature. The crude residue was purified by column chromatography on silica gel (hexane/EtOAc = 10:1 to 6:1). R_f_ = 0.63 (hexane/EtOAc = 4:1). ^1^H NMR (600 MHz, CDCl_3_) δ 7.91 (d, *J* = 15.4 Hz, 1H), 7.66 (dd, *J* = 6.4, 2.7 Hz, 2H), 7.51 (d, *J* = 11.7 Hz, 1H), 7.46 − 7.40 (m, 3H), 6.52 (s, 1H), 3.94 (s, 9H), 3.92 (s, 3H). HRMS *m*/*z* calculated for C_17_H_16_O_4_ [M + H]^+^: 285.1122; found: 285.1112.

##### (E)-1–(2-Hydroxy-4,5-dimethoxyphenyl)-3-(p-tolyl)prop-2-en-1-one (18c)

Compound **18c** was prepared in 85% yield as a yellow powder, following the same procedure as described in the general procedure D with *1–(2-hydroxy-4,5-dimethoxyphenyl)ethanone* **(11)** (200 mg, 1.02 mmol), benzaldehyde (252 μL, 1.05 eq), sodium methoxide solution (0.56 mL, 1.2 eq) in THF (10 mL), stirring for 15 h at room temperature. The crude residue was purified by column chromatography on silica gel (hexane/EtOAc = 20:1 to 3:1). R_f_ = 0.30 (hexane/EtOAc = 4:1). ^1^H NMR (600 MHz, CDCl_3_) δ 13.41 (s, 1H), 7.89 (d, *J* = 15.4 Hz, 1H), 7.57 (d, *J* = 8.1 Hz, 2H), 7.48 (d, *J* = 15.4 Hz, 1H), 7.26 (d, *J* = 1.5 Hz, 2H), 7.24 (d, *J* = 2.7 Hz, 1H), 6.52 (s, 1H), 3.94 (s, 3H), 3.92 (s, 3H), 2.41 (s, 3H). HRMS *m*/*z* calculated for C_18_H_18_O_4_ [M + H]^+^: 299.1278; found: 299.1283.

##### (E)-1–(2-Hydroxy-4,5-dimethoxyphenyl)-3-(m-tolyl)prop-2-en-1-one (18d)

Compound **18d** was prepared in 65% yield as a yellow powder, following the same procedure as described in the general procedure D with *1–(2-hydroxy-4,5-dimethoxyphenyl)ethanone* **(11)** (500 mg, 2.55 mmol), *m-*tolubenzaldehyde (445 μL, 1.5 eq), sodium methoxide solution (0.7 mL, 1.5 eq) in THF (20 mL), stirring for 15 h at room temperature. The crude residue was recrystallized from ethyl acetate and hexane. R_f_ = 0.35 (hexane/EtOAc = 3:1). ^1^H NMR (600 MHz, CDCl_3_) δ 7.88 (d, *J* = 15.4 Hz, 1H), 7.49 (dd, *J* = 16.3, 11.6 Hz, 3H), 7.33 (t, *J* = 7.6 Hz, 1H), 7.27 (s, 1H), 7.24 (s, 1H), 6.52 (s, 1H), 3.94 (s, 3H), 3.92 (s, 3H), 2.42 (s, 3H). HRMS *m*/*z* calculated for C_18_H_18_O_4_ [M + H]^+^: 299.1278; found: 299.1270.

##### (E)-3–(3,4-Dimethylphenyl)-1–(2-hydroxy-4,5-dimethoxyphenyl)prop-2-en-1-one (18e)

Compound **18e** was prepared in 74% yield as a yellow powder, following the same procedure as described in the general procedure D with *1–(2-hydroxy-4,5-dimethoxyphenyl)ethanone* **(11)** (500 mg, 2.55 mmol), 3,4-dimethylbenzaldehyde (405 μL, 1.2 eq), sodium methoxide solution (0.56 mL, 1.2 eq) in THF (20 mL), stirring for 12 h at room temperature. The crude residue was recrystallized from ethyl acetate and hexane. R_f_ = 0.45 (hexane/EtOAc = 3:1). ^1^H NMR (600 MHz, CDCl_3_) δ 7.87 (d, *J* = 15.4 Hz, 1H), 7.47 (d, *J* = 15.4 Hz, 1H), 7.44 − 7.39 (m, 2H), 7.28 (d, *J* = 4.5 Hz, 1H), 7.20 (d, *J* = 7.7 Hz, 1H), 6.51 (s, 1H), 3.94 (s, 3H), 3.92 (s, 3H), 2.33 (s, 3H), 2.32 (s, 3H). HRMS *m*/*z* calculated for C_19_H_20_O_4_ [M + H]^+^: 313.1435; found: 313.1426.

##### (E)-3–(4-Fluorophenyl)-1–(2-hydroxy-4,5-dimethoxyphenyl)prop-2-en-1-one (18f)

Compound **18f** was prepared in 81% yield as a yellow powder, following the same procedure as described in the general procedure D with *1–(2-hydroxy-4,5-dimethoxyphenyl)ethanone* **(11)** (300 mg, 1.53 mmol), 4-fluorobenzaldehyde (196 μL, 1.2 eq), sodium methoxide solution (0.33 mL, 1.2 eq) in THF (20 mL), stirring for 12 h at room temperature. The crude residue was recrystallized from ethyl acetate and hexane. R_f_ = 0.28 (hexane/EtOAc = 3:1). ^1^H NMR (600 MHz, CDCl_3_) δ 7.91 (d, *J* = 15.4 Hz, 1H), 7.66 (dd, *J* = 6.4, 2.7 Hz, 2H), 7.52 (d, *J* = 15.4 Hz, 1H), 7.47 − 7.40 (m, 3H), 6.52 (s, 1H), 3.94 (s, 3H), 3.92 (s, 3H). HRMS *m*/*z* calculated for C_17_H_15_FO_4_ [M + H]^+^: 303.1027; found: 303.1032.

##### (E)-3–(3-Fluorophenyl)-1–(2-hydroxy-4,5-dimethoxyphenyl)prop-2-en-1-one (18g)

Compound **18 g** was prepared in 91% yield as a yellow powder, following the same procedure as described in the general procedure D with *1–(2-hydroxy-4,5-dimethoxyphenyl)ethanone* **(11)** (400 mg, 2.04 mmol), 3-fluorobenzaldehyde (324 μL, 1.5 eq), sodium methoxide solution (0.56 mL, 1.5 eq) in THF (20 mL), stirring for 12 h at room temperature. The crude residue was recrystallized from ethyl acetate and hexane. R_f_ = 0.46 (hexane/EtOAc = 3:1). ^1^H NMR (600 MHz, CDCl_3_) δ 7.85 (d, *J* = 15.4 Hz, 1H), 7.50 (d, *J* = 15.4 Hz, 1H), 7.44 − 7.38 (m, 2H), 7.36 (d, *J* = 9.5 Hz, 1H), 7.24 (d, *J* = 3.9 Hz, 1H), 7.18 − 7.10 (m, 1H), 6.52 (s, 1H), 3.95 (s, 3H), 3.92 (s, 3H). HRMS *m*/*z* calculated for C_17_H_15_FO_4_ [M + H]^+^: 303.1027; found: 303.1025.

##### (E)-3–(3,4-Difluorophenyl)-1–(2-hydroxy-4,5-dimethoxyphenyl)prop-2-en-1-one (18h)

Compound **18h** was prepared in 56% yield as a yellow powder, following the same procedure as described in the general procedure D with *1–(2-hydroxy-4,5-dimethoxyphenyl)ethanone* **(11)** (400 mg, 2.04 mmol), 3,4-difluorobenzaldehyde (337 μL, 1.5 eq), sodium methoxide solution (0.56 mL, 1.5 eq) in THF (20 mL), stirring for 16 h at room temperature. The crude residue was recrystallized from ethyl acetate and hexane. R_f_ = 0.52 (hexane/EtOAc = 3:1). ^1^H NMR (600 MHz, CDCl_3_) δ 7.81 (d, *J* = 14.2 Hz, 1H), 7.54 − 7.46 (m, *J* = 15.5, 8.7, 5.0 Hz, 1H), 7.44 − 7.34 (m, 2H), 7.25 − 7.19 (m, 2H), 6.52 (s, 1H), 3.95 (s, 3H), 3.92 (s, 3H). HRMS *m*/*z* calculated for C_17_H_14_F_2_O_4_ [M + H]^+^: 321.0933; found: 321.0918.

##### (E)-1–(2-Hydroxy-4,5-dimethoxyphenyl)-3–(4-(trifluoromethyl)phenyl)prop-2-en-1-one (18i)

Compound **18i** was prepared in 89% yield as a yellow powder, following the same procedure as described in the general procedure D with *1–(2-hydroxy-4,5-dimethoxyphenyl)ethanone* **(11)** (200 mg, 1.02 mmol), 4-(Trifluoromethyl)benzaldehyde (508 μL, 1.5 eq), sodium methoxide solution (0.7 mL, 1.5 eq) in THF (20 mL), stirring for 16 h at room temperature. The crude residue was recrystallized from ethyl acetate and hexane. R_f_ = 0.49 (hexane/EtOAc = 4:1). ^1^H NMR (600 MHz, CDCl_3_) δ 7.90 (d, *J* = 15.5 Hz, 1H), 7.76 (d, *J* = 8.3 Hz, 2H), 7.70 (d, *J* = 8.3 Hz, 2H), 7.57 (d, 1H), 7.24 (s, 1H), 6.53 (s, 1H), 3.95 (s, 3H), 3.93 (s, 3H). HRMS *m*/*z* calculated for C_18_H_15_F_3_O_4_ [M + H]^+^: 353.0995; found: 353.0988.

##### (E)-1–(2-Hydroxy-4,5-dimethoxyphenyl)-3–(3-(trifluoromethyl)phenyl)prop-2-en-1-one (18j)

Compound **18j** was prepared in 67% yield as a yellow powder, following the same procedure as described in the general procedure D with *1–(2-hydroxy-4,5-dimethoxyphenyl)ethanone* **(11)** (200 mg, 1.02 mmol), 3-(Trifluoromethyl)benzaldehyde (508 μL, 1.5 eq), sodium methoxide solution (0.7 mL, 1.5 eq) in THF (20 mL), stirring for 16 h at room temperature. The crude residue was recrystallized from ethyl acetate and hexane. R_f_ = 0.33 (hexane/EtOAc = 4:1). ^1^H NMR (600 MHz, CDCl_3_) δ 7.91 (d, *J* = 15.4 Hz, 2H), 7.82 (d, *J* = 7.7 Hz, 1H), 7.68 (d, *J* = 7.7 Hz, 1H), 7.57 (dd, *J* = 18.3, 11.6 Hz, 2H), 7.25 (s, 1H), 6.53 (s, 1H), 3.95 (s, 3H), 3.93 (s, 3H). HRMS *m*/*z* calculated for C_18_H_15_F_3_O_4_ [M + H]^+^: 353.0995; found: 353.0982.

##### (E)-3–(4-Chlorophenyl)-1–(2-hydroxy-4,5-dimethoxyphenyl)prop-2-en-1-one (18k)

Compound **18k** was prepared in 86% yield as a yellow powder, following the same procedure as described in the general procedure D with *1–(2-hydroxy-4,5-dimethoxyphenyl)ethanone* **(11)** (300 mg, 1.53 mmol), 4-chlorobenzaldehyde (257 mg, 1.2 eq), sodium methoxide solution (0.33 mL, 1.2 eq) in THF (20 mL), stirring for 12 h at room temperature. The crude residue was recrystallized from ethyl acetate and hexane. R_f_ = 0.24 (hexane/EtOAc = 3:1). ^1^H NMR (600 MHz, CDCl_3_) δ 7.85 (d, *J* = 15.4 Hz, 1H), 7.59 (d, *J* = 8.4 Hz, 2H), 7.48 (d, *J* = 15.4 Hz, 1H), 7.41 (d, *J* = 8.4 Hz, 2H), 7.24 (s, 1H), 6.52 (s, 1H), 3.94 (s, 3H), 3.92 (s, 3H). HRMS *m*/*z* calculated for C_17_H_15_ClO_4_ [M + H]^+^: 319.0732; found: 319.0736.

##### (E)-3–(3-Chlorophenyl)-1–(2-hydroxy-4,5-dimethoxyphenyl)prop-2-en-1-one (18l)

Compound **18 l** was prepared in 68% yield as a yellow powder, following the same procedure as described in the general procedure D with *1–(2-hydroxy-4,5-dimethoxyphenyl)ethanone* **(11)** (500 mg, 2.55 mmol), 3-chlorobenzaldehyde (346 μL, 1.2 eq), sodium methoxide solution (0.56 mL, 1.2 eq) in THF (20 mL), stirring for 12 h at room temperature. The crude residue was recrystallized from ethyl acetate and hexane. R_f_ = 0.50 (hexane/EtOAc = 3:1). ^1^H NMR (600 MHz, CDCl_3_) δ 7.82 (d, *J* = 15.4 Hz, 1H), 7.65 (s, 1H), 7.51 (t, *J* = 10.4 Hz, 2H), 7.42 − 7.36 (m, 2H), 7.23 (s, 1H), 6.52 (s, 1H), 3.95 (s, 3H), 3.93 (s, 3H). HRMS *m*/*z* calculated for C_17_H_15_ClO_4_ [M + H]^+^: 319.0732; found: 319.0746.

##### (E)-3-([1,1'-Biphenyl]-4-yl)-1–(2-hydroxy-4,5-dimethoxyphenyl)prop-2-en-1-one (18m)

Compound **18 m** was prepared in 52% yield as a yellow powder, following the same procedure as described in the general procedure D with *1–(2-hydroxy-4,5-dimethoxyphenyl)ethanone* **(11)** (500 mg, 2.55 mmol), Biphenyl-4-carboxaldehyde (557 mg, 1.2 eq), sodium methoxide solution (0.7 mL, 1.5 eq) in THF (20 mL), stirring for 16 h at room temperature. The crude residue was recrystallized from ethyl acetate and hexane. R_f_ = 0.44 (hexane/EtOAc = 3:1). ^1^H NMR (600 MHz, CDCl_3_) δ 7.95 (d, *J* = 15.4 Hz, 1H), 7.75 (d, *J* = 8.3 Hz, 2H), 7.68 (d, *J* = 8.3 Hz, 2H), 7.66 − 7.62 (m, 2H), 7.56 (d, *J* = 15.4 Hz, 1H), 7.50 − 7.45 (m, 2H), 7.41 − 7.37 (m, 1H), 7.29 (s, 1H), 6.53 (s, 1H), 3.95 (s, 3H), 3.94 (s, 3H). HRMS *m*/*z* calculated for C_23_H_20_O_4_ [M + H]^+^: 361.1417; found: 361.1447.

##### (E)-1–(2-Hydroxy-4,5-dimethoxyphenyl)-3–(4-phenoxyphenyl)prop-2-en-1-one (18n)

Compound **18n** was prepared in 89% yield as a yellow powder, following the same procedure as described in the general procedure D with *1–(2-hydroxy-4,5-dimethoxyphenyl)ethanone* **(11)** (500 mg, 2.55 mmol), 4-phenoxybenzaldehyde (658 μL, 1.5 eq), sodium methoxide solution (0.7 mL, 1.5 eq) in THF (20 mL), stirring for 16 h at room temperature. The crude residue was recrystallized from ethyl acetate and hexane. R_f_ = 0.44 (hexane/EtOAc = 3:1). ^1^H NMR (600 MHz, CDCl_3_) δ 7.89 (d, *J* = 15.4 Hz, 1H), 7.64 (d, *J* = 8.6 Hz, 2H), 7.48 − 7.35 (m, 3H), 7.25 (s, 1H), 7.18 (t, *J* = 7.4 Hz, 1H), 7.08 (d, *J* = 7.8 Hz, 2H), 7.03 (d, *J* = 8.6 Hz, 2H), 6.52 (s, 1H), 3.94 (s, 3H), 3.91 (s, 3H). HRMS *m*/*z* calculated for C_23_H_20_O_5_ [M + H]^+^: 377.1384; found: 377.1384.

##### (E)-1–(2-Hydroxy-4,5-dimethoxyphenyl)-3–(3-phenoxyphenyl)prop-2-en-1-one (18o)

Compound **18o** was prepared in 35% yield as a yellow powder, following the same procedure as described in the general procedure D with *1–(2-hydroxy-4,5-dimethoxyphenyl)ethanone* **(11)** (500 mg, 2.55 mmol), 3-phenoxybenzaldehyde (658 μL, 1.5 eq), sodium methoxide solution (0.7 mL, 1.5 eq) in THF (20 mL), stirring for 16 h at room temperature. The crude residue was recrystallized from ethyl acetate and hexane. R_f_ = 0.44 (hexane/EtOAc = 3:1). ^1^H NMR (600 MHz, CDCl_3_) δ 7.82 (d, *J* = 11.7 Hz, 1H), 7.46 (d, *J* = 11.7 Hz, 1H), 7.39 − 7.35 (m, 3H), 7.31 (s, 1H), 7.21 (s, 1H), 7.15 (t, *J* = 7.4 Hz, 1H), 7.10 − 7.02 (m, 2H), 6.51 (s, 2H), 3.94 (s, 3H), 3.90 (s, 3H). HRMS *m*/*z* calculated for C_23_H_20_O_5_ [M + H]^+^: 377.1384; found: 377.1393.

##### (E)-3–(4-Methoxyphenyl)-1–(2-hydroxy-4,5-dimethoxyphenyl)prop-2-en-1-one (18p)

Compound **18p** was prepared in 84% yield as a yellow powder, following the same procedure as described in the general procedure D with *1–(2-hydroxy-4,5-dimethoxyphenyl)ethanone* **(11)** (2350 mg, 12 mmol), *p*-anisaldehyde (1.747 mL, 1.2 eq), sodium methoxide solution (2.66 mL, 1.2 eq) in THF (36 mL), stirring for 16 h at room temperature. The crude residue was recrystallized from ethyl acetate and hexane. R_f_ = 0.33 (hexane/EtOAc = 3:1). ^1^H NMR (600 MHz, CDCl_3_) 7.88 (d, *J* = 15.3 Hz, 1H), 7.63 (d, *J* = 8.7 Hz, 2H), 7.41 (d, *J* = 13.8 Hz, 1H), 7.26 (s, 1H), 6.96 (d, *J* = 8.7 Hz, 2H), 6.51 (s, 1H), 3.94 (s, 3H), 3.92 (s, 3H), 3.87 (s, 3H). HRMS (ESI) m/z calculated for C_18_H_18_O_5_ [M + H]^+^: 315.1227; found: 315.1141.

##### (E)-3–(3-Methoxyphenyl)-1–(2-hydroxy-4,5-dimethoxyphenyl)prop-2-en-1-one (18q)

Compound **18q** was prepared in 35% yield as a yellow powder, following the same procedure as described in the general procedure D with *1–(2-hydroxy-4,5-dimethoxyphenyl)ethanone* **(11)** (500 mg, 12 mmol), *m*-anisaldehyde (465 μL, 1.5 eq), sodium methoxide solution (0.84 mL, 1.5 eq) in THF (20 mL), stirring for 16 h at room temperature. The crude residue was purified by column chromatography on a silica gel (hexane/EtOAc = 10:1 to 4:1). Rf= 0.41 (hexane-E.A = 2:1, v/v). 1 H NMR (300 MHz, CDCl3) δ 13.48 (s, 1H), 7.88 (d, J = 15.3 Hz, 1H), 7.64 (d, J = 8.7 Hz, 1H), 7.63 (dd, J = 4.5 and 9.6 Hz, 1H), 7.41 (d, J = 15.3 Hz, 1H), 6.97 (d, J = 8.7 Hz, 1H), 6.96 (dd, J = 4.5 and 9.6 Hz, 1H), 6.51 (s, 1H), 3.94 (s, 3H), 3.92 (s, 3H), 3.87 (s, 3H). HRMS (ESI) m/z calculated for C_18_H_18_O_5_ [M + H]^+^: 315.1227; found: 315.1242.

##### (E)-Methyl 4–(3-(2-hydroxy-4,5-dimethoxyphenyl)-3-oxoprop-1-en-1-yl)benzoate (18r)

Compound **18r** was prepared in 37% yield as a yellow powder, following the same procedure as described in the general procedure D with *1–(2-hydroxy-4,5-dimethoxyphenyl)ethanone* **(11)** (2350 mg, 12 mmol), Methyl terepthalaldehydate (2360 mg, 1.2 eq), sodium methoxide solution (3.28 mL, 1.2 eq) in THF (36 mL), stirring for 16 h at room temperature. The crude residue was recrystallized from ethyl acetate and hexane. R_f_ = 0.61 (hexane/EtOAc = 1:1). ^1^H NMR (600 MHz, CDCl_3_) δ 8.10 (d, *J* = 8.3 Hz, 2H), 7.90 (d, *J* = 15.5 Hz, 1H), 7.72 (d, *J* = 8.3 Hz, 2H), 7.58 (d, *J* = 15.5 Hz, 1H), 7.25 (s, 1H), 6.52 (s, 1H), 3.95 (s, 6H), 3.92 (s, 3H). HRMS *m*/*z* calculated for C_19_H_18_O_6_ [M + H]^+^: 343.1176; found: 343.1179.

##### (E)-Methyl 3–(3-(2-hydroxy-4,5-dimethoxyphenyl)-3-oxoprop-1-en-1-yl)benzoate (18s)

Compound **18s** was prepared in 19% yield as a yellow powder, following the same procedure as described in the general procedure D with *1–(2-hydroxy-4,5-dimethoxyphenyl)ethanone* **(11)** (1000 mg, 5.10 mmol), Methyl 3-formylbenzoate (1000 mg, 1.2 eq), sodium methoxide solution (1.13 mL, 1.2 eq) in THF (20 mL), stirring for 16 h at room temperature The crude residue was recrystallized from ethyl acetate and hexane. R_f_ = 0.49 (hexane/EtOAc = 2:1). ^1^H NMR (600 MHz, CDCl_3_) δ 8.35 (s, 1H), 8.12 − 8.05 (m, 1H), 7.92 (d, *J* = 15.5 Hz, 1H), 7.81 (d, *J* = 7.7 Hz, 1H), 7.58 (d, 1H), 7.53 (t, *J* = 7.7 Hz, 1H), 7.27 (s, 1H), 6.52 (s, 1H), 3.97 (s, 3H), 3.95 (s, 3H), 3.94 (s, 3H). HRMS *m*/*z* calculated for C_19_H_18_O_6_ [M + H]^+^: 343.1176; found: 343.1181.

##### (E)-1–(2-Hydroxy-4,5-dimethoxyphenyl)-3-(naphthalen-2-yl)prop-2-en-1-one (21a)

Compound **21a** was prepared in 54% yield as a yellow powder, following the same procedure as described in the general procedure D with *1–(2-hydroxy-4,5-dimethoxyphenyl)ethanone* **(11)** (500 mg, 2.55 mmol), 2-naphthaldehyde (477 mg, 1.2 eq), sodium methoxide solution (0.56 mL, 1.2 eq) in THF (20 mL), stirring for 16 h at room temperature. The crude residue was recrystallized from ethyl acetate and hexane. R_f_ = 0.32 (hexane/EtOAc = 4:1). ^1^H NMR (600 MHz, CDCl_3_) δ 8.07 (d, *J* = 15.1 Hz, 2H), 7.98 − 7.84 (m, 3H), 7.81 (dd, *J* = 8.5, 1.2 Hz, 1H), 7.63 (d, *J* = 15.4 Hz, 1H), 7.59 − 7.50 (m, 2H), 7.32 (s, 1H), 6.53 (s, 1H), 3.95 (s, 6H). HRMS *m*/*z* calculated for C_21_H_18_O_4_ [M + H]^+^: 335.1278; found: 335.1259.

##### (E)-1–(2-Hydroxy-4,5-dimethoxyphenyl)-3–(1-methyl-1H-pyrazol-4-yl)prop-2-en-1-one (21b)

Compound **21b** was prepared in 54% yield as a yellow powder, following the same procedure as described in the general procedure D with *1–(2-hydroxy-4,5-dimethoxyphenyl)ethanone* **(11)** (500 mg, 1.53 mmol), 1-methypyrazole-4-carboxaldehyde (0.52 mL, 1.5 eq), sodium methoxide solution (0.56 mL, 1.2 eq) in THF (20 mL), stirring for 16 h at room temperature. The crude residue was recrystallized from ethyl acetate and hexane. R_f_ = 0.12 (hexane/EtOAc = 3:1). ^1^H NMR (600 MHz, CDCl_3_) δ 7.85 (s, 1H), 7.81 (d, *J* = 15.0 Hz, 1H), 7.67 (s, 1H), 7.26 (s, 1H), 7.24 (d, *J* = 15.6 Hz, 1H), 3.96 (s, 3H), 3.94 (s, 3H), 3.92 (s, 3H). HRMS *m*/*z* calculated for C_15_H_16_N_2_O_4_ [M + H]^+^: 289.1183; found: 289.1195.

##### (E)-1–(2-Hydroxy-4,5-dimethoxyphenyl)-3-(thiophen-2-yl)prop-2-en-1-one (21c)

Compound **21c** was prepared in 99% yield as a yellow powder, following the same procedure as described in the general procedure D with *1–(2-hydroxy-4,5-dimethoxyphenyl)ethanone* **(11)** (300 mg, 1.53 mmol), 2-thiophenecarboxaldehyde (209 μL, 1.5 eq), sodium methoxide solution (0.43 mL, 1.5 eq) in THF (20 mL), stirring for 16 h at room temperature. The crude residue was recrystallized from ethyl acetate and hexane. R_f_ = 0.34 (hexane/EtOAc = 3:1). ^1^H NMR (600 MHz, CDCl_3_) δ 8.03 (d, *J* = 15.0 Hz, 1H), 7.44 (d, *J* = 3.6 Hz, 1H), 7.39 (d, *J* = 3.6 Hz, 1H), 7.30 (d, *J* = 3.6 Hz, 1H), 7.22 (s, 1H), 7.12 − 7.08 (m, 1H), 6.51 (s, 1H), 3.94 (s, 3H), 3.92 (s, 3H). HRMS *m*/*z* calculated for C_15_H_14_O_4_S [M + H]^+^: 291.0686; found: 291.0678.

#### General Procedure E for compounds 19a–19s and 22a–22c

To a stirred solution of chalcone compound (**19a–19s** and **22a–22c**) in MeOH or EtOH were added sodium hydroxide (NaOH, 3 M aq.) or sodium methoxide (NaOCH_3_, 5.4 M in methanol solution, 4 eq) and hydrogen peroxide (H_2_O_2_, 35% aq., 4 eq) at room temperature. The reaction mixture was stirred under argon at 40 °C until complete conversion monitored by TLC analysis (typically 5–16 h). The reaction mixture was then poured into H_2_O and acidified with 3N HCl (pH = 2) and extracted with CH_2_Cl_2_. The organic layer was dried over MgSO_4_, filtered, and concentrated under reduced pressure. The crude residue was purified by column chromatography on a silica gel furnish compounds **19a–19s** and **22a–22c**.

##### 2–(3,4-Dimethoxyphenyl)-3-hydroxy-6,7-dimethoxy-4H-chromen-4-one (19a)

Compound **19a** was prepared in 68% yield as a yellow powder, following the same procedure as described in the general procedure E with *(E)-3–(3,4-dimethoxyphenyl)-1–(2-hydroxy-4,5-dimethoxyphenyl)prop-2-en-1-one* (**18a**) (185 mg, 0.54 mmol), NaOH (3 M aq.) 2 mL and hydrogen peroxide (35% aq., 209 μL, 4 eq) in ethanol (10 mL), stirring for 16 h. The reaction mixture was poured into H_2_O and acidified with 3N HCl (pH = 2) and extracted with CH_2_Cl_2_. The crude residue was purified by column chromatography on silica gel (CH_2_Cl_2_/MeOH = 100:1 to 8:1). R_f_ = 0.35 (CH_2_Cl_2_/MeOH = 30:1). ^1^H NMR (600 MHz, CDCl_3_) δ 7.91 − 7.78 (m, 2H), 7.53 (s, 1H), 7.05 − 6.95 (m, 3H), 4.03 (s, 3H), 4.00 (s, 6H), 3.98 (d, *J* = 6.4 Hz, 3H). HRMS *m*/*z* calculated for C_19_H_18_O_7_ [M + H]^+^: 359.1126; found: 359.1132.

##### 3-Hydroxy-6,7-dimethoxy-2-phenyl-4H-chromen-4-one (19b)

Compound **19b** was prepared in 24% yield as a yellow powder, following the same procedure as described in the general procedure E with *(E)-1–(2-hydroxy-4,5-dimethoxyphenyl)-3-phenylprop-2-en-1-one* (**18b**) (200 mg, 0.70 mmol), NaOH (3 M aq.) 2 mL and hydrogen peroxide (35% aq., 273 μL, 4 eq) in ethanol (10 mL), stirring for 5 h. The reaction mixture was poured into H_2_O and acidified with 3N HCl (pH = 2) and extracted with CH_2_Cl_2_. The crude residue was purified by column chromatography on silica gel (CH_2_Cl_2_/MeOH = 100:0 to 30:1). R_f_ = 0.39 (CH_2_Cl_2_/MeOH = 100:1). ^1^H NMR (600 MHz, CDCl_3_) δ 8.23 (d, *J* = 7.5 Hz, 2H), 7.57 − 7.51 (m, 3H), 7.46 (t, *J* = 7.4 Hz, 1H), 7.03 (s, 1H), 7.00 (s, 1H), 4.03 (s, 3H), 4.01 (s, 3H). HRMS *m*/*z* calculated for C_17_H_14_O_5_ [M + H]^+^: 299.0554; found: 299.0914.

##### 3-Hydroxy-6,7-dimethoxy-2-(p-tolyl)-4H-chromen-4-one (19c)

Compound **19c** was prepared in 58% yield as a yellow powder, following the same procedure as described in the general procedure E with *(E)-1–(2-hydroxy-4,5-dimethoxyphenyl)-3-(p-tolyl)prop-2-en-1-one* (**18c**) (180 mg, 0.60 mmol), NaOH (3 M aq.) 1.5 mL and hydrogen peroxide (35% aq., 274 μL, 4 eq) in ethanol (10 mL), stirring for 16 h. The reaction mixture was poured into H_2_O and acidified with 3N HCl (pH = 2) and extracted with CH_2_Cl_2_. The crude residue was purified by column chromatography on silica gel (CH_2_Cl_2_/MeOH = 100:0 to 30:1). ^1^H NMR (600 MHz, CDCl_3_) δ 8.13 (d, *J* = 8.2 Hz, 2H), 7.53 (s, 1H), 7.34 (d, *J* = 8.1 Hz, 2H), 6.99 (d, *J* = 4.4 Hz, 2H), 4.03 (s, 3H), 4.00 (s, 3H), 2.44 (s, 3H). HRMS *m*/*z* calculated for C_18_H_16_O_5_ [M + H]^+^: 313.1070; found: 313.1069.

##### 3-Hydroxy-6,7-dimethoxy-2-(m-tolyl)-4H-chromen-4-one (19d)

Compound **19d** was prepared in 16% yield as a yellow powder, following the same procedure as described in the general procedure E with *(E)-1–(2-hydroxy-4,5-dimethoxyphenyl)-3-(m-tolyl)prop-2-en-1-one* (**18d**) (480 mg, 1.60 mmol), NaOH (3 M aq.) 2 mL and hydrogen peroxide (35% aq., 625 μL, 4 eq) in methanol (10 mL), stirring for 20 h. The reaction mixture was poured into H_2_O and acidified with 3N HCl (pH = 2) and extracted with CH_2_Cl_2_. The crude residue was purified by column chromatography on silica gel (CH_2_Cl_2_/MeOH = 100:0 to 30:1). R_f_ = 0.21 (CH_2_Cl_2_/MeOH = 100:1). ^1^H NMR (600 MHz, CDCl_3_) δ 8.04 (d, *J* = 8.9 Hz, 2H), 7.53 (s, 1H), 7.42 (t, *J* = 7.6 Hz, 1H), 7.28 (d, *J* = 7.7 Hz, 1H), 7.01 (s, 2H), 4.03 (s, 3H), 4.01 (s, 3H), 2.47 (s, 3H).

##### 2–(3,4-Dimethylphenyl)-3-hydroxy-6,7-dimethoxy-4H-chromen-4-one (19e)

Compound **19e** was prepared in 32% yield as a yellow powder, following the same procedure as described in the general procedure E with *(E)-3–(3,4-dimethylphenyl)-1–(2-hydroxy-4,5-dimethoxyphenyl)prop-2-en-1-one* (**18e**) (120 mg, 0.38 mmol), NaOH (3 M aq.) 1 mL and hydrogen peroxide (35% aq., 149 μL, 4 eq) in ethanol (10 mL), stirring for 16 h. The reaction mixture was poured into H_2_O and acidified with 3N HCl (pH = 2) and extracted with CH_2_Cl_2_. The crude residue was purified by column chromatography on silica gel (CH_2_Cl_2_/MeOH = 100:0 to 20:1). R_f_ = 0.55 (CH_2_Cl_2_/MeOH = 30:1). ^1^H NMR (600 MHz, CDCl_3_) δ 8.05 − 7.91 (m, 2H), 7.53 (s, 1H), 7.29 (d, *J* = 8.1 Hz, 1H), 7.00 (s, 1H), 6.97 (s, 1H), 4.03 (s, 3H), 4.00 (s, 3H), 2.38 (s, 3H), 2.35 (s, 3H). (s, 3H), 4.01 (s, 3H), 2.47 (s, 3H). HRMS *m*/*z* calculated for C_19_H_18_O_5_ [M + H]^+^: 327.1227; found: 327.1207.

##### 2–(4-Fluorophenyl)-3-hydroxy-6,7-dimethoxy-4H-chromen-4-one (19f)

Compound **19f** was prepared in 33% yield as a yellow powder, following the same procedure as described in the general procedure E with *(E)-3–(4-fluorophenyl)-1–(2-hydroxy-4,5-dimethoxyphenyl)prop-2-en-1-one* (**18f**) (300 mg, 0.99 mmol), NaOH (3 M aq.) 2 mL and hydrogen peroxide (35% aq., 386 μL, 4 eq) in ethanol (10 mL), stirring for 16 h. The reaction mixture was poured into H_2_O and acidified with 3N HCl (pH = 2) and extracted with CH_2_Cl_2_. The crude residue was purified by column chromatography on silica gel (CH_2_Cl_2_/MeOH = 100:0 to 30:1). R_f_ = 0.29 (CH_2_Cl_2_/MeOH = 30:1). ^1^H NMR (600 MHz, CDCl_3_) δ 8.25 (dd, *J* = 8.8, 5.4 Hz, 2H), 7.53 (s, 1H), 7.22 (t, *J* = 8.7 Hz, 2H), 7.04 (s, 1H), 6.99 (s, 1H), 4.03 (s, 3H), 4.01 (s, 3H).

##### 2–(3-Fluorophenyl)-3-hydroxy-6,7-dimethoxy-4H-chromen-4-one (19g)

Compound **19 g** was prepared in 80% yield as a yellow powder, following the same procedure as described in the general procedure E with *(E)-3–(3-fluorophenyl)-1–(2-hydroxy-4,5-dimethoxyphenyl)prop-2-en-1-one* (**18 g**) (300 mg, 0.99 mmol), NaOH (3 M aq.) 2 mL and hydrogen peroxide (35% aq., 386 μL, 4 eq) in methanol (10 mL), stirring for 16 h. The reaction mixture was poured into H_2_O and acidified with 3N HCl (pH = 2) and extracted with CH_2_Cl_2_. The crude residue was purified by column chromatography on silica gel (CH_2_Cl_2_/MeOH = 100:0 to 30:1). R_f_ = 0.52 (CH_2_Cl_2_/MeOH = 100:1). ^1^H NMR (600 MHz, CDCl_3_) δ 8.06 (d, *J* = 7.9 Hz, 1H), 7.96 (dd, *J* = 10.5, 1.9 Hz, 1H), 7.54 − 7.46 (m, 2H), 7.19 − 7.07 (m, *J* = 35.4, 20.7, 11.4 Hz, 2H), 7.00 (s, 1H), 4.04 (s, 3H), 4.01 (s, 3H).

##### 2–(3,4-Difluorophenyl)-3-hydroxy-6,7-dimethoxy-4H-chromen-4-one (19h)

Compound **19h** was prepared in 29% yield as a yellow powder, following the same procedure as described in the general procedure E with *(E)-3–(3,4-difluorophenyl)-1–(2-hydroxy-4,5-dimethoxyphenyl)prop-2-en-1-one* (**18h**) (200 mg, 0.99 mmol), NaOH (3 M aq.) 2 mL and hydrogen peroxide (35% aq., 242 μL, 4 eq) in methanol (10 mL), stirring for 16 h. The reaction mixture was poured into H_2_O and acidified with 3N HCl (pH = 2) and extracted with CH_2_Cl_2_. The crude residue was purified by column chromatography on silica gel (CH_2_Cl_2_/MeOH = 100:0 to 30:1). R_f_ = 0.27 (CH_2_Cl_2_/MeOH = 100:1). ^1^H NMR (600 MHz, CDCl_3_) δ 8.14 − 8.04 (m, *J* = 11.9, 7.7, 2.2 Hz, 1H), 8.07 − 7.99 (m, 1H), 7.52 (s, 1H), 7.37 − 7.27 (m, 1H), 7.09 (d, *J* = 2.4 Hz, 1H), 6.99 (s, 1H), 4.04 (s, 3H), 4.01 (s, 3H).

##### 3-Hydroxy-6,7-dimethoxy-2–(4-(trifluoromethyl)phenyl)-4H-chromen-4-one (19i)

Compound **19i** was prepared in 16% yield as yellow powder, following the same procedure as described in the general procedure E with *(E)-1–(2-hydroxy-4,5-dimethoxyphenyl)-3–(4-(trifluoromethyl)phenyl)prop-2-en-1-one* (**18i**) (220 mg, 0.62 mmol), NaOH (3 M aq.) 1.5 mL and hydrogen peroxide (35% aq., 242 μL, 4 eq) in methanol (10 mL), stirring for 16 h. The reaction mixture was poured into H_2_O and acidified with 3N HCl (pH = 2) and extracted with CH_2_Cl_2_. The crude residue was purified by column chromatography on silica gel (CH_2_Cl_2_/MeOH = 100:1 to 30:1). R_f_ = 0.63 (CH_2_Cl_2_/MeOH = 100:1). ^1^H NMR (600 MHz, CDCl_3_) δ 8.36 (d, *J* = 8.3 Hz, 6H), 7.78 (d, *J* = 8.4 Hz, 6H), 7.53 (s, 3H), 7.16 (s, 2H), 7.01 (s, 3H), 4.04 (s, 9H), 4.01 (s, 9H).

##### 3-Hydroxy-6,7-dimethoxy-2–(3-(trifluoromethyl)phenyl)-4H-chromen-4-one (19j)

Compound **19j** was prepared in 8% yield as a yellow powder, following the same procedure as described in the general procedure E with *(E)-1–(2-hydroxy-4,5-dimethoxyphenyl)-3–(3-(trifluoromethyl)phenyl)prop-2-en-1-one* (**18j**) (589 mg, 1.67 mmol), NaOH (3 M aq.) 2 mL and hydrogen peroxide (35% aq., 651 μL, 4 eq) in methanol (10 mL), stirring for 16 h. The reaction mixture was poured into H_2_O and acidified with 3N HCl (pH = 2) and extracted with CH_2_Cl_2_. The crude residue was purified by column chromatography on silica gel (CH_2_Cl_2_/MeOH = 100:1 to 30:1). R_f_ = 0.32 (CH_2_Cl_2_/MeOH = 100:1). ^1^H NMR (600 MHz, CDCl_3_) δ 8.48 (d, *J* = 6.5 Hz, 2H), 7.71 (d, *J* = 7.7 Hz, 1H), 7.66 (t, *J* = 8.1 Hz, 1H), 7.53 (s, 1H), 7.14 (s, 1H), 7.03 (s, 1H), 4.06 (s, 3H), 4.01 (s, 3H).

##### 2–(4-Chlorophenyl)-3-hydroxy-6,7-dimethoxy-4H-chromen-4-one (19k)

Compound **19k** was prepared in 40% yield as a yellow powder, following the same procedure as described in the general procedure E with *(E)-3–(4-chlorophenyl)-1–(2-hydroxy-4,5-dimethoxyphenyl)prop-2-en-1-one* (**18k**) (300 mg, 0.94 mmol), NaOH (3 M aq.) 2 mL and hydrogen peroxide (35% aq., 366 μL, 4 eq) in ethanol (10 mL), stirring for 16 h. The reaction mixture was poured into H_2_O and acidified with 3N HCl (pH = 2) and extracted with CH_2_Cl_2_. The crude residue was purified by column chromatography on silica gel (CH_2_Cl_2_/MeOH = 100:0 to 30:1). R_f_ = 0.3 (CH_2_Cl_2_/MeOH = 100:1). ^1^H NMR (600 MHz, CDCl_3_) δ 8.19 (d, *J* = 8.6 Hz, 2H), 7.57 − 7.46 (m, 3H), 7.08 (s, 1H), 6.99 (s, 1H), 4.03 (s, 3H), 4.01 (s, 3H).

##### 2–(3-Chlorophenyl)-3-hydroxy-6,7-dimethoxy-4H-chromen-4-one (19l)

Compound **19 l** was prepared in 29% yield as a yellow powder, following the same procedure as described in the general procedure E with *(E)-3–(3-chlorophenyl)-1–(2-hydroxy-4,5-dimethoxyphenyl)prop-2-en-1-one* (**18 l**) (300 mg, 0.94 mmol), NaOH (3 M aq.) 2 mL and hydrogen peroxide (35% aq., 288 μL, 4 eq) in methanol (10 mL), stirring for 16 h. The reaction mixture was poured into H_2_O and acidified with 3N HCl (pH = 2) and extracted with CH_2_Cl_2_. The crude residue was purified by column chromatography on silica gel (CH_2_Cl_2_/MeOH = 100:0 to 30:1). R_f_ = 0.35 (CH_2_Cl_2_/MeOH = 100:1). ^1^H NMR (600 MHz, CDCl_3_) δ 8.21 (t, *J* = 1.7 Hz, 1H), 8.17 (d, *J* = 7.8 Hz, 1H), 7.52 (s, 1H), 7.50 − 7.39 (m, 2H), 7.09 (d, *J* = 11.1 Hz, 1H), 7.01 (s, 1H), 4.04 (s, 3H), 4.01 (s, 3H). HRMS *m*/*z* calculated for C_17_H_13_ClO_5_ [M + H]^+^: 333.0525; found: 333.0525.

##### 2-([1,1'-Biphenyl]-4-yl)-3-hydroxy-6,7-dimethoxy-4H-chromen-4-one (19m)

Compound **19 m** was prepared in 72% yield as a yellow powder, following the same procedure as described in the general procedure E with *(E)-3-([1,1′-biphenyl]-4-yl)-1–(2-hydroxy-4,5-dimethoxyphenyl)prop-2-en-1-one* (**18 m**) (400 mg, 1.11 mmol), NaOH (3 M aq.) 2 mL and hydrogen peroxide (35% aq., 431 μL, 4 eq) in methanol (20 mL), stirring for 16 h. The reaction mixture was poured into H_2_O and acidified with 3N HCl (pH = 2) and extracted with CH_2_Cl_2_. The crude residue was purified by column chromatography on silica gel (CH_2_Cl_2_/MeOH = 100:0 to 30:1). R_f_ = 0.29 (CH_2_Cl_2_/MeOH = 30:1). ^1^H NMR (600 MHz, CDCl_3_) δ 8.32 (d, *J* = 8.2 Hz, 2H), 7.77 (d, *J* = 8.2 Hz, 2H), 7.68 (d, *J* = 7.7 Hz, 2H), 7.64 (d, *J* = 3.8 Hz, 1H), 7.55 (s, 1H), 7.48 (d, *J* = 7.4 Hz, 2H), 7.40 (t, *J* = 6.3 Hz, 1H), 7.11 (s, 1H), 7.03 (s, 1H), 4.04 (s, 3H), 4.02 (s, 3H). HRMS *m*/*z* calculated for C_23_H_18_O_5_ [M + H]^+^: 375.1227; found: 375.1240.

##### 3-Hydroxy-6,7-dimethoxy-2–(4-phenoxyphenyl)-4H-chromen-4-one (19n)

Compound **19n** was prepared in 16% yield as a yellow powder, following the same procedure as described in the general procedure E with *(E)-1–(2-hydroxy-4,5-dimethoxyphenyl)-3–(4-phenoxyphenyl)prop-2-en-1-one* (**18n**) (500 mg, 0.69 mmol), NaOH (3 M aq.) 2 mL and hydrogen peroxide (35% aq., 516 μL, 4 eq) in methanol (10 mL), stirring for 16 h. The reaction mixture was poured into H_2_O and acidified with 3N HCl (pH = 2) and extracted with CH_2_Cl_2_. The crude residue was purified by column chromatography on silica gel (CH_2_Cl_2_/MeOH = 100:0 to 30:1). R_f_ = 0.29 (CH_2_Cl_2_/MeOH = 30:1). ^1^H NMR (600 MHz, CDCl_3_) δ 8.21 (d, *J* = 8.8 Hz, 2H), 7.53 (s, 1H), 7.39 (t, *J* = 7.9 Hz, 2H), 7.18 (t, *J* = 7.4 Hz, 1H), 7.13 (d, *J* = 8.8 Hz, 2H), 7.10 (d, *J* = 8.2 Hz, 2H), 6.98 (s, 2H), 4.02 (s, 3H), 4.01 (s, 3H). HRMS *m*/*z* calculated for C_23_H_18_O_6_ [M + H]^+^: 391.1176; found: 391.1181.

##### 3-Hydroxy-6,7-dimethoxy-2–(3-phenoxyphenyl)-4H-chromen-4-one (19o)

Compound **19o** was prepared in 32% yield as a yellow powder, following the same procedure as described in the general procedure E with *(E)-1–(2-hydroxy-4,5-dimethoxyphenyl)-3–(3-phenoxyphenyl)prop-2-en-1-one* (**18o**) (800 mg, 0.69 mmol), NaOH (3 M aq.) 4 mL and hydrogen peroxide (35% aq., 990 μL, 4 eq) in methanol (20 mL), stirring for 16 h. The reaction mixture was poured into H_2_O and acidified with 3N HCl (pH = 2) and extracted with CH_2_Cl_2_. The crude residue was purified by column chromatography on silica gel (CH_2_Cl_2_/MeOH = 100:0 to 30:1). R_f_ = 0.33 (CH_2_Cl_2_/MeOH = 30:1). ^1^H NMR (600 MHz, CDCl_3_) δ 8.02 (d, *J* = 7.9 Hz, 1H), 7.93 (s, 1H), 7.51 (s, 1H), 7.49 (t, *J* = 8.0 Hz, 1H), 7.37 (t, *J* = 7.9 Hz, 2H), 7.14 (t, *J* = 7.3 Hz, 1H), 7.10 − 7.01 (m, 4H), 6.97 (s, 1H), 4.01 (s, 3H), 4.00 (s, 3H). HRMS *m*/*z* calculated for C_23_H_18_O_6_ [M + H]^+^: 391.1176; found: 391.1191.

##### 3-Hydroxy-6,7-dimethoxy-2–(4-methoxyphenyl)-4H-chromen-4-one (19p)

Compound **19p** was prepared in 58% yield as a yellow powder, following the same procedure as described in the general procedure E with *(E)-1–(2-hydroxy-4,5-dimethoxyphenyl)-3–(4-methoxyphenyl)prop-2-en-1-one* (**18p**) (300 mg, 0.95 mmol), NaOH (3 M aq.) 2 mL and hydrogen peroxide (35% aq., 371 μL, 4 eq) in methanol (10 mL), stirring for 16 h. The reaction mixture was poured into H_2_O and acidified with 3N HCl (pH = 2) and extracted with CH_2_Cl_2_. The crude residue was purified by column chromatography on silica gel (CH_2_Cl_2_/MeOH = 100:1 to 8:1). R_f_ = 0.21 (CH_2_Cl_2_/MeOH = 30:1). ^1^H NMR (600 MHz, CDCl_3_) δ 8.20 (d, *J* = 9.0 Hz, 2H), 7.52 (d, *J* = 5.5 Hz, 1H), 7.05 (d, *J* = 9.0 Hz, 2H), 6.99 (s, 2H), 4.02 (s, 3H), 4.00 (s, 3H), 3.90 (s, 3H).

##### 3-Hydroxy-6,7-dimethoxy-2–(3-methoxyphenyl)-4H-chromen-4-one (19q)

Compound **19q** was prepared in 43% yield as a yellow powder, following the same procedure as described in the general procedure E with *(E)-1–(2-hydroxy-4,5-dimethoxyphenyl)-3–(3-methoxyphenyl)prop-2-en-1-one* (**18q**) (300 mg, 0.95 mmol), NaOH (3 M aq.) 2 mL and hydrogen peroxide (35% aq., 371 μL, 4 eq) in methanol (10 mL), stirring for 16 h. The reaction mixture was poured into H_2_O and acidified with 3N HCl (pH = 2) and extracted with CH_2_Cl_2_. The crude residue was purified by column chromatography on silica gel (CH_2_Cl_2_/MeOH = 100:1 to 10:1). R_f_ = 0.16 (CH_2_Cl_2_/MeOH = 30:1). ^1^H NMR (600 MHz, CDCl_3_) δ 7.88 − 7.77 (m, 2H), 7.53 (s, 1H), 7.45 (t, *J* = 8.1 Hz, 1H), 7.10 − 6.95 (m, 3H), 4.03 (s, 3H), 4.01 (s, 3H), 3.91 (s, 3H).

##### Methyl 4–(3-hydroxy-6,7-dimethoxy-4-oxo-4H-chromen-2-yl)benzoate (19r)

Compound **19r** was prepared in 17% yield as a yellow powder, following the same procedure as described in the general procedure E with *(E)-methyl 4–(3-(2-hydroxy-4,5-dimethoxyphenyl)-3-oxoprop-1-en-1-yl)benzoate* (**18r**) (800 mg, 0.68 mmol), NaOCH_3_ (5.4 M in methanol solution, 1.7 mL, 4 eq) and hydrogen peroxide (35% aq., 908 μL, 4 eq) in methanol (30 mL), stirring for 12 h. The reaction mixture was poured into H_2_O and acidified with 3N HCl (pH = 2) and extracted with CH_2_Cl_2_. The crude residue was purified by column chromatography on silica gel (CH_2_Cl_2_/MeOH = 50:0 to 10:1). R_f_ = 0.44 (CH_2_Cl_2_/MeOH = 10:1). ^1^H NMR (600 MHz, CDCl_3_) δ 8.33 (d, *J* = 8.6 Hz, 2H), 8.18 (d, *J* = 8.6 Hz, 2H), 7.53 (s, 1H), 7.18 (s, 1H), 7.02 (s, 1H), 4.04 (s, 3H), 4.01 (s, 3H), 3.97 (s, 3H). HRMS *m*/*z* calculated for C_19_H_16_O_7_ [M + H]^+^: 357.0969; found: 357.0976.

##### Methyl 3–(3-hydroxy-6,7-dimethoxy-4-oxo-4H-chromen-2-yl)benzoate (19s)

Compound **19s** was prepared in 18% yield as a yellow powder, following the same procedure as described in the general procedure E with *(E)-methyl 3–(3-(2-hydroxy-4,5-dimethoxyphenyl)-3-oxoprop-1-en-1-yl)benzoate* (**18s**) (330 mg, 0.96 mmol), NaOCH_3_ (5.4 M in methanol solution, 0.7 mL, 4 eq) and hydrogen peroxide (35% aq., 374 μL, 4 eq) in methanol (10 mL), stirring for 12 h. The reaction mixture was poured into H_2_O and acidified with 3N HCl (pH = 2) and extracted with CH_2_Cl_2_. The crude residue was purified by column chromatography on silica gel (CH_2_Cl_2_/MeOH = 100:1 to 8:1). R_f_ = 0.43 (CH_2_Cl_2_/MeOH = 10:1). ^1^H NMR (600 MHz, CDCl_3_) δ 8.84 (s, 1H), 8.48 (d, *J* = 6.8 Hz, 1H), 8.10 (d, *J* = 7.4 Hz, 1H), 7.60 (t, *J* = 7.5 Hz, 1H), 7.51 (s, 1H), 7.04 (s, 1H), 4.04 (s, 3H), 4.00 (s, 3H), 3.97 (d, *J* = 10.6 Hz, 3H). HRMS *m*/*z* calculated for C_19_H_16_O_7_ [M + H]^+^: 357.0969; found: 357.0969.

##### 3-Hydroxy-6,7-dimethoxy-2-(naphthalen-2-yl)-4H-chromen-4-one (22a)

Compound **22a** was prepared in 15% yield as a yellow powder, following the same procedure as described in the general procedure E with *(E)-1–(2-hydroxy-4,5-dimethoxyphenyl)-3-(naphthalen-2-yl)prop-2-en-1-one* (**21a**) (230 mg, 0.69 mmol), NaOH (3 M aq.) 1.5 mL and hydrogen peroxide (35% aq., 267 μL, 4 eq) in ethanol (10 mL), stirring for 15 h. The reaction mixture was poured into H_2_O and acidified with 3N HCl (pH = 2) and extracted with CH_2_Cl_2_. The crude residue was purified by column chromatography on silica gel (CH_2_Cl_2_/MeOH = 100:0 to 30:1). R_f_ = 0.58 (CH_2_Cl_2_/MeOH = 30:1). ^1^H NMR (600 MHz, CDCl_3_) δ 8.78 (s, 1H), 8.32 (dd, *J* = 8.7, 1.7 Hz, 1H), 7.99 (dd, *J* = 11.3, 7.9 Hz, 2H), 7.93 − 7.86 (m, 1H), 7.62 − 7.52 (m, 3H), 7.14 (s, 1H), 7.08 (d, *J* = 6.2 Hz, 1H), 4.06 (s, 3H), 4.02 (s, 3H). HRMS *m*/*z* calculated for C_21_H_16_O_5_ [M + H]^+^: 349.1071; found: 349.1077.

##### 3-Hydroxy-6,7-dimethoxy-2–(1-methyl-1H-pyrazol-4-yl)-4H-chromen-4-one (22b)

Compound **22b** was prepared in 46% yield as a yellow powder, following the same procedure as described in the general procedure E with *(E)-1–(2-hydroxy-4,5-dimethoxyphenyl)-3–(1-methyl-1H-pyrazol-4-yl)prop-2-en-1-one* (**21b**) (338 mg, 1.35 mmol), NaOH (3 M aq.) 2 mL and hydrogen peroxide (35% aq., 523 μL, 4 eq) in ethanol (10 mL), stirring for 15 h. The reaction mixture was poured into H_2_O and acidified with 3N HCl (pH = 2) and extracted with CH_2_Cl_2_. The crude residue was purified by column chromatography on silica gel (CH_2_Cl_2_/MeOH = 100:0 to 30:1). R_f_ = 0.39 (CH_2_Cl_2_/MeOH = 30:1). ^1^H NMR (600 MHz, CDCl_3_) δ 8.15 (s, 1H), 8.11 (s, 1H), 7.52 (s, 1H), 6.69 (s, 1H), 4.03 (s, 3H), 4.02 (s, 3H), 3.99 (s, 3H). HRMS *m*/*z* calculated for C_15_H_14_N_2_O_5_ [M + H]^+^: 303.0976; found: 303.0971.

##### 3-Hydroxy-6,7-dimethoxy-2-(thiophen-2-yl)-4H-chromen-4-one (22c)

Compound **22c** was prepared in 22% yield as a yellow powder, following the same procedure as described in the general procedure F with *(E)-1–(2-hydroxy-4,5-dimethoxyphenyl)-3-(thiophen-2-yl)prop-2-en-1-one* (**21c**) (273 mg, 0.94 mmol), NaOH (3 M aq.) 1.5 mL and hydrogen peroxide (35% aq., 365 μL, 4 eq) in ethanol (10 mL), stirring for 15 h. The reaction mixture was poured into H_2_O and acidified with 3N HCl (pH = 2) and extracted with CH_2_Cl_2_. The crude residue was purified by column chromatography on silica gel (CH_2_Cl_2_/MeOH = 100:0 to 30:1). R_f_ = 0.58 (CH_2_Cl_2_/MeOH = 30:1). ^1^H NMR (600 MHz, CDCl_3_) δ 7.98 − 7.90 (m, 1H), 7.59 (dd, *J* = 4.8 Hz and 1.2 Hz, 1H), 7.52 (s, 1H), 7.24 − 7.22 (m, 1H), 6.98 (s, 1H), 4.03 (s, 3H), 3.99 (s, 3H). HRMS *m*/*z* calculated for C_15_H_12_O_5_S [M + H]^+^: 305.0477; found: 305.0469.

### In silico *docking studies*

*Ligand preparation and optimisation:* All ligands were generated as 2D and 3D structure by *ChemDraw Ultra* (ver. 12.0.2) and *Chem3D Pro* (ver. 11.0.1), respectively. Ligand preparation and optimisation was followed by *“Sanitize”* preparation protocol in *SYBYL-X 2.1.1* (Tripos Inc., St Louis) to clean up of the structures involving filling valences, standardising, removing duplicates and producing only one molecule per input structure. The group of ligands was saved as *.sdf* file.

*Protein preparation:* For IP6K2, the protein model of human IP6K2 (UniProt: Q9UHH9) was downloaded from AlphaFold2 structure database (https://alphafold.ebi.ac.uk) as PDB and FASTA format. *SYBYL-X 2.1.1* program was employed for protein preparation including conflicted side chains of amino acid residues fixation. Hydrogen atoms were added under the application of *AMBER7 FF99* Force Field setting for IP6K2, respectively. Minimisation process was performed by *POWELL* method, and initial optimisation option was set to N*one* for IP6K2. Termination gradient and max iteration were set 0.05 kcal/(mol*Å) and 100 times.

*Docking and scoring function studies:* The docking studies of all prepared ligands were performed by *Surflex-Dock GeomX* module in *SYBYL-X 2.1.1*. For CYP3A4, docking was guided by the *Surflex-Dock protomol* and docking site was defined by the “*Ligand*” method with the reported ligand quercetin. *Surflex-Dock protomol* set to “*Residues*” method with selected amino acids (Lys42, Leu206, Glu207, Asn208, Leu209, Thr210, Val 218, Leu219, Asp220, Leu221, Lys222, Asp383; radius setting: 2.2; Those amino acids were selected based on the active site of *Eh*IP6KA.) was used to guide docking site for IP6K2 homology model. Two factors related with a generation of Protomol are *Bloat(Å)* and *Threshold* were set to 0.5 and 0, respectively. Other parameters were applied with its default settings in all runs.

### ADP-Glo kinase assay

Inhibition activity of the synthesised compounds against IP6K2 was assessed using ADP-Glo™ Kinase Assay (Promega). Kinase reaction mixtures were first shaking-incubated with individual drugs (diluted in DMSO) for pre-incubation at RT for 15 min, and then ATP was added (final 10 µM) to initiate the reaction. 20 ng of recombinant human IP6K2 protein was used per 15 µL of IP6K2 reaction mixture (50 mM Tris-HCl, pH 6.8, 10 mM MgCl_2_, 2.5 mM DTT, 0.02% Triton X-100, 10 µM IP6). Kinase reaction was proceeded at 37 °C for 30 min with 100 rpm shaking, and the amount of ADP produced was measured on white plates using Mithras LB940 plate reader (Berthold) according to the manufacturer’s protocol. Each detection set was prepared on 96-well (25 µL kinase reaction) as duplicates. Resulting enzyme activity was calculated as following; Activity (%) = ([ADP_experimental_]/[ADP_DMSO_])×100. For IC_50_ estimation of compounds, kinase assay was prepared on 384-well plates (20 ng of recombinant human IP6K2 protein was used per 5 µL kinase reaction mixture) as duplicates. The final concentrations for IP6K2, IP6, and ATP were 80 nM, 10 μM, and 10 μM, respectively. Kinase reaction was proceeded at 37 °C for 40 min with 300 rpm shaking, and the amount of ADP produced was measured using Synergy Neo microplate reader (Biotek) according to the manufacturer’s protocol. IP6K1 and IP6K3 assays were performed by Liao *et al*.’s conditions.^25^ The final concentrations for IP6K1, IP6, and ATP were 60 nM, 100 μM, and 1 mM, respectively. The final concentrations for IP6K3, IP6, and ATP were 120 nM, 100 μM, and 1 mM, respectively. The mixed reaction plate was placed into a 37 °C incubator and rotated on an orbital shaker at 300 rpm for 30 min. All statistical analyses including IC_50_ estimation was performed using Prism 7 or Prism 9 (Graphpad Software).

### Recombinant IP6K enzyme purification

Full-length human IP6K1, IP6K2, or IP6K3 cDNA was subcloned into pFASTBAC1 plasmid (Gibco) with an N-terminal FLAG epitope sequence, and baculovirus was generated according to the manufacturer’s instructions. Sf9 insect cells were infected with baculovirus and incubated for 72 h. The cells were resuspended in lysis buffer [20 mM Tris–HCl (pH 7.9), 500 mM NaCl, 4 mM MgCl_2_, 0.4 mM EDTA, 2 mM DTT, 20% glycerol, 1 mM PMSF, and protease inhibitor cocktail (Roche)] and then disrupted with a Dounce homogeniser (pestle A, 3 series of 10 strokes with 10 min interval). Clarified extracts were adjusted to 300 mM NaCl by adding dilution buffer [20 mM Tris–HCl (pH 7.9) and 10% glycerol], supplemented with final 0.1% NP-40, and then subjected to affinity purification on M2 agarose (Sigma). After extensive washing with wash buffer [20 mM Tris–HCl (pH 7.9), 150 mM NaCl, 2 mM MgCl_2_, 0.2 mM EDTA, 1 mM DTT, 15% glycerol, 1 mM PMSF, and 0.1% NP-40], FLAG-IP6K proteins were eluted with elution buffer (wash buffer containing 0.25 mg/mL FLAG-peptide and protease inhibitor cocktail) and stored at −80 °C (See Supporting Information Figure 5).

### Extractions and quantification of intracellular inositol phosphates by HPLC

For HPLC analysis, 2 × 10^5^ cells/60 mm dish of HCT116 were treated with 60 μCi [^3^H] myo-inositol (NET1177001MC, PerkinElmer). After 3 days, soluble inositol polyphosphates from HCT116 (human colorectal cancer cell line) were extracted and analysed as previously described.^39^ Intracellular inositol phosphates were extracted with acid extraction buffer (1M HClO_4_, 3 mM EDTA, and 0.1 mg/mL IP6), and neutralised with neutralisation buffer (1M K_2_CO_3_ and 3 mM EDTA). The lysates were centrifuged for 10 min, and the soluble fraction was resolved by HPLC as described earlier.^39^ The lipid pellet was lysed with 0.1% Triton X-100 in NaOH overnight. Each fraction was mixed with Ultima-Flo AP liquid scintillation cocktail (6013599, PerkinElmer), and radioactivity was counted in a scintillation counter. Quantity of inositol phosphates was presented as total counts/min (CPM) normalised by total lipid contents.

## Supplementary Material

Supplemental Material
