# Supplementary material for: Synthesis and biological evaluation of flavonoid-based IP6K2 inhibitors
Source: J Enzyme Inhib Med Chem. 2023 Apr 4;38(1):2193866. doi: 10.1080/14756366.2023.2193866 (PMC10075506; doi:10.1080/14756366.2023.2193866)
Supplement: Supplemental Material [file IENZ_A_2193866_SM7463.pdf]

Supporting Information for

Synthesis and Biological Evaluation of Flavonoid-based IP6K2 Inhibitors

Myunghwan Ahn[a,#], Seung Eun Park[b,#], Jiyeon Choi[b,#], Jiahn Choi[a], Doyoung Choi[a], Dongju An[b], Hayoung Jeon[a], Soowhan Oh[a], Kiho Lee[a], Jaehoon Kim[b], Jaebong Jang[a,*], Seyun Kim[b,c,*] and Youngjoo Byun[a,d,*]

[a] *College of Pharmacy, Korea University, 2511 Sejong-ro, Sejong 30019, Republic of Korea*

[b] *Department of Biological Sciences, Korea Advanced Institute of Science and Technology (KAIST), Daejeon 344, Republic of Korea*

[c] *KAIST Institute for the BioCentury and KAIST Stem Cell Center, KAIST, Daejeon, Republic of Korea.*

[d] *Institute of Pharmaceutical Science and Translational Research, 2511 Sejong-ro, Sejong 30019, Korea, University, Sejong 30019, Republic of Korea*

[#] *Equal contribution*

* Corresponding authors; jaebong_jang@korea.ac.kr, seyunkim@kaist.ac.kr, yjbyun1@korea.ac.kr

Table of contents

Content	page
^{1}H NMR spectra of compound **20a**	S1
HRMS chromatogram of compound **20a**	S1
^{1}H NMR spectra of compound **20b**	S2
^{13}C NMR spectra of compound **20b**	S2
HRMS chromatogram of compound **20b**	S3
HPLC chromatogram of compound **20b** eluted using ACN/Water	S3
^{1}H NMR spectra of compound **20c**	S4
^{13}C NMR spectra of compound **20c**	S4
HRMS chromatogram of compound **20c**	S5
HPLC chromatogram of compound **20c** eluted using ACN/Water	S5
^{1}H NMR spectra of compound **20d**	S6
^{13}C NMR spectra of compound **20d**	S6
HRMS chromatogram of compound **20d**	S7
HPLC chromatogram of compound **20d** eluted using ACN/Water	S7
^{1}H NMR spectra of compound **20e**	S8
^{13}C NMR spectra of compound **20e**	S8
HRMS chromatogram of compound **20e**	S9
HPLC chromatogram of compound **20e** eluted using ACN/Water	S9
^{1}H NMR spectra of compound **20f**	S10
^{13}C NMR spectra of compound **20f**	S10
HRMS chromatogram of compound **20f**	S11
HPLC chromatogram of compound **20f** eluted using ACN/Water	S11

Table of contents

Content	page
^{1}H NMR spectra of compound **20g**	S12
^{13}C NMR spectra of compound **20g**	S12
HRMS chromatogram of compound **20g**	S13
HPLC chromatogram of compound **20g** eluted using ACN/Water	S13
^{1}H NMR spectra of compound **20h**	S14
^{13}C NMR spectra of compound **20h**	S14
HRMS chromatogram of compound **20h**	S15
HPLC chromatogram of compound **20h** eluted using ACN/Water	S15
^{1}H NMR spectra of compound **20i**	S16
^{13}C NMR spectra of compound **20i**	S16
HRMS chromatogram of compound **20i**	S17
HPLC chromatogram of compound **20i** eluted using ACN/Water	S17
^{1}H NMR spectra of compound **20j**	S18
^{13}C NMR spectra of compound **20j**	S18
HRMS chromatogram of compound **20j**	S19
HPLC chromatogram of compound **20j** eluted using ACN/Water	S19
^{1}H NMR spectra of compound **20k**	S20
^{13}C NMR spectra of compound **20k**	S20
HRMS chromatogram of compound **20k**	S21
HPLC chromatogram of compound **20k** eluted using ACN/Water	S21
^{1}H NMR spectra of compound **20l**	S22
^{13}C NMR spectra of compound **20l**	S22
HRMS chromatogram of compound **20l**	S23
HPLC chromatogram of compound **20l** eluted using ACN/Water	S23

Table of contents

Content	page
^{1}H NMR spectra of compound **20m**	S24
^{13}C NMR spectra of compound **20m**	S24
HRMS chromatogram of compound **20m**	S25
HPLC chromatogram of compound **20m** eluted using ACN/Water	S25
^{1}H NMR spectra of compound **20n**	S26
^{13}C NMR spectra of compound **20n**	S26
HRMS chromatogram of compound **20n**	S27
HPLC chromatogram of compound **20n** eluted using ACN/Water	S27
^{1}H NMR spectra of compound **20o**	S28
^{13}C NMR spectra of compound **20o**	S28
HRMS chromatogram of compound **20o**	S29
HPLC chromatogram of compound **20o** eluted using ACN/Water	S29
^{1}H NMR spectra of compound **20p**	S30
HRMS chromatogram of compound **20p**	S30
^{1}H NMR spectra of compound **20q**	S31
HRMS chromatogram of compound **20q**	S31
^{1}H NMR spectra of compound **20r**	S32
^{13}C NMR spectra of compound **20r**	S32
HRMS chromatogram of compound **20r**	S33
HPLC chromatogram of compound **20r** eluted using ACN/Water	S33
^{1}H NMR spectra of compound **20s**	S34
^{13}C NMR spectra of compound **20s**	S34
HRMS chromatogram of compound **20s**	S35
HPLC chromatogram of compound **20s** eluted using ACN/Water	S35

Table of contents

Content	page
^{1}H NMR spectra of compound **23a**	S36
^{13}C NMR spectra of compound **23a**	S36
HRMS chromatogram of compound **23a**	S37
HPLC chromatogram of compound **23a** eluted using ACN/Water	S37
^{1}H NMR spectra of compound **23b**	S38
^{13}C NMR spectra of compound **23b**	S38
HRMS chromatogram of compound **23b**	S39
HPLC chromatogram of compound **23b** eluted using ACN/Water	S39
^{1}H NMR spectra of compound **23c**	S40
^{13}C NMR spectra of compound **23c**	S40
HRMS chromatogram of compound **23c**	S41
HPLC chromatogram of compound **23c** eluted using ACN/Water	S41
**Supporting Information Figure 1.** IP6K2 inhibition of the synthesized compounds **9a-10i**	S42
**Supporting Information Figure 2.** Chemical structures of compounds **9a-10i**	S42
**Supporting Information Figure 3.** IP6K1 and IP6K3 inhibition of **20s** and quercetin	S43
**Supporting information Figure 4**. Inhibition rate of compound **20s** against IP6K1, IP6K2 and IP6K3 with the addition of ATP and IP6 at final concentration of 10 μM	S43
**Supporting Information Figure 5**. The cellular effects of **20s** on IP7 synthesis in the HCT116 cell line	S43
**Supporting Information Figure 6**. Purification of recombinant human IP6K proteins.	S44
**Supporting Information Figure 7.** Aggregation test of 20s and quercetin with IP6K2	S44
**Supporting Information Table 1.** PAMPA permeability of compound **20s** and quercetin	S45

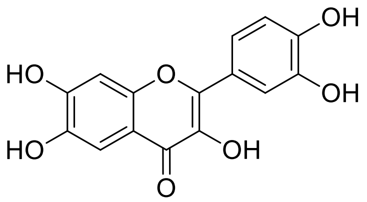

20a

^{1}H NMR spectra of compound **20a** measured in DMSO at 600 MHz

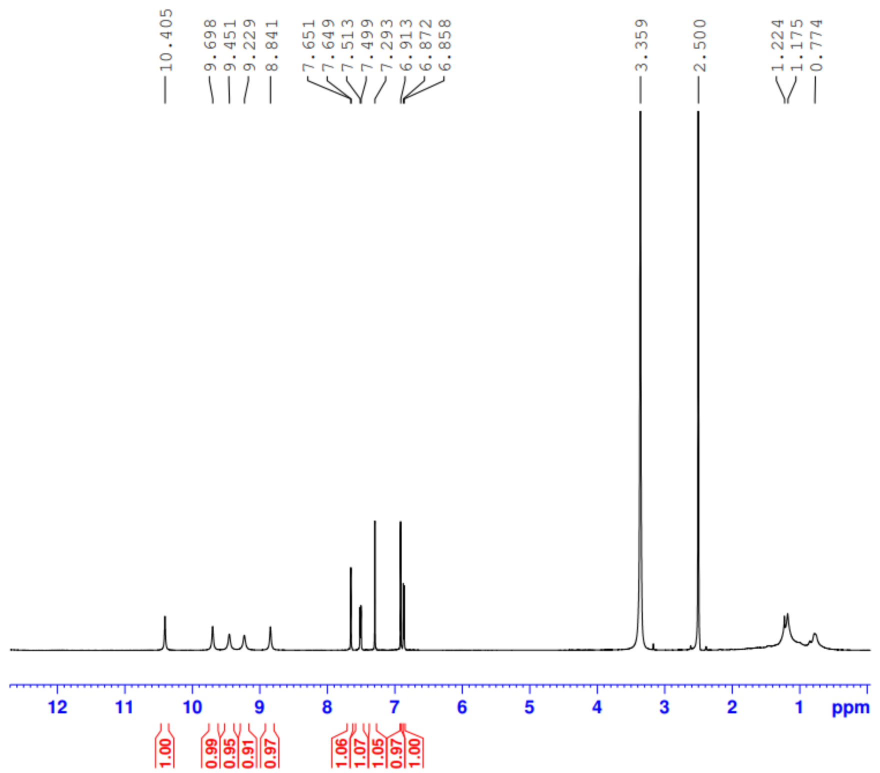

HRMS chromatogram of compound **20a**

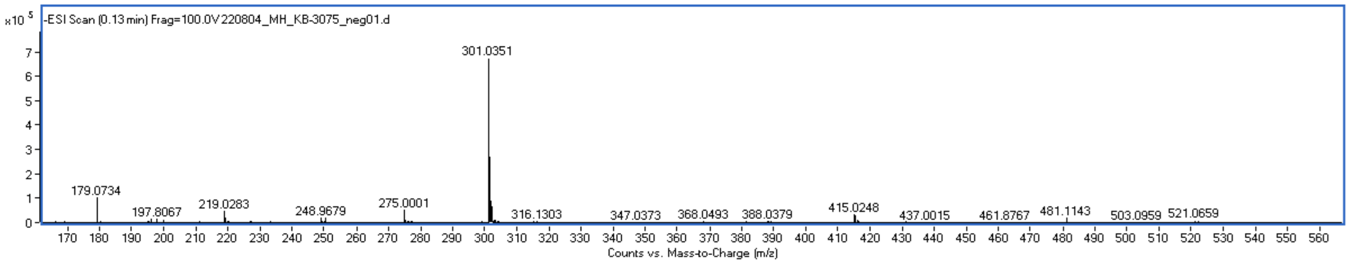

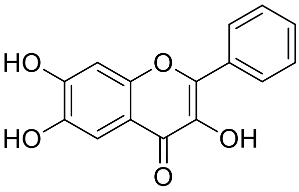

20b

^{1}H NMR spectra of compound **20b** measured in DMSO at 600 MHz

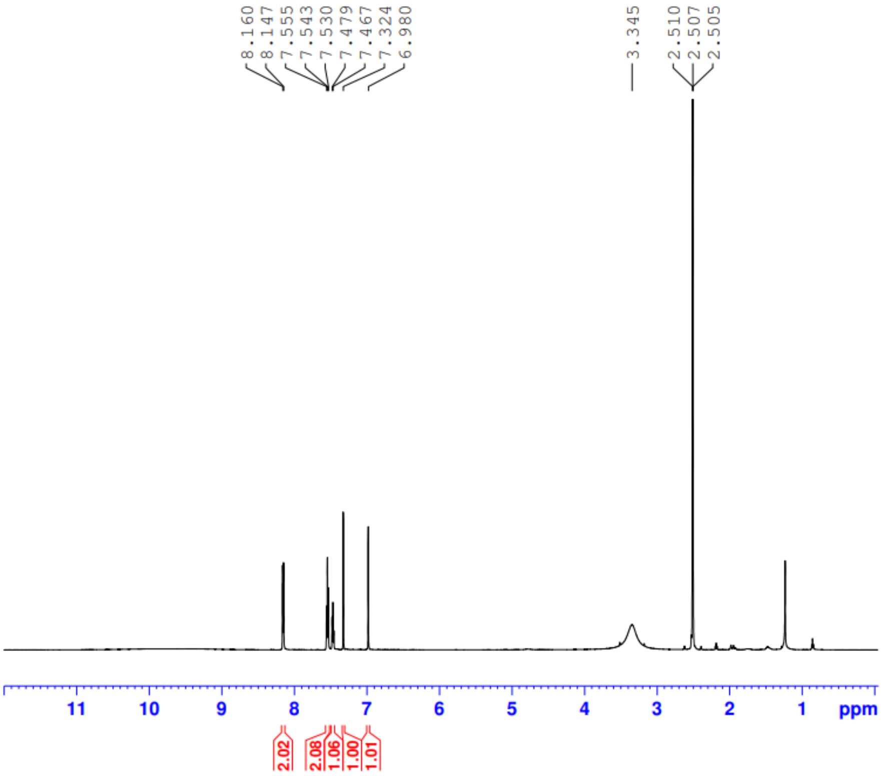

^{13}C NMR spectra of compound **20b** measured in DMSO at 600 MHz

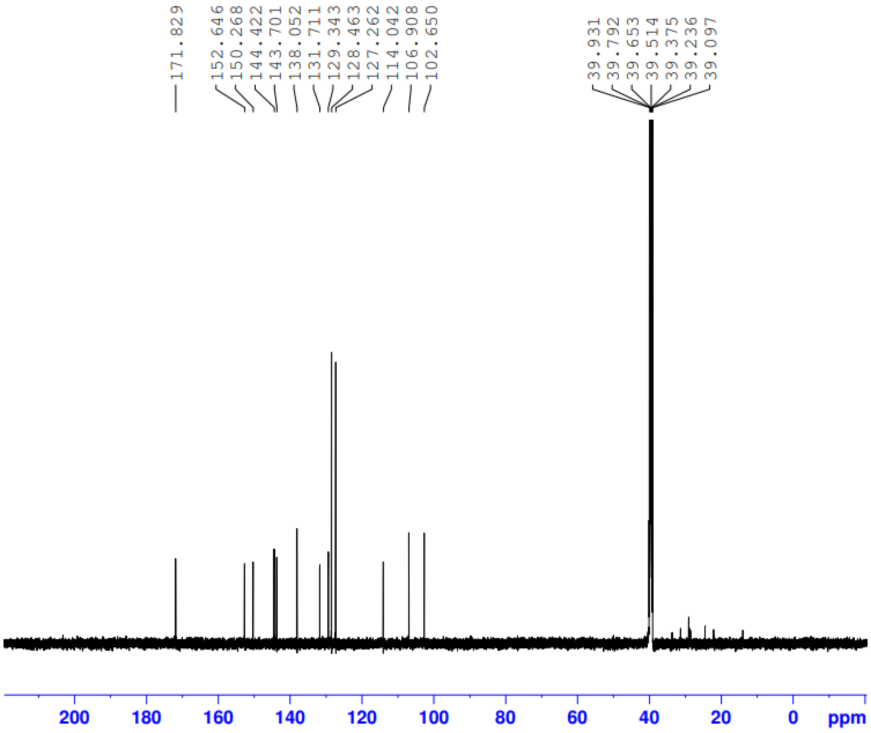

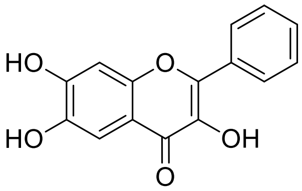

20b

HRMS chromatogram of compound **20b**

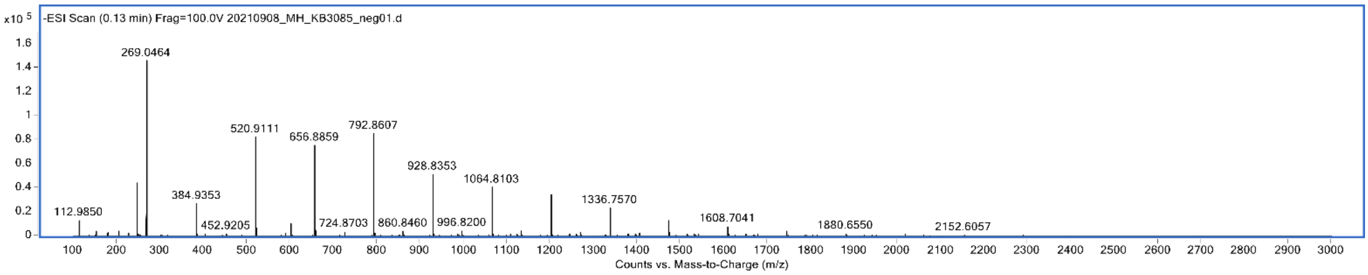

HPLC chromatogram of compound **20b** eluted using ACN/Water

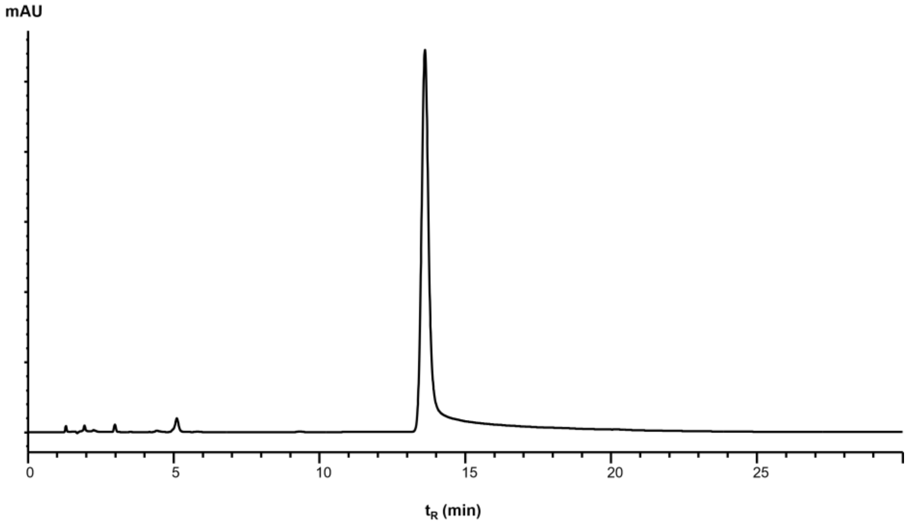

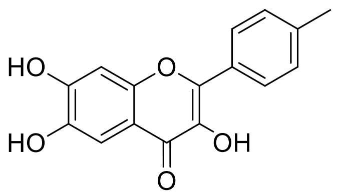

20c

^{1}H NMR spectra of compound **20c** measured in MeOD at 600 MHz

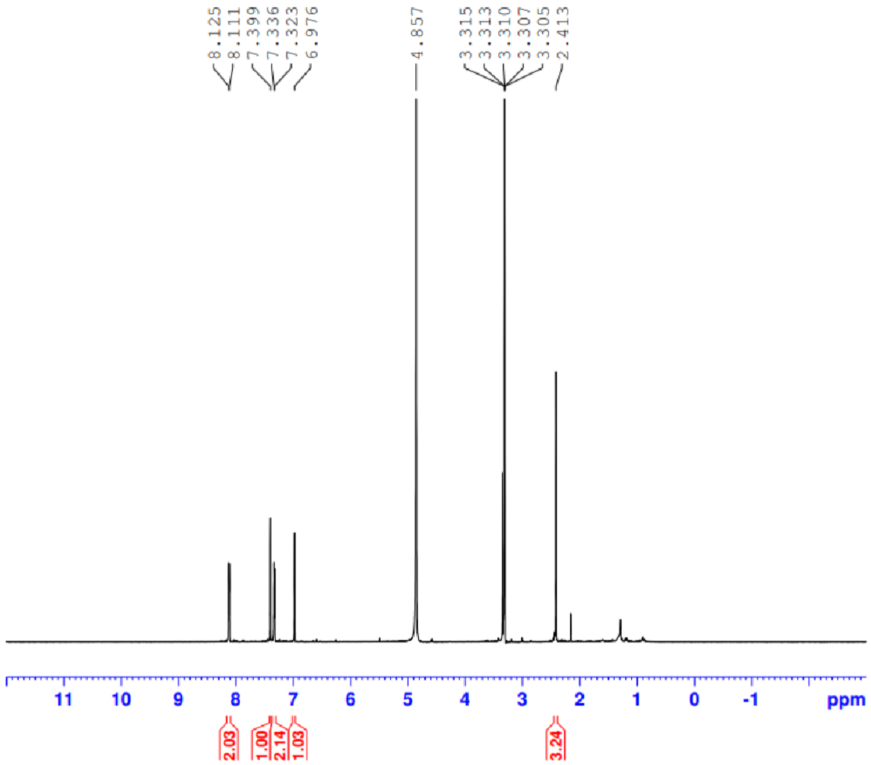

^{13}C NMR spectra of compound **20c** measured in DMSO at 600 MHz

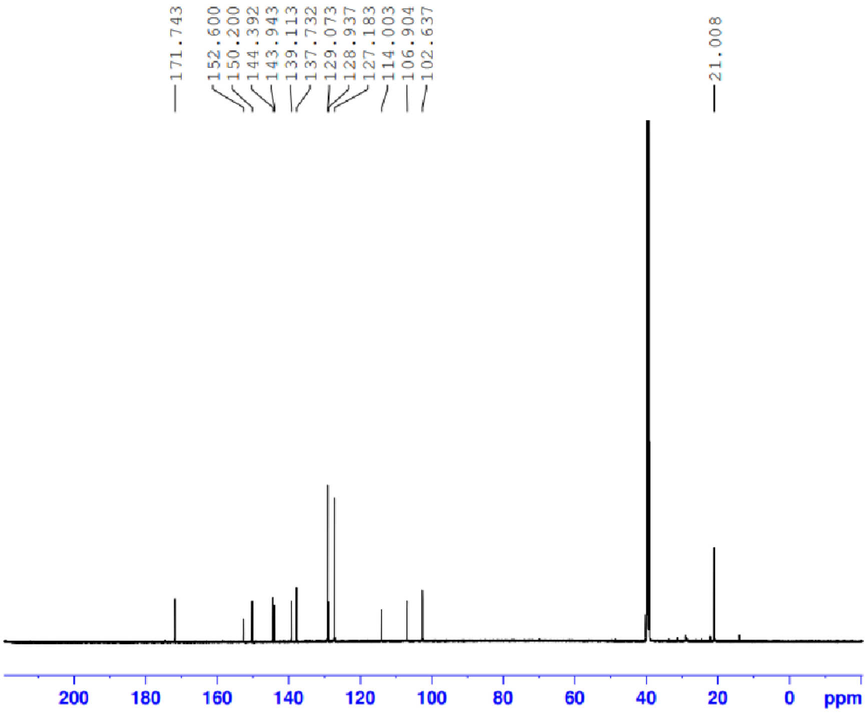

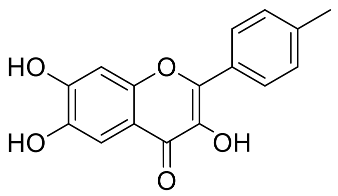

20c

HRMS chromatogram of compound **20c**

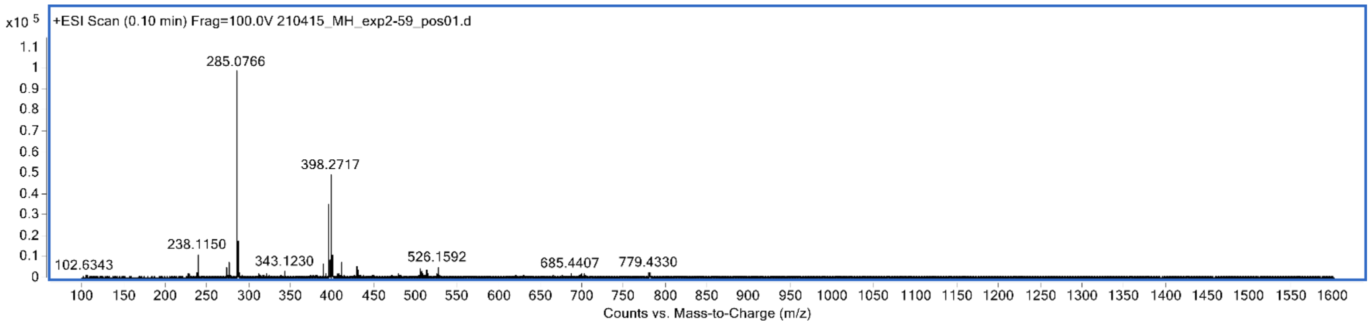

HPLC chromatogram of compound **20c** eluted using ACN/Water

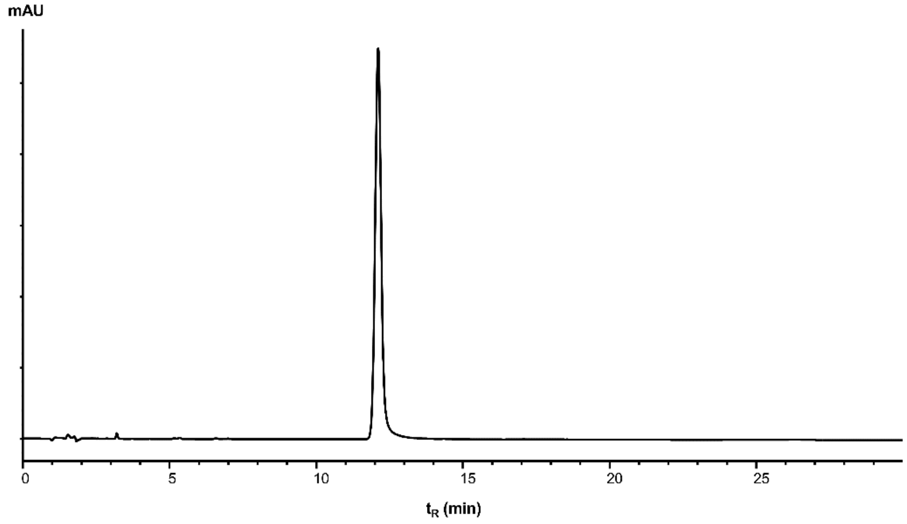

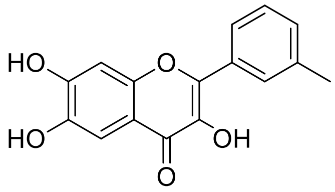

20d

^{1}H NMR spectra of compound **20d** measured in MeOD at 600 MHz

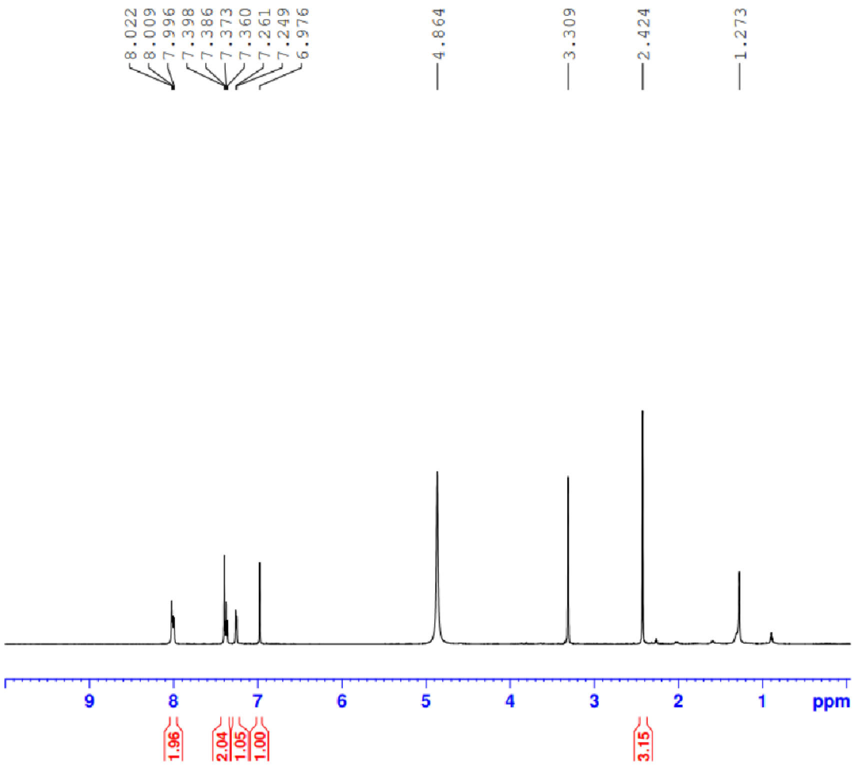

^{13}C NMR spectra of compound **20d** measured in MeOD at 600 MHz

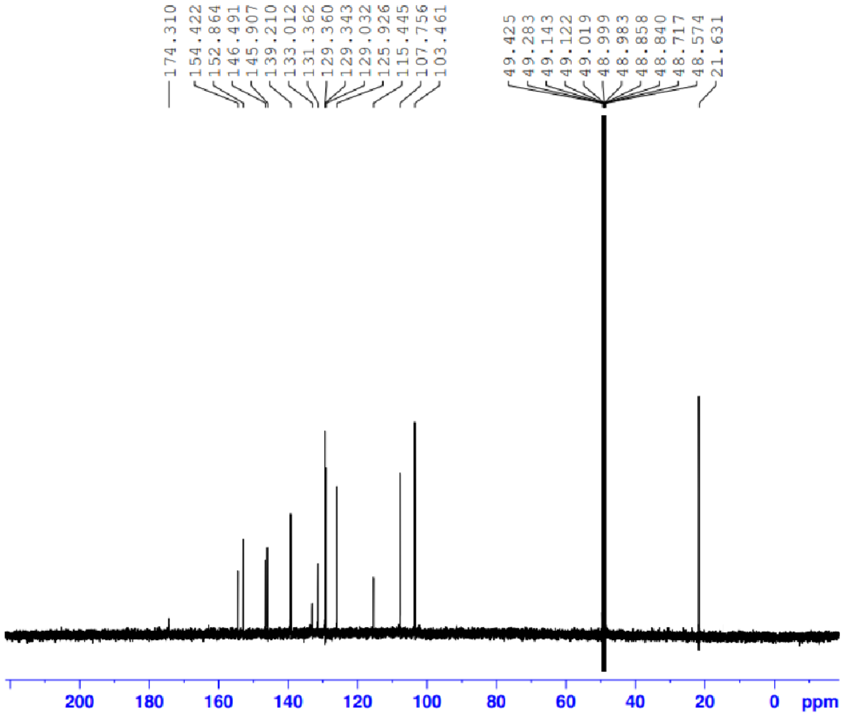

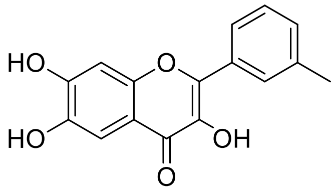

20d

HRMS chromatogram of compound **20d**

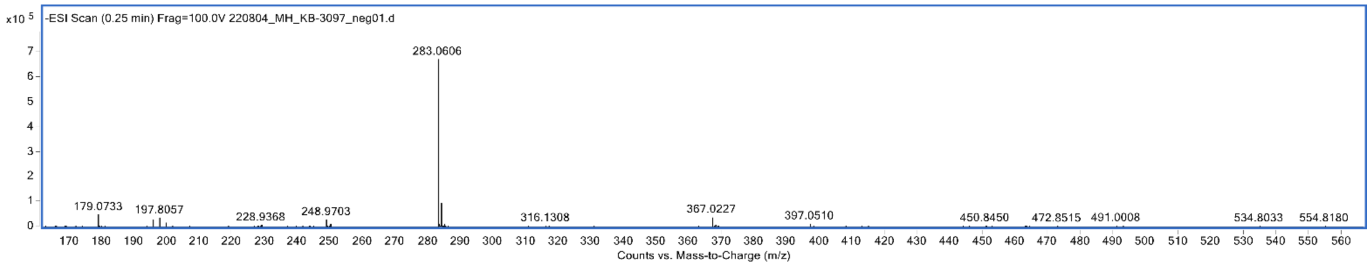

HPLC chromatogram of compound **20d** eluted using ACN/Water

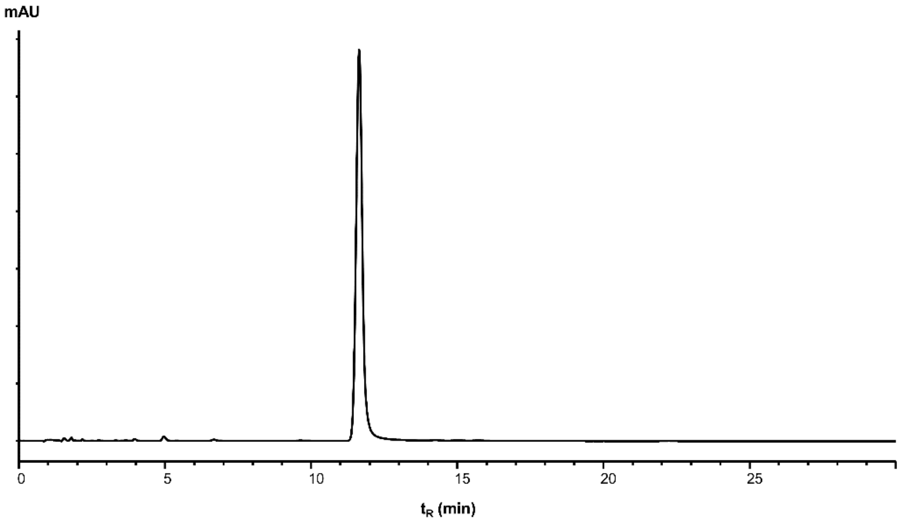

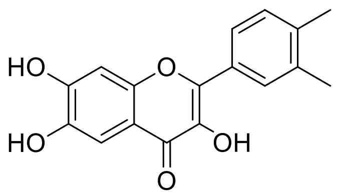

20e

^{1}H NMR spectra of compound **20e** measured in DMSO at 600 MHz

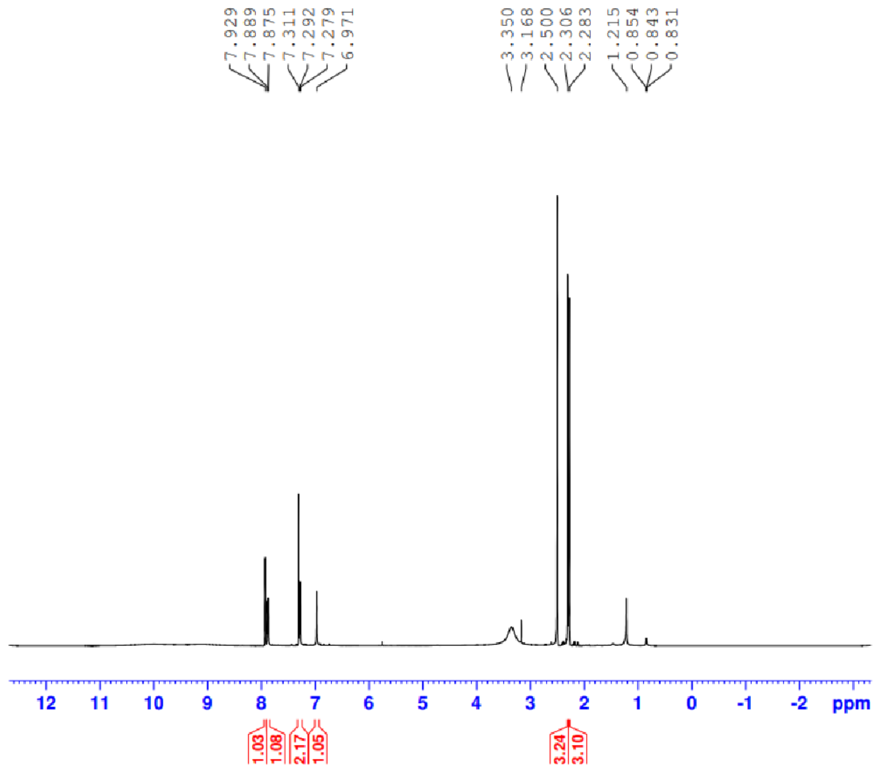

^{13}C NMR spectra of compound **20e** measured in DMSO at 600 MHz

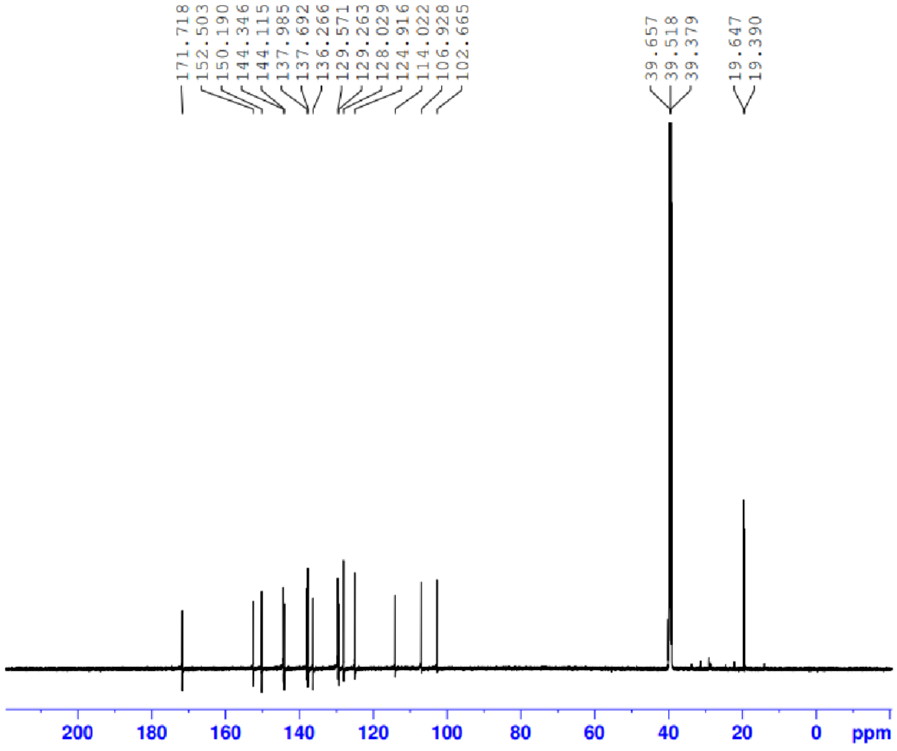

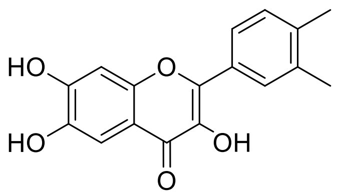

20e

HRMS chromatogram of compound **20e**

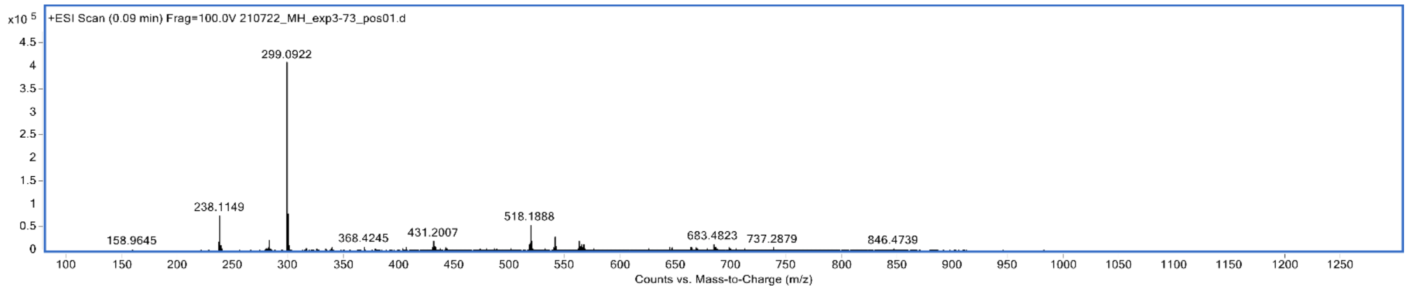

HPLC chromatogram of compound **20e** eluted using ACN/Water

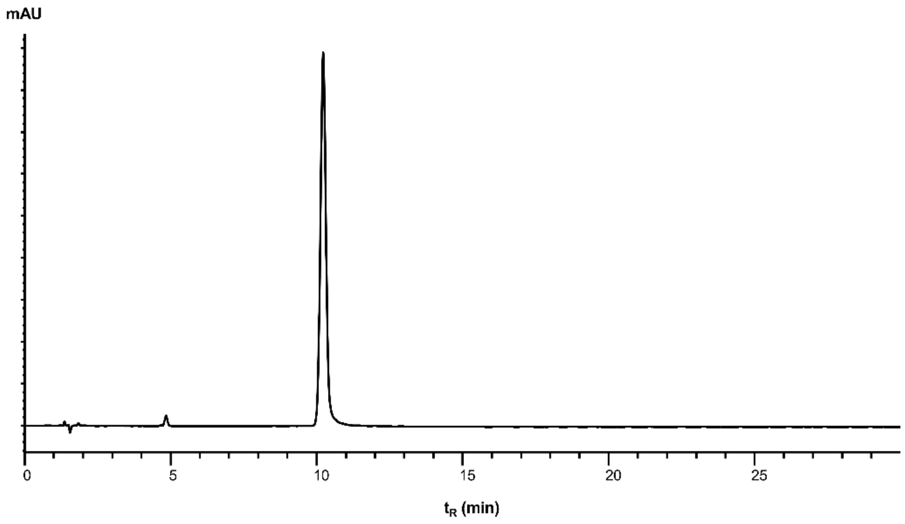

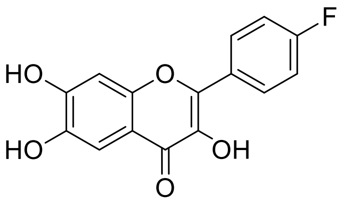

20f

^{1}H NMR spectra of compound **20f** measured in DMSO at 600 MHz

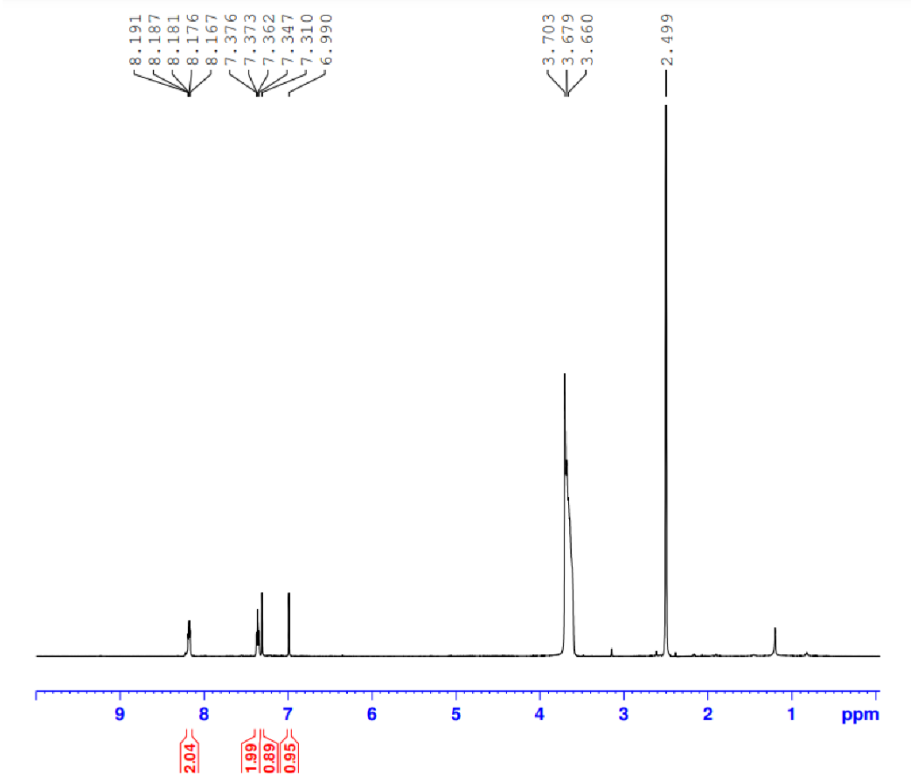

^{13}C NMR spectra of compound **20f** measured in DMSO at 600 MHz

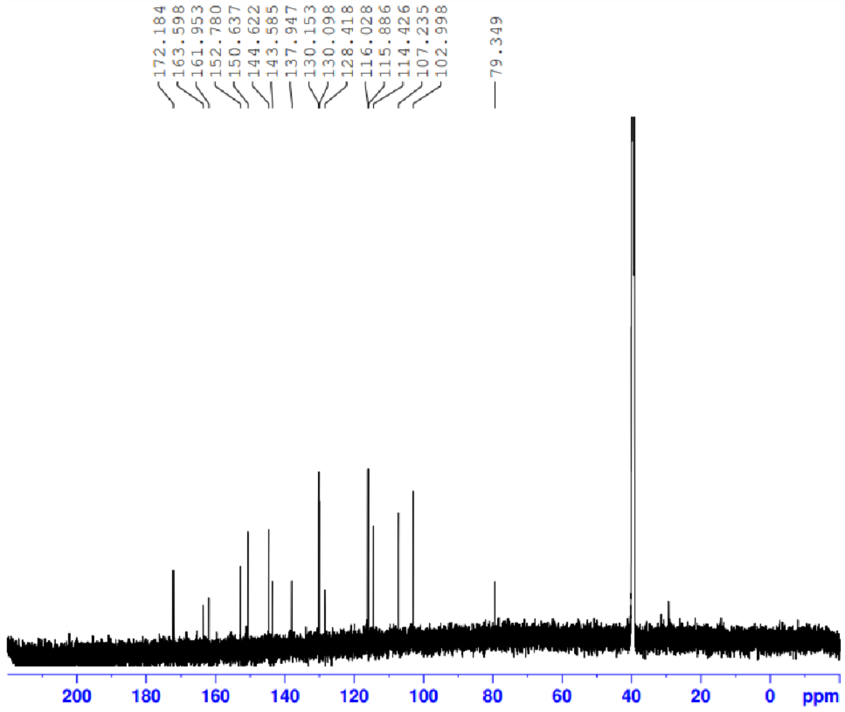

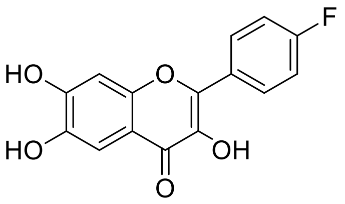

20f

HRMS chromatogram of compound **20f**

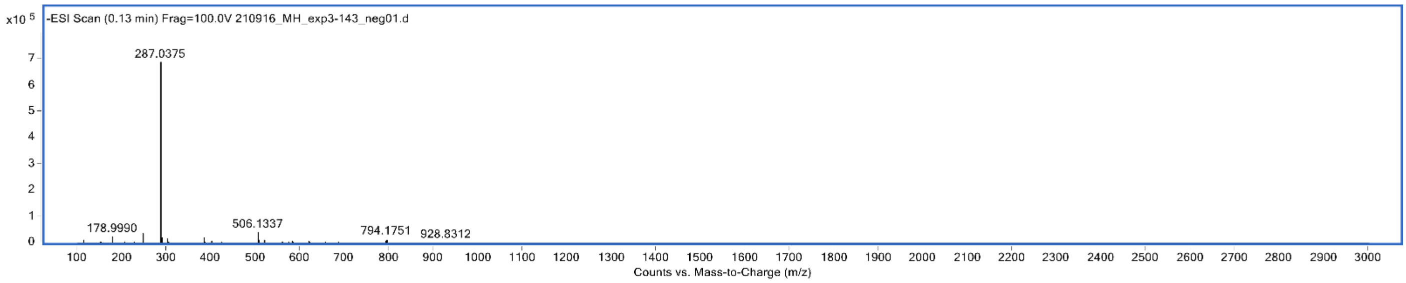

HPLC chromatogram of compound **20f** eluted using ACN/Water

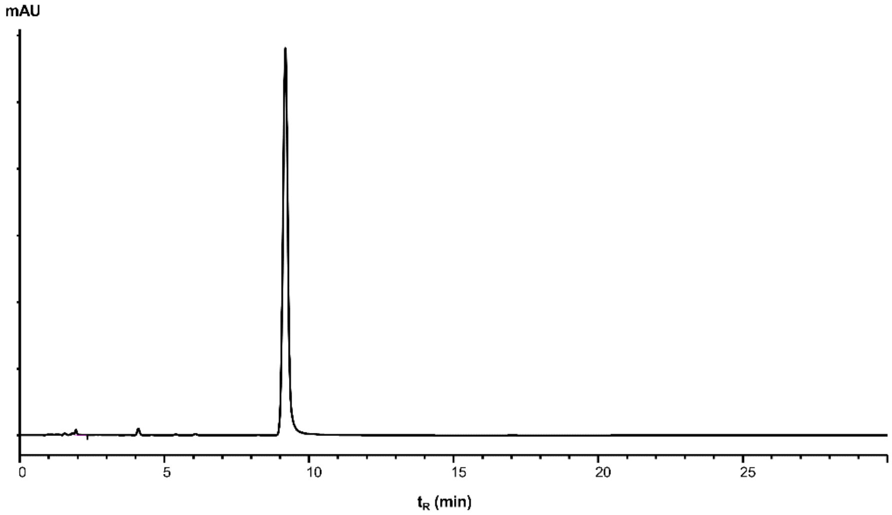

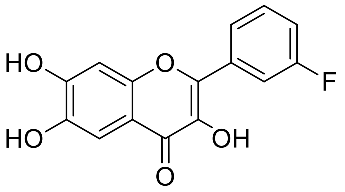

20g

^{1}H NMR spectra of compound **20g** measured in DMSO at 600 MHz

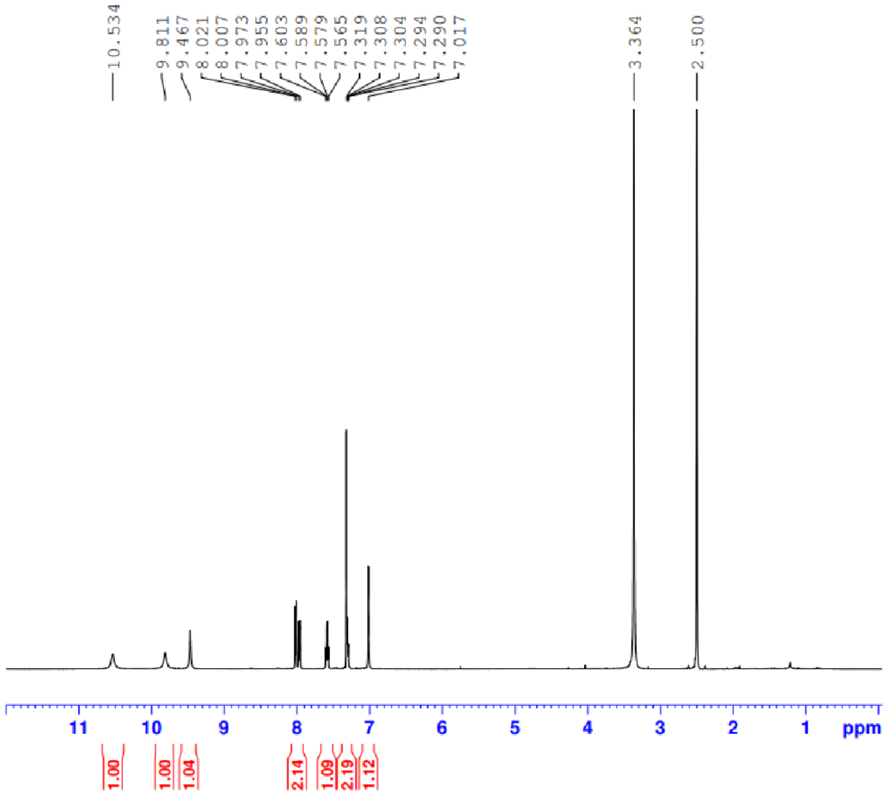

^{13}C NMR spectra of compound **20g** measured in DMSO at 600 MHz

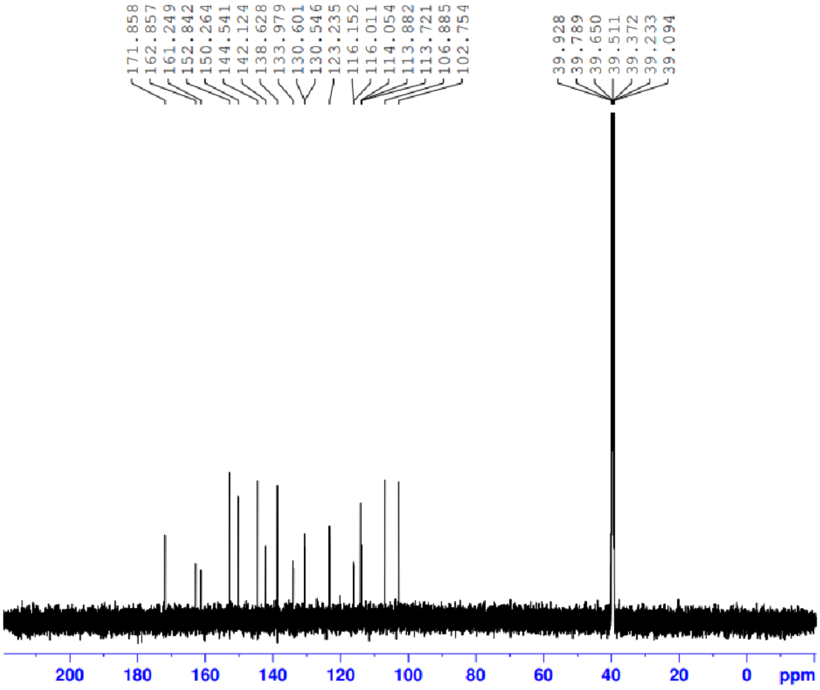

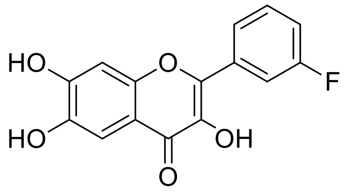

20g

HRMS chromatogram of compound **20g**

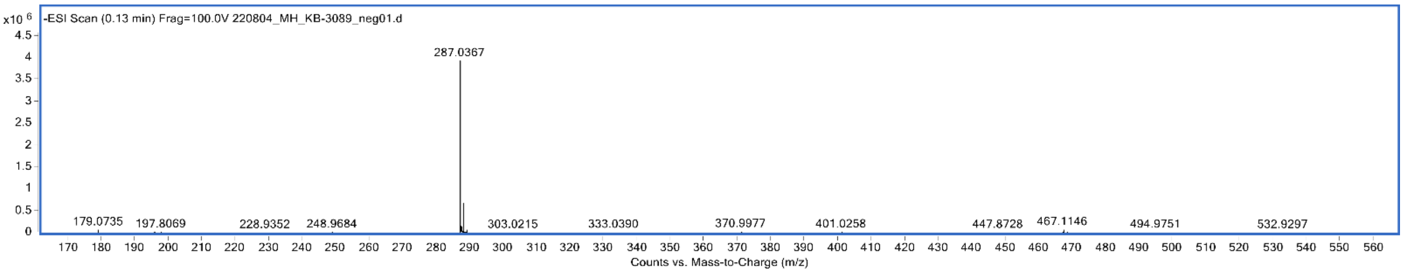

HPLC chromatogram of compound **20g** eluted using ACN/Water

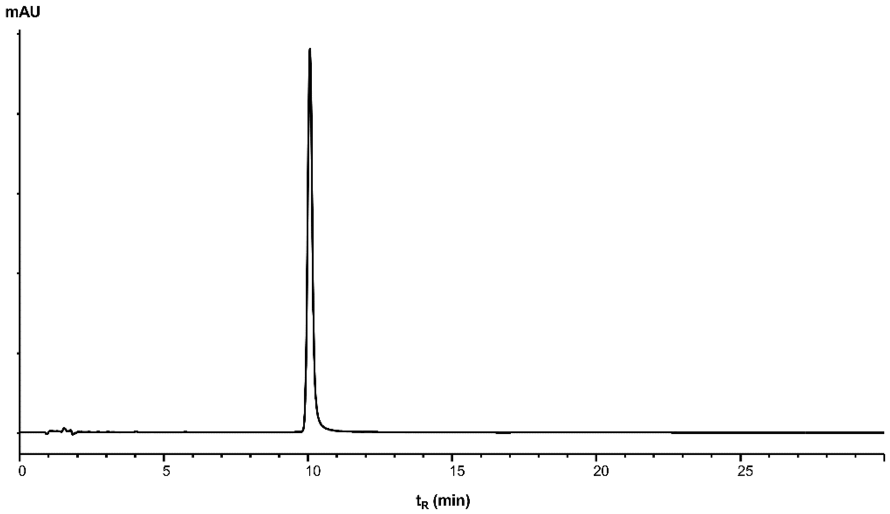

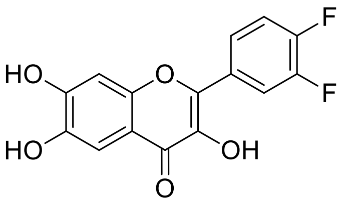

20h

^{1}H NMR spectra of compound **20h** measured in DMSO at 600 MHz

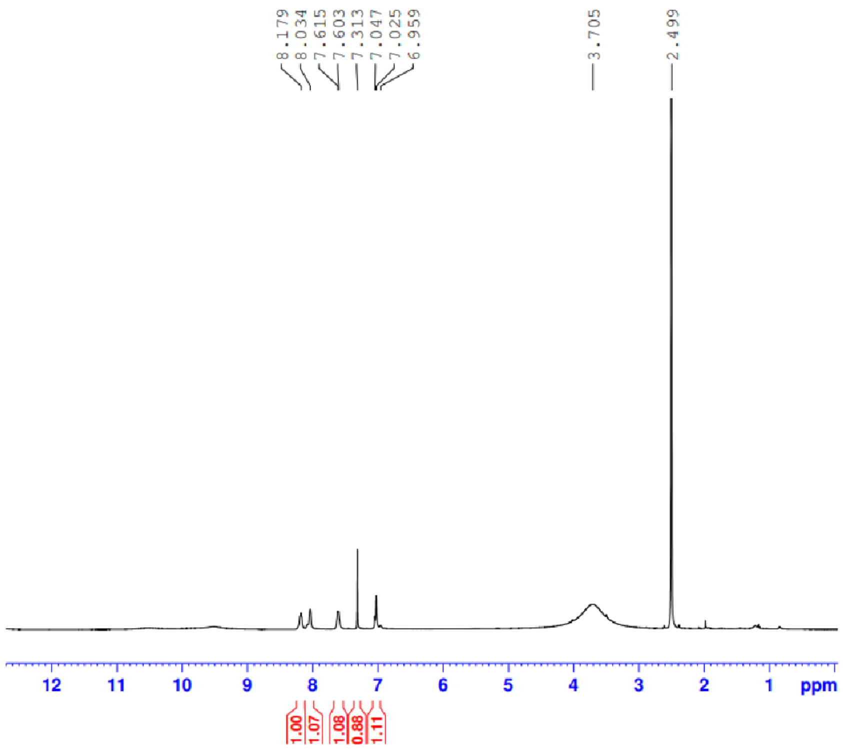

^{13}C NMR spectra of compound **20h** measured in DMSO at 600 MHz

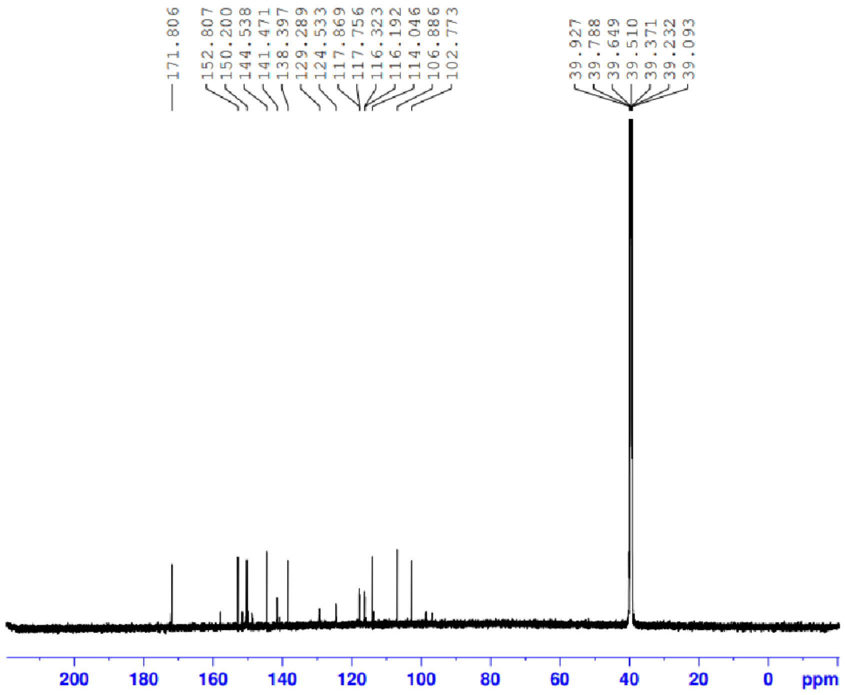

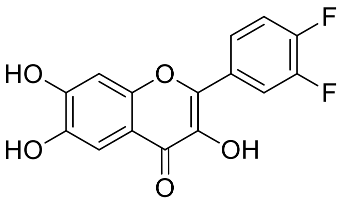

20h

HRMS chromatogram of compound **20h**

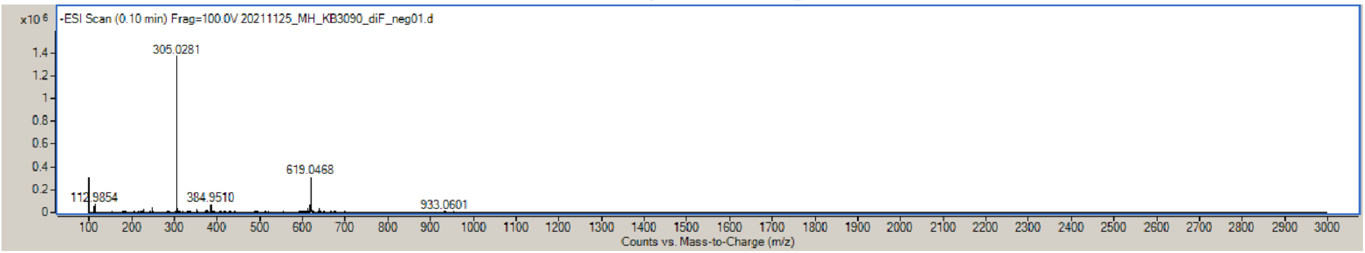

HPLC chromatogram of compound **20h** eluted using ACN/Water

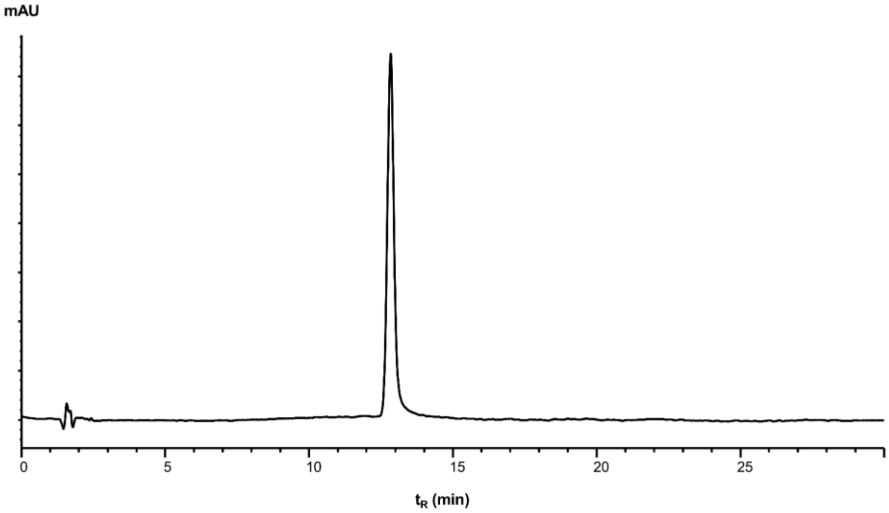

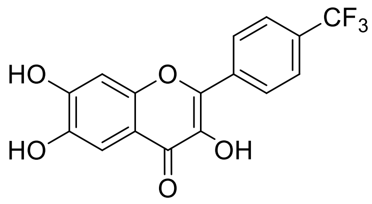

20i

^{1}H NMR spectra of compound **20i** measured in DMSO at 600 MHz

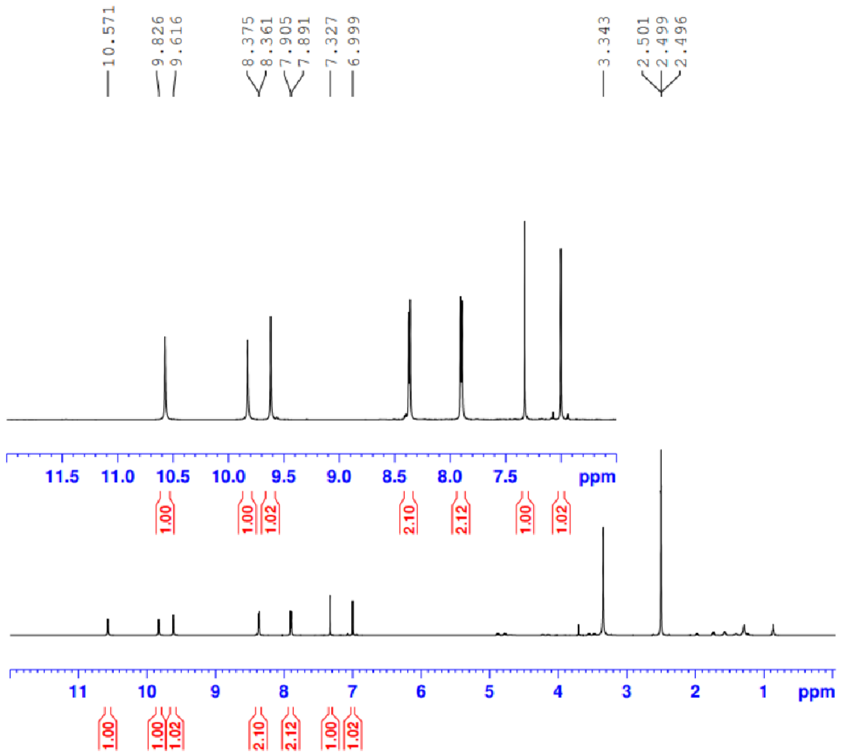

^{13}C NMR spectra of compound **20i** measured in DMSO at 600 MHz

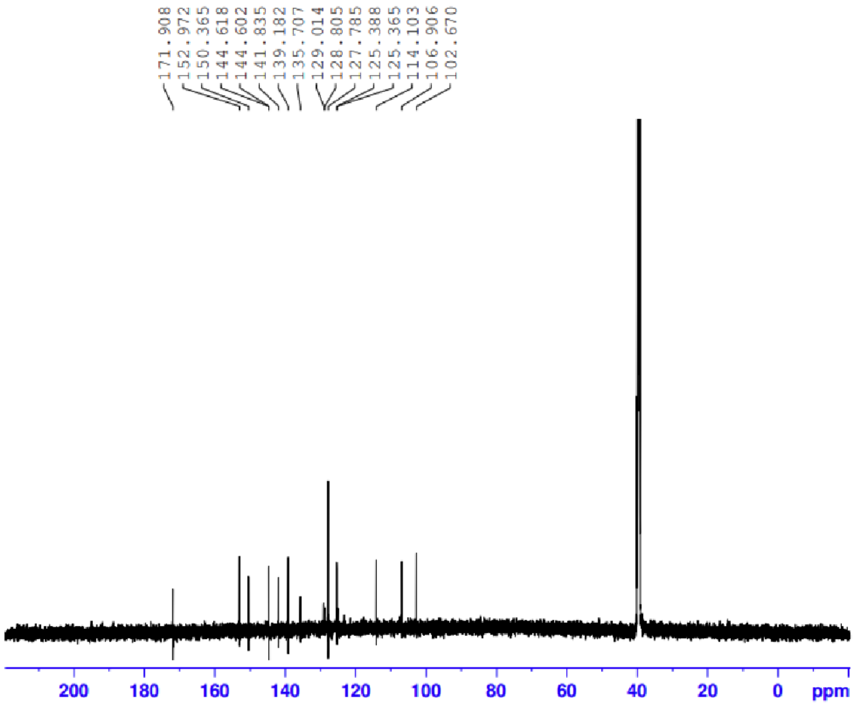

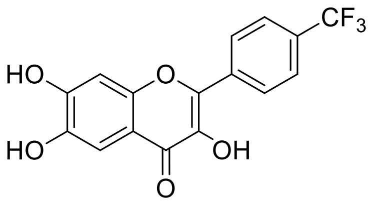

20i

HRMS chromatogram of compound **20i**

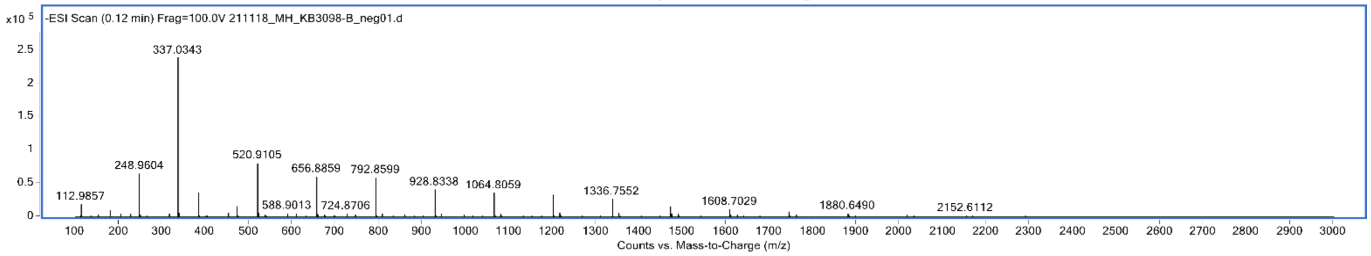

HPLC chromatogram of compound **20i** eluted using ACN/Water

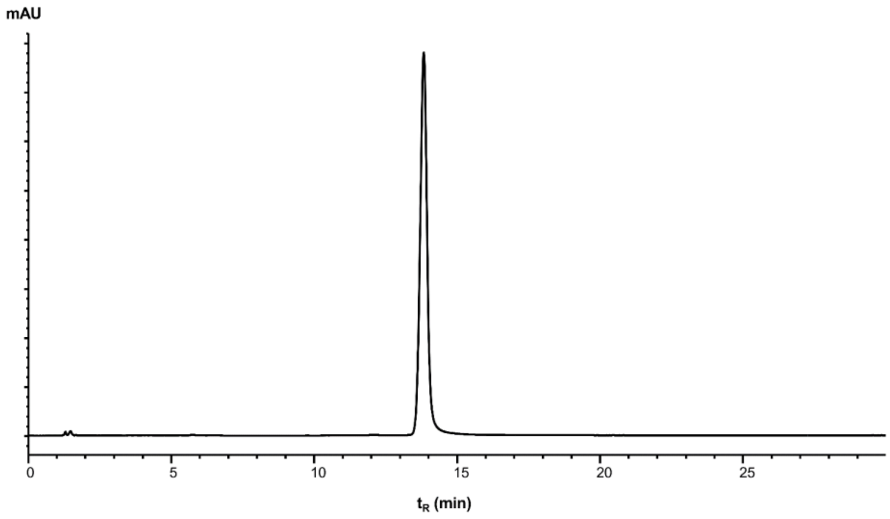

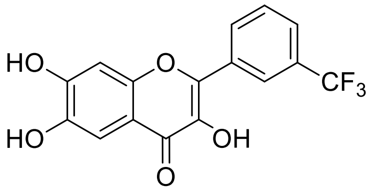

20j

^{1}H NMR spectra of compound **20j** measured in MeOD at 600 MHz

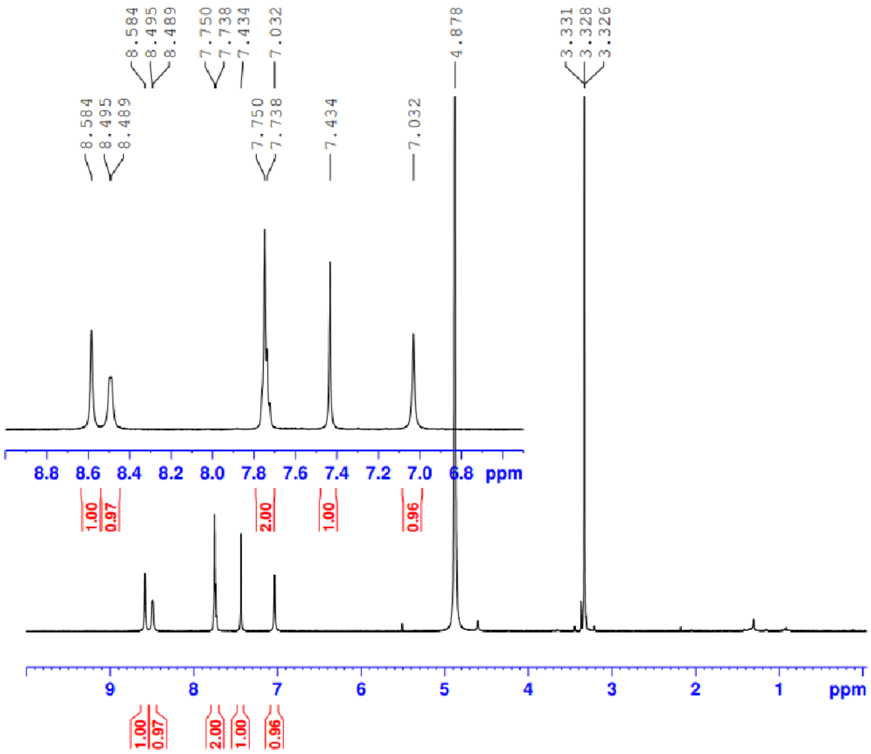

^{13}C NMR spectra of compound **20j** measured in DMSO at 600 MHz

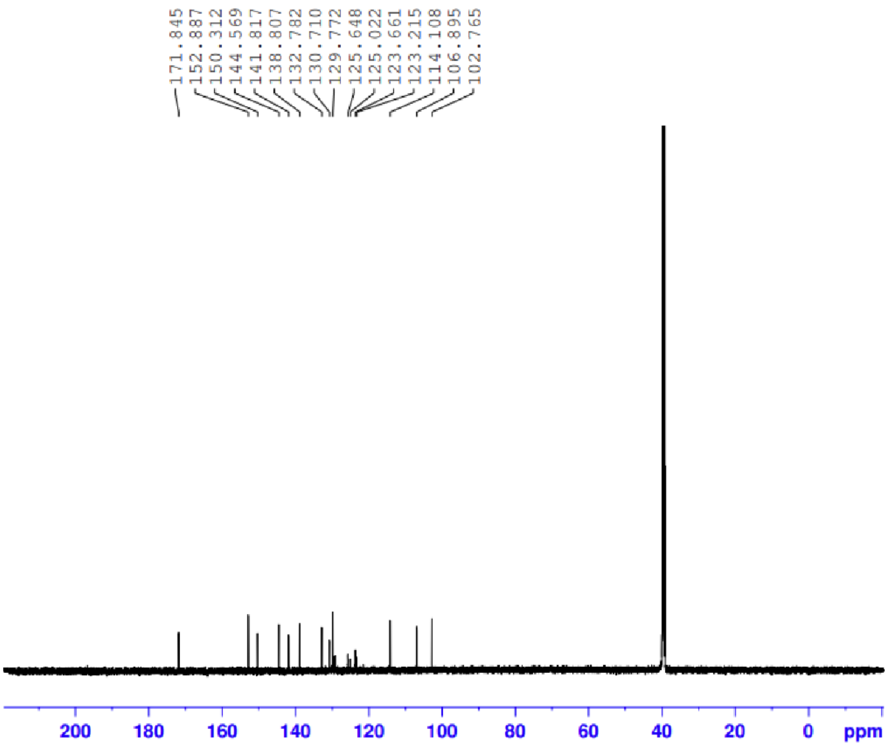

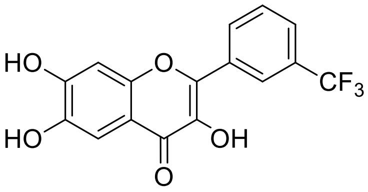

20j

HRMS chromatogram of compound **20j**

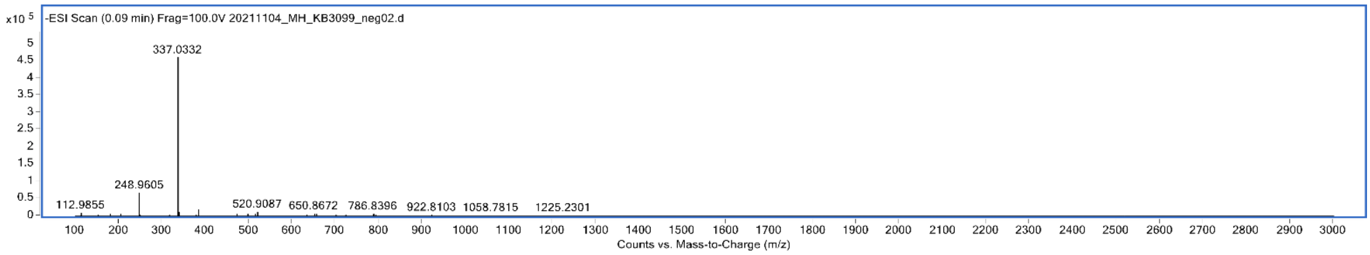

HPLC chromatogram of compound **20j** eluted using ACN/Water

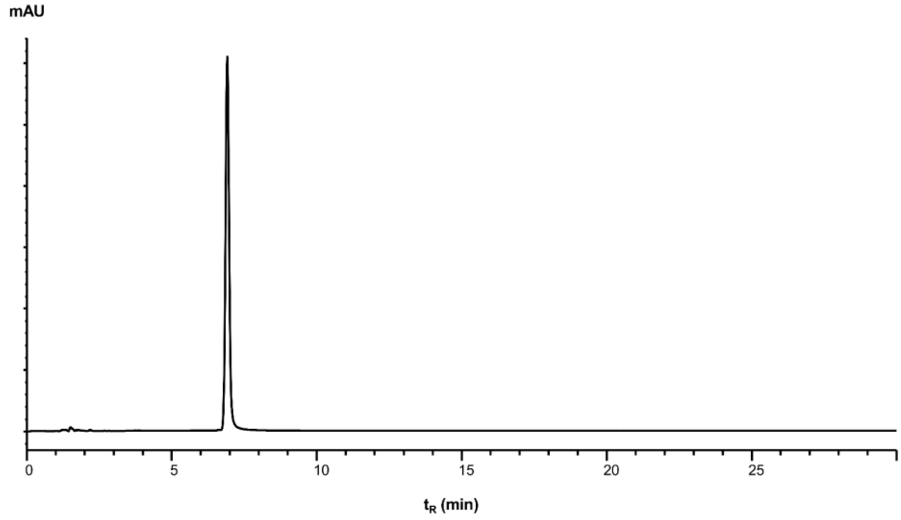

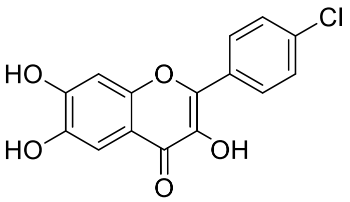

20k

^{1}H NMR spectra of compound **20k** measured in DMSO at 600 MHz

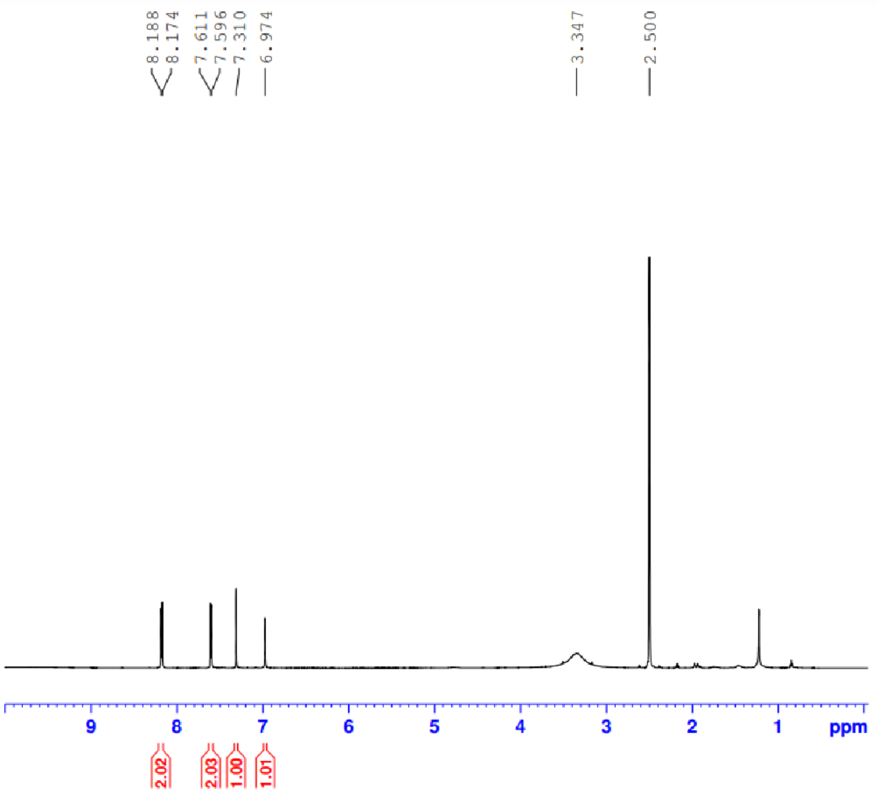

^{13}C NMR spectra of compound **20k** measured in DMSO at 600 MHz

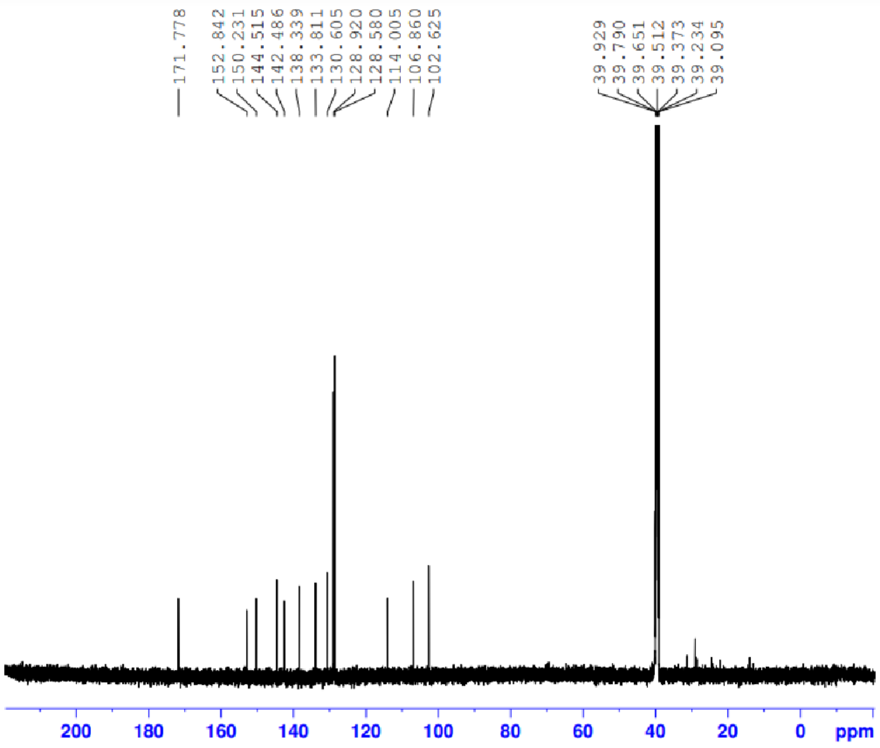

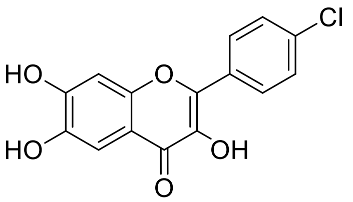

20k

HRMS chromatogram of compound **20k**

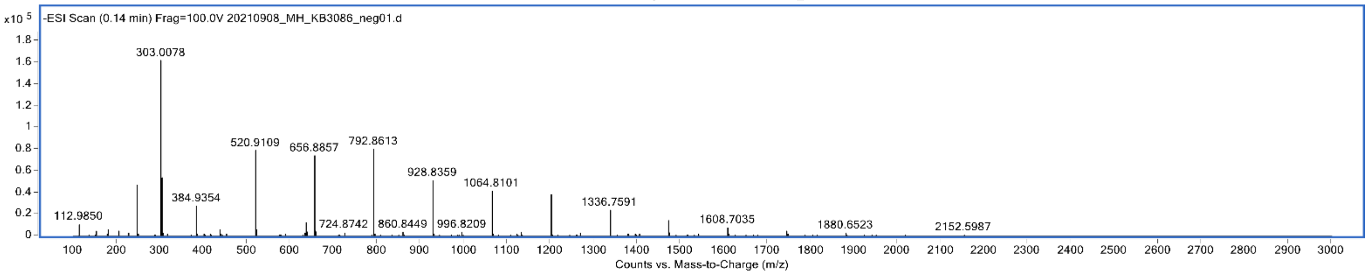

HPLC chromatogram of compound **20k** eluted using ACN/Water

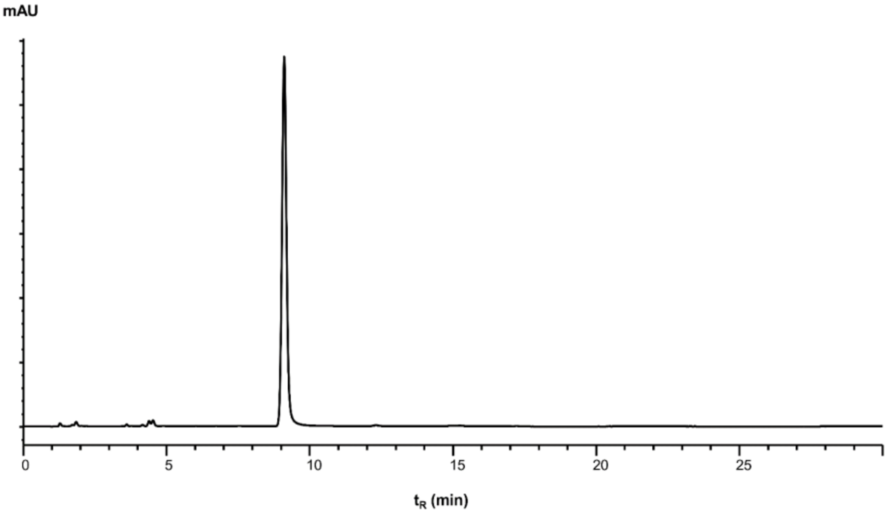

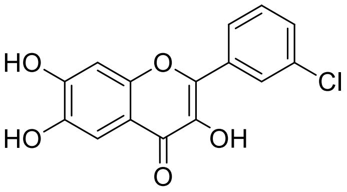

20l

^{1}H NMR spectra of compound **20l** measured in DMSO at 600 MHz

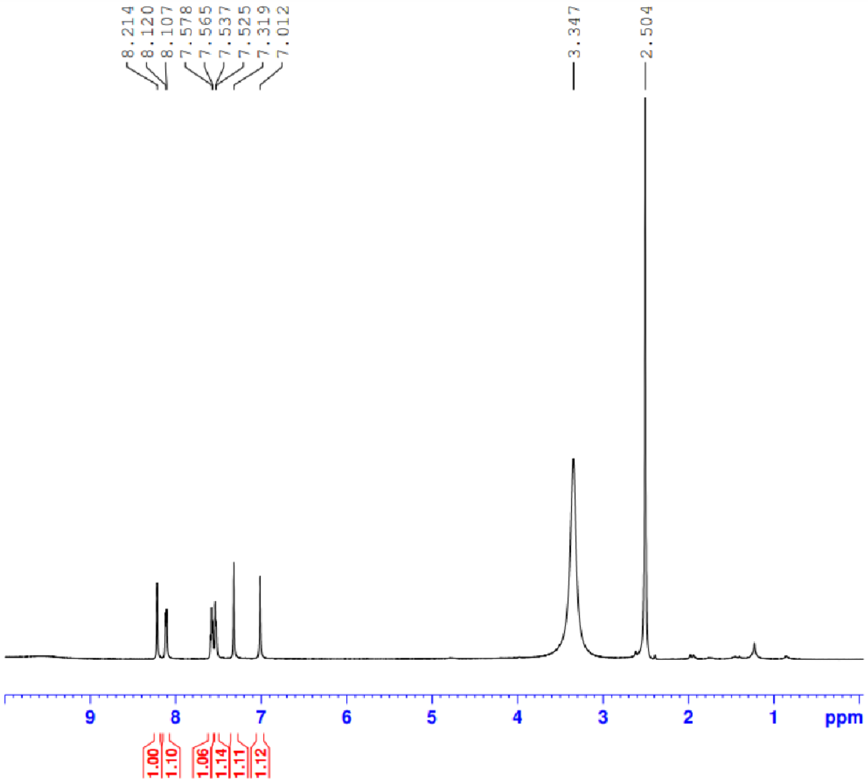

^{13}C NMR spectra of compound **20l** measured in DMSO at 600 MHz

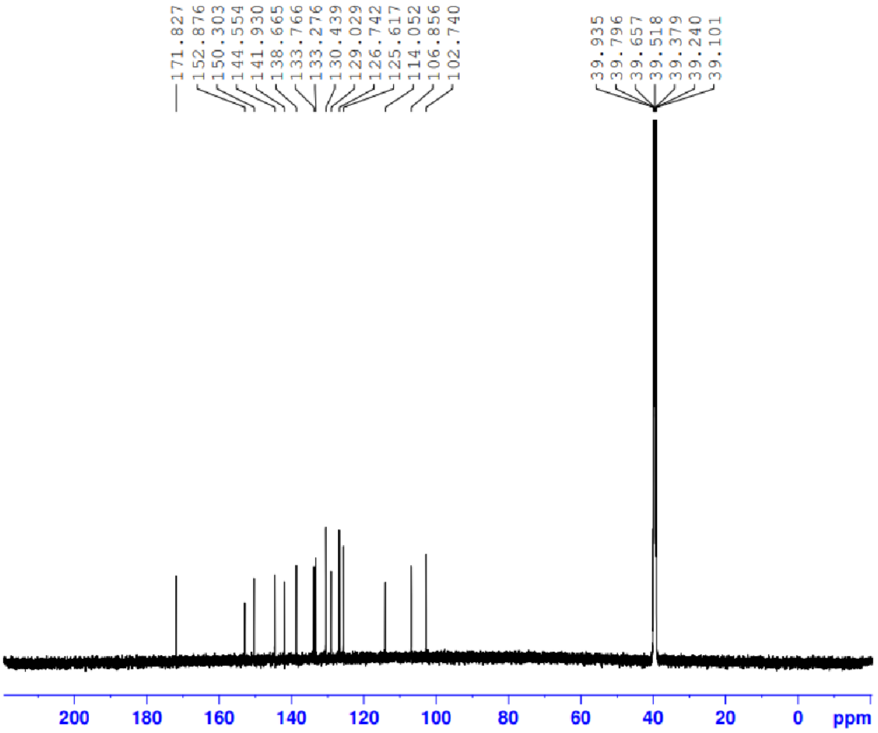

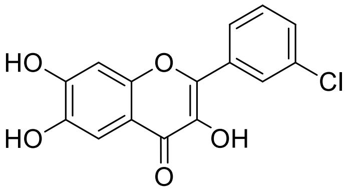

20l

HRMS chromatogram of compound **20l**

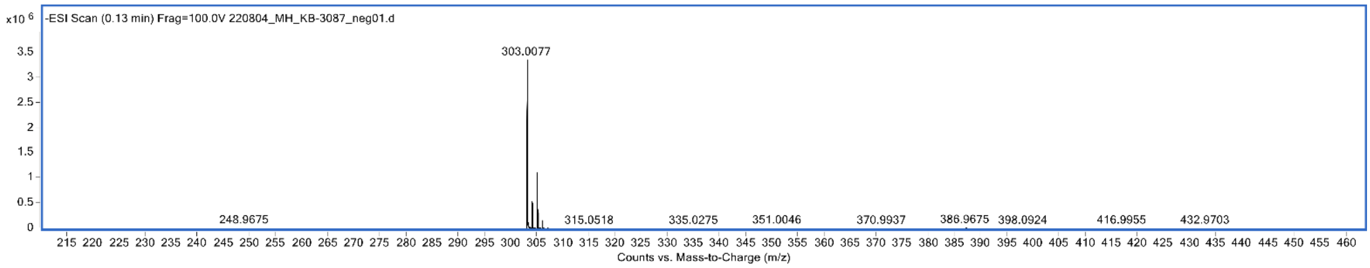

HPLC chromatogram of compound **20l** eluted using ACN/Water

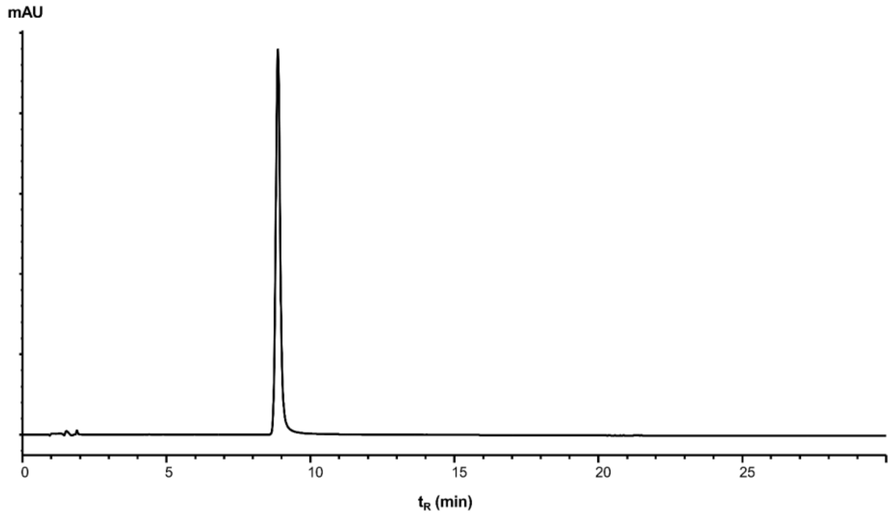

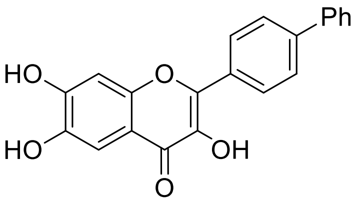

20m

^{1}H NMR spectra of compound **20m** measured in DMSO at 600 MHz

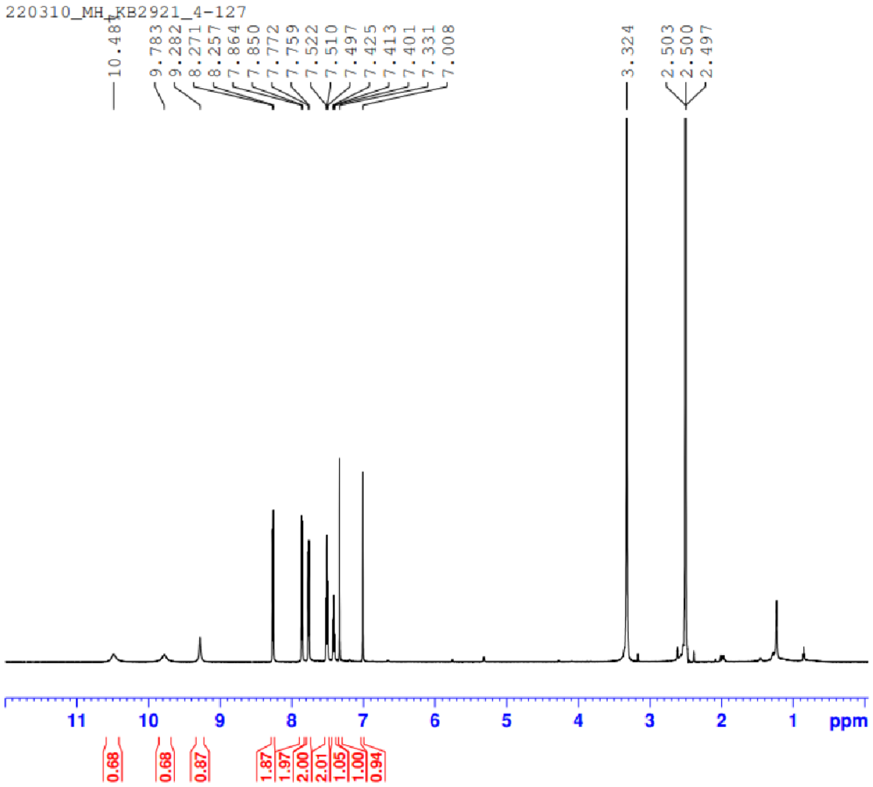

^{13}C NMR spectra of compound **20m** measured in DMSO at 600 MHz

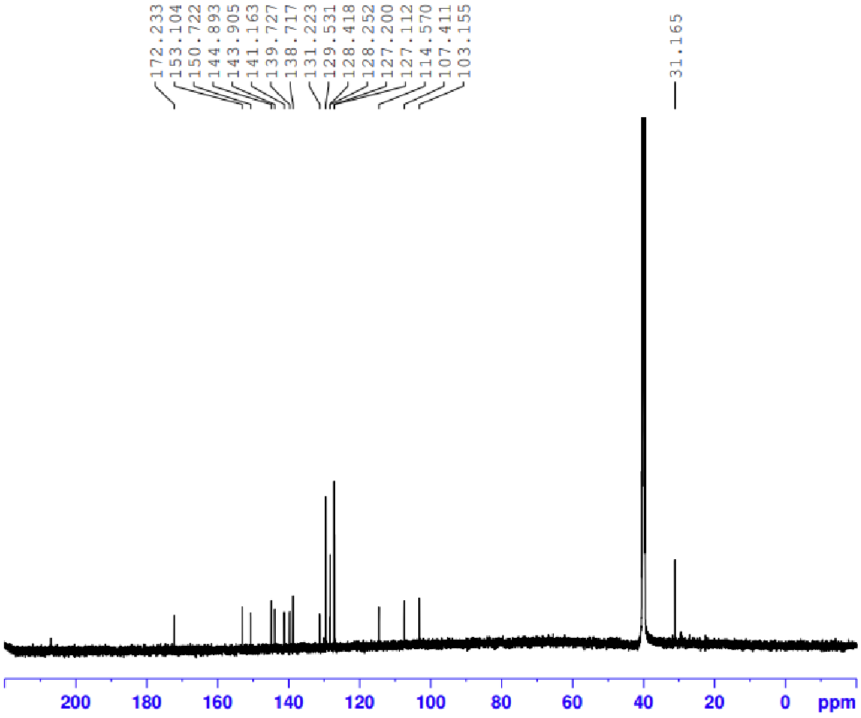

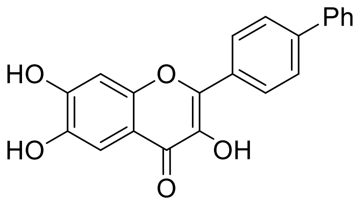

20m

HRMS chromatogram of compound **20m**

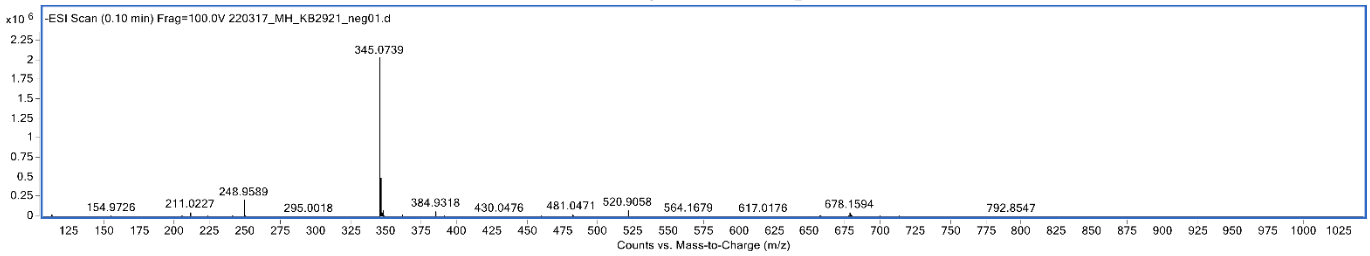

HPLC chromatogram of compound **20m** eluted using ACN/Water

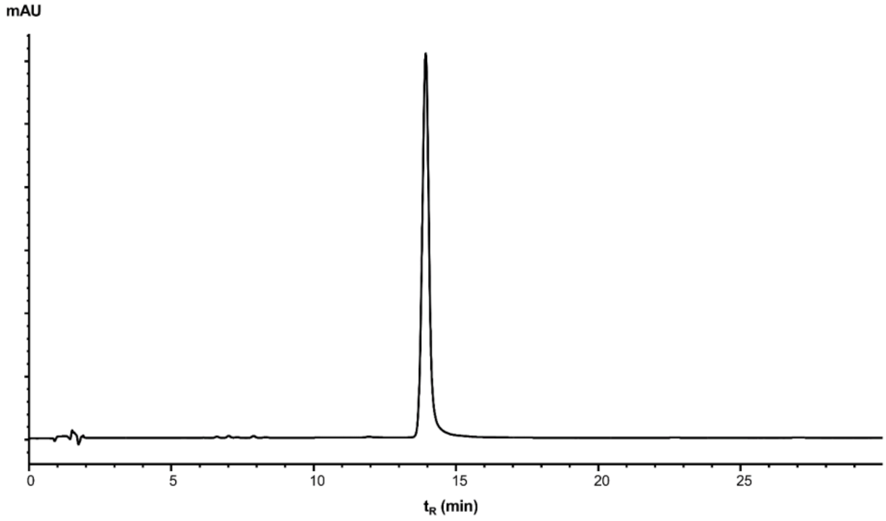

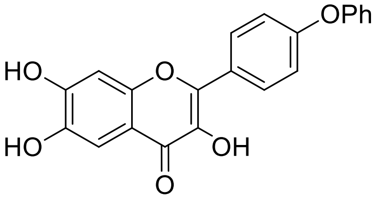

20n

^{1}H NMR spectra of compound **20n** measured in DMSO at 600 MHz

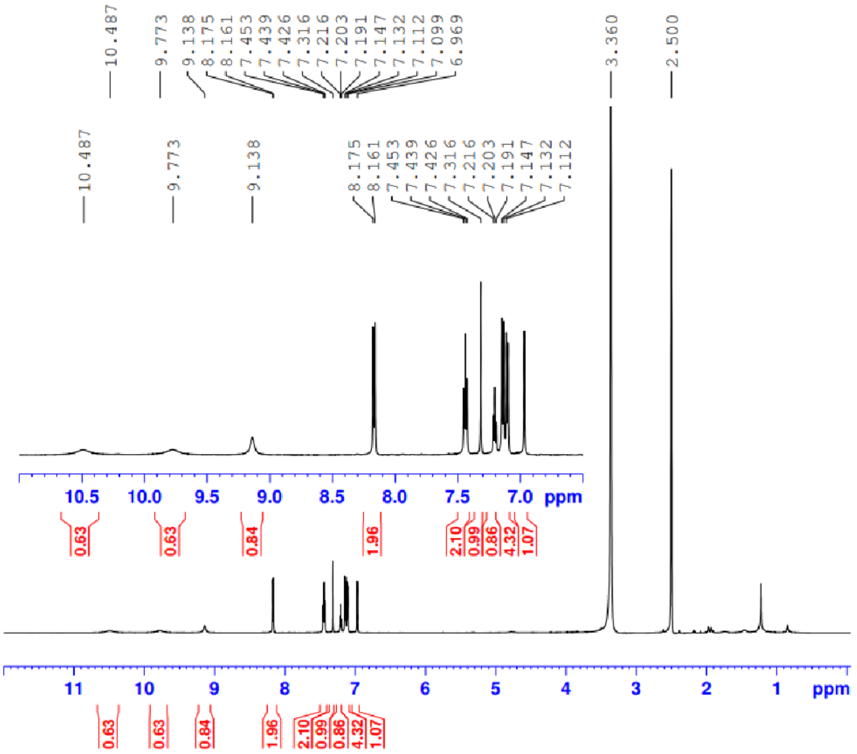

^{13}C NMR spectra of compound **20n** measured in DMSO at 600 MHz

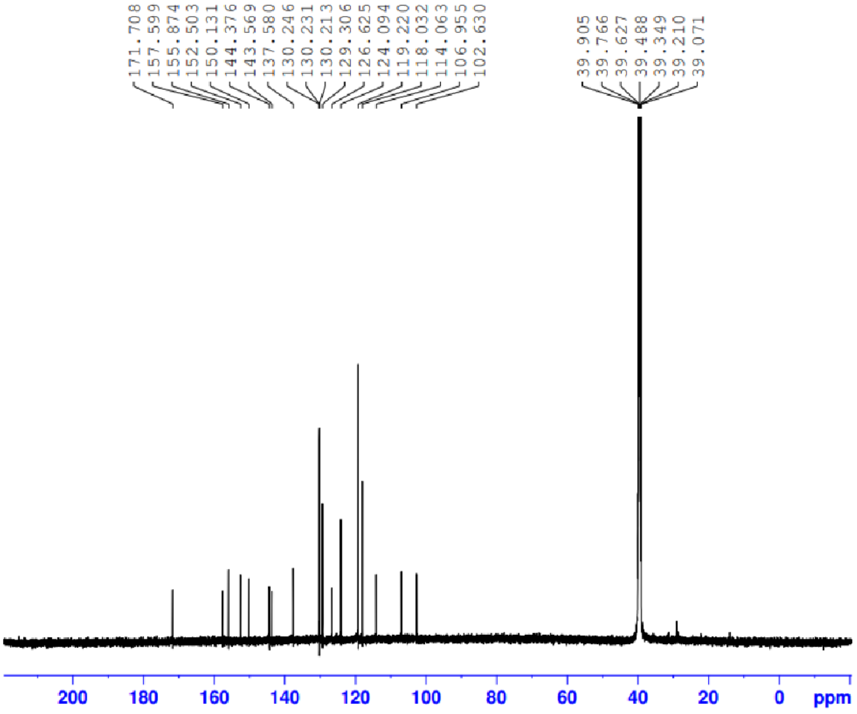

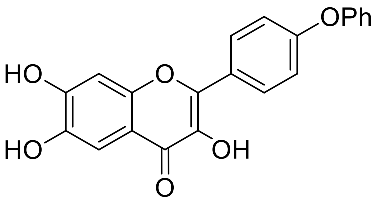

20n

HRMS chromatogram of compound **20n**

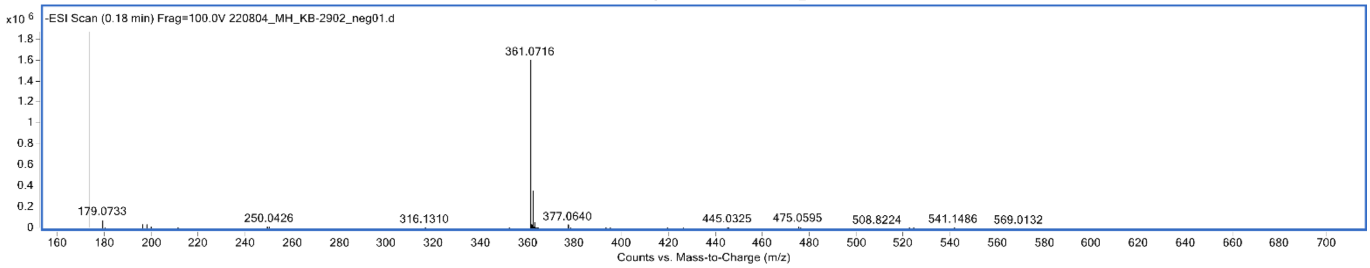

HPLC chromatogram of compound **20n** eluted using ACN/Water

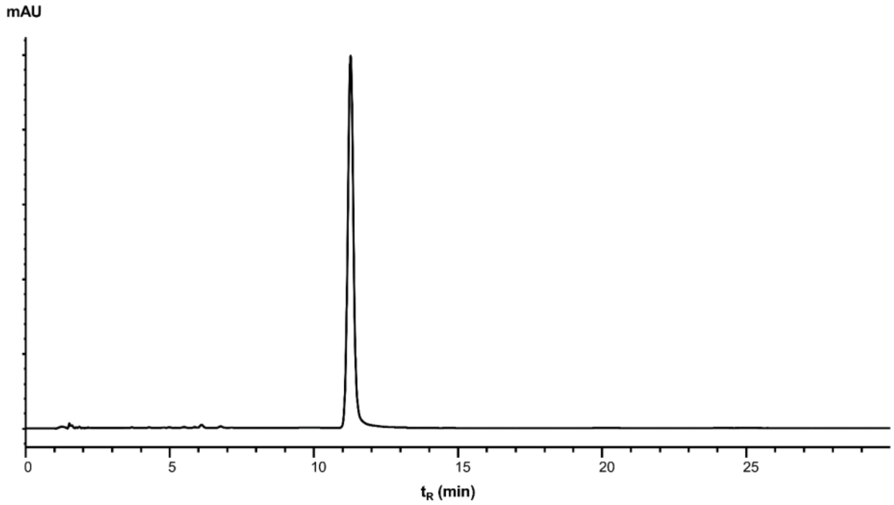

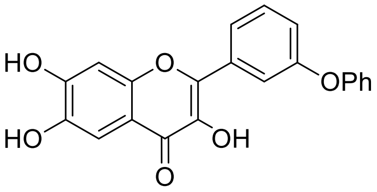

20o

^{1}H NMR spectra of compound **20o** measured in DMSO at 600 MHz

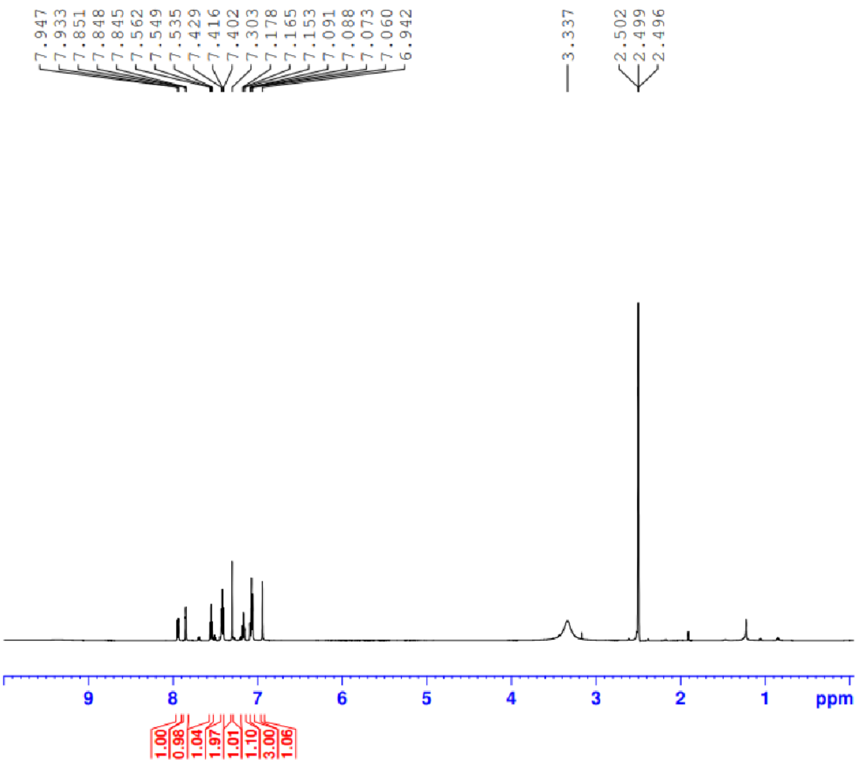

^{13}C NMR spectra of compound **20o** measured in DMSO at 600 MHz

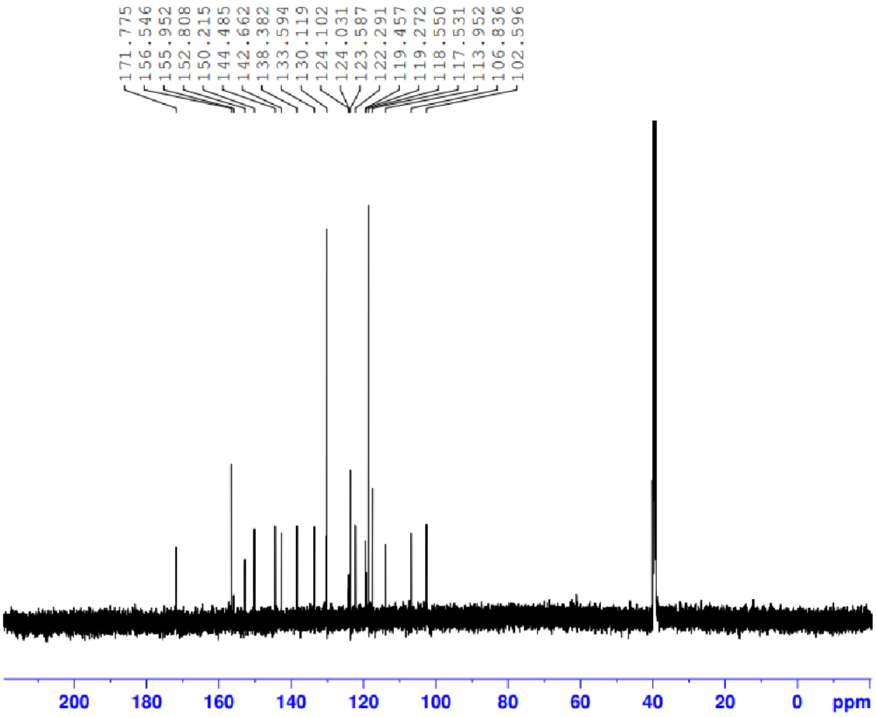

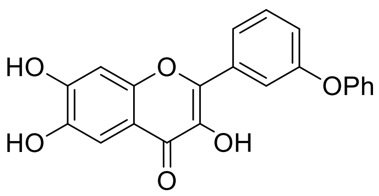

20o

HRMS chromatogram of compound **20o**

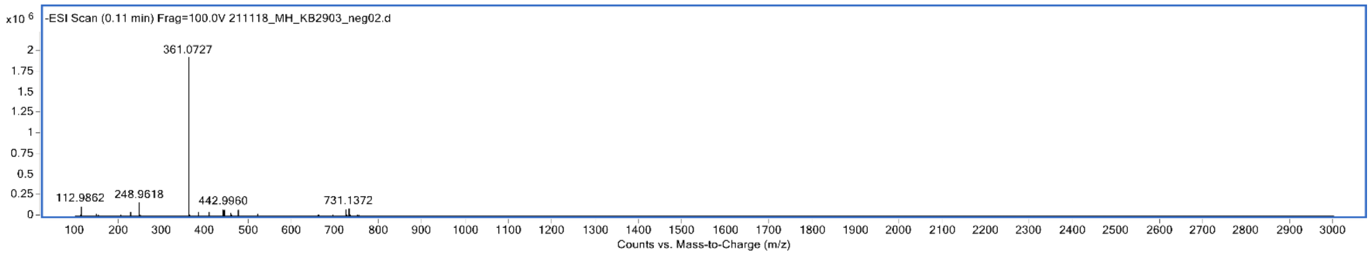

HPLC chromatogram of compound **20o** eluted using ACN/Water

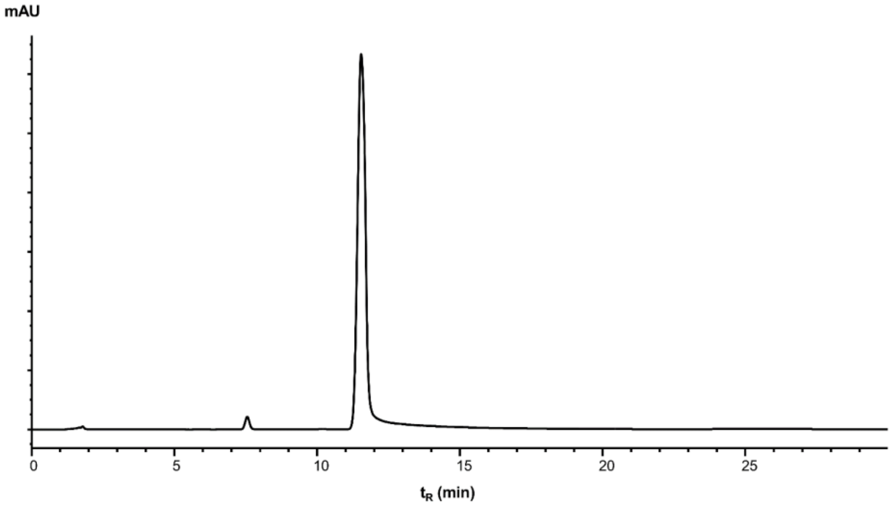

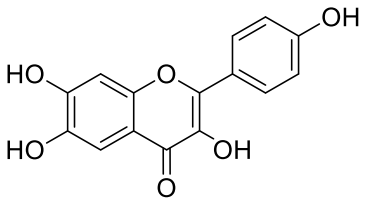

20p

^{1}H NMR spectra of compound **20p** measured in DMSO at 600 MHz

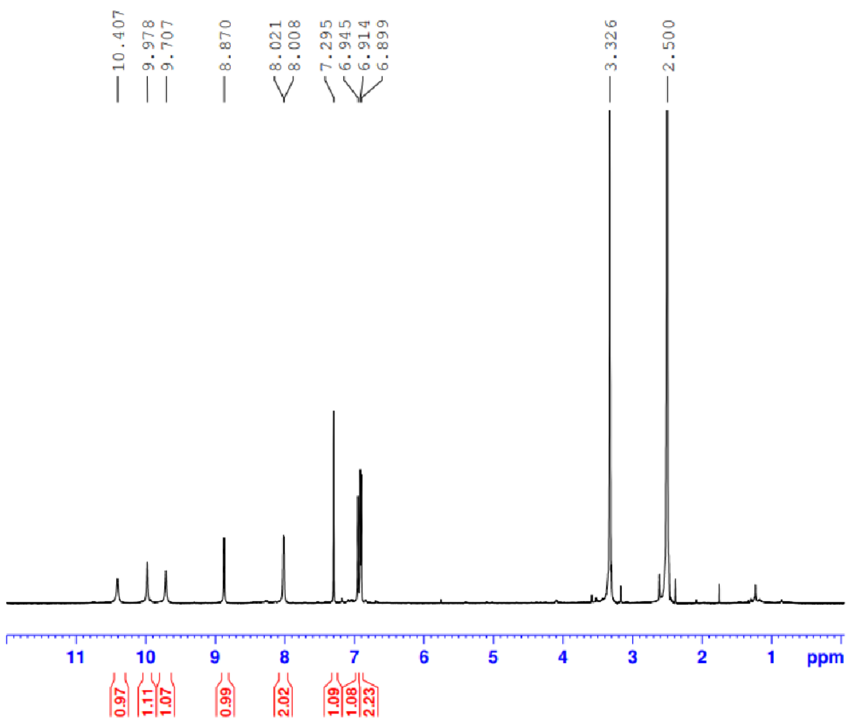

HRMS chromatogram of compound **20p**

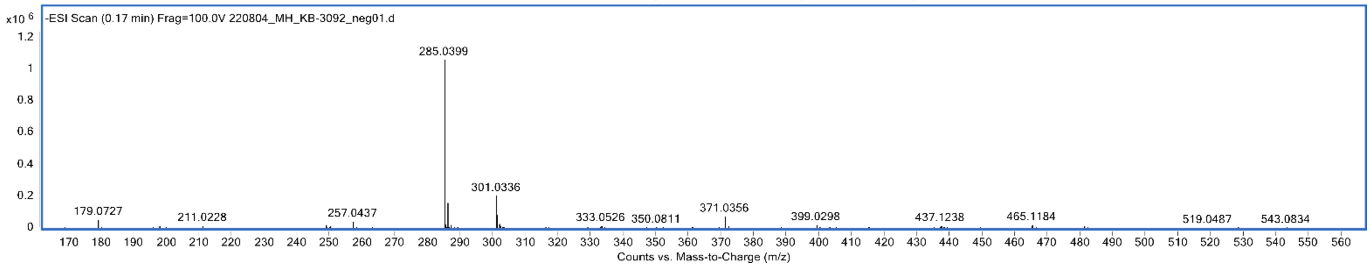

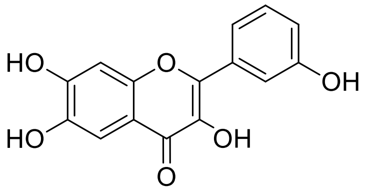

20q

^{1}H NMR spectra of compound **20q** measured in DMSO at 600 MHz

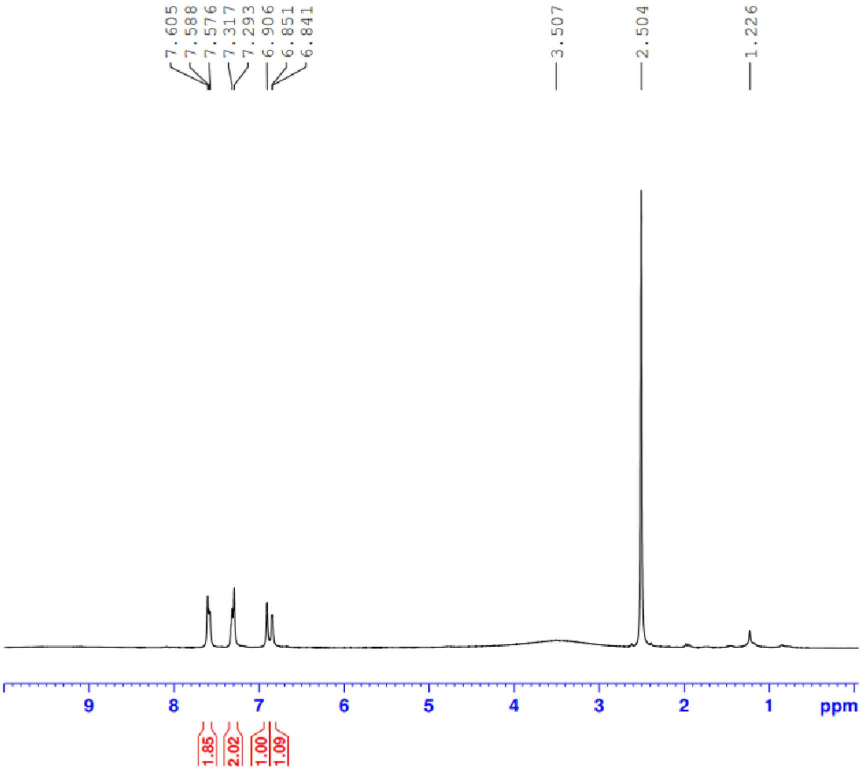

HRMS chromatogram of compound **20q**

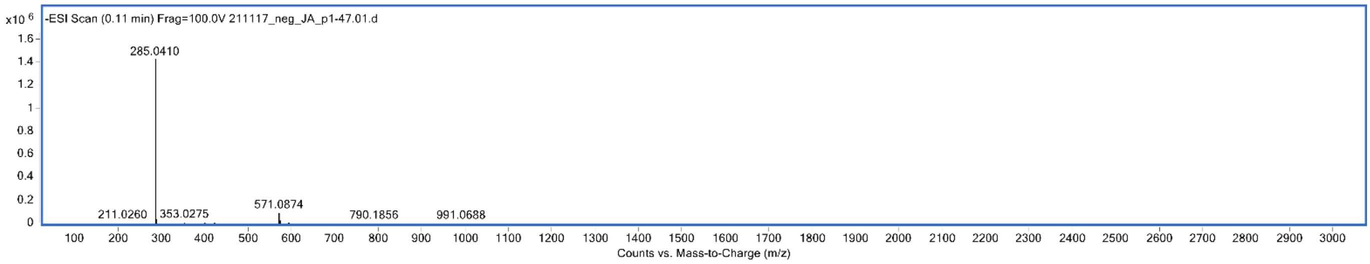

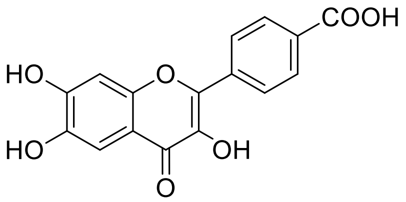

20r

^{1}H NMR spectra of compound **20r** measured in MeOD at 600 MHz

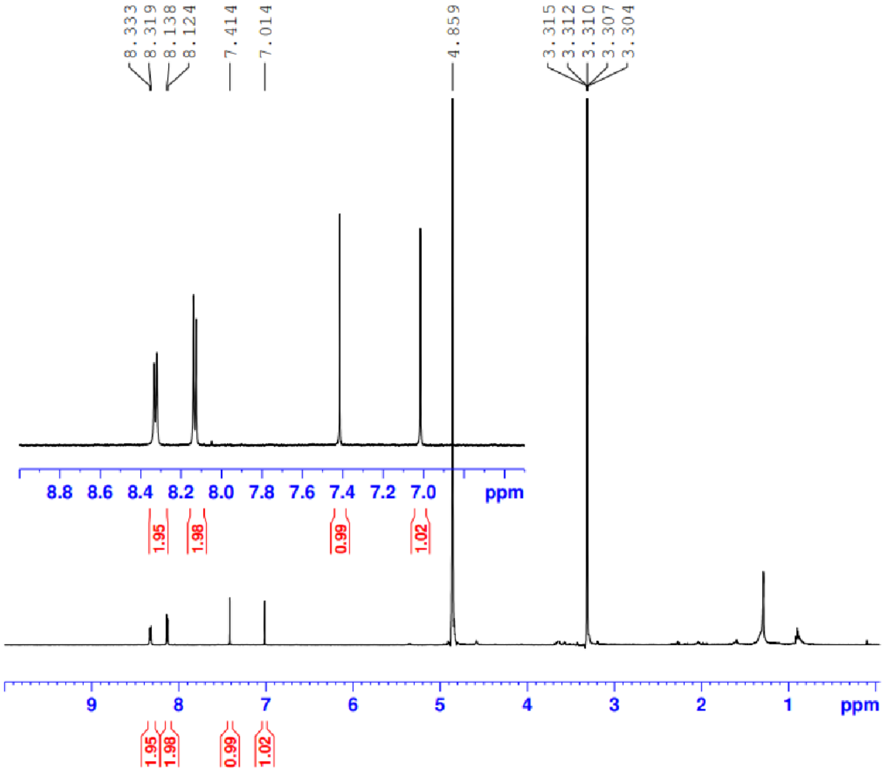

^{13}C NMR spectra of compound **20r** measured in DMSO at 600 MHz

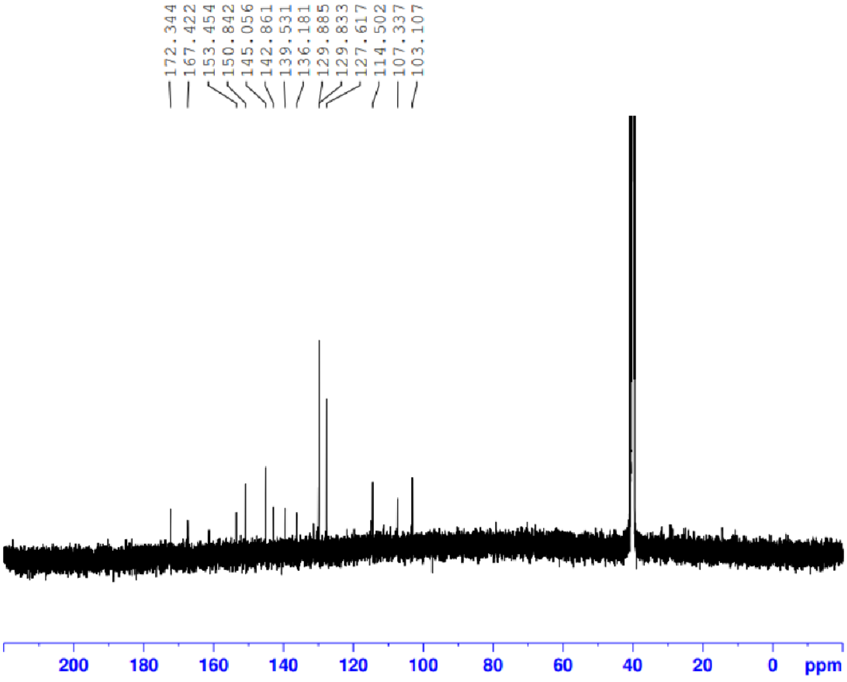

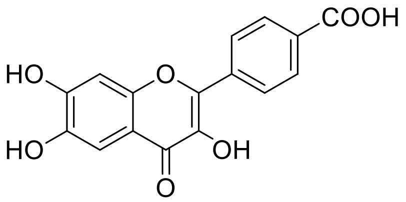

20r

HRMS chromatogram of compound **20r**

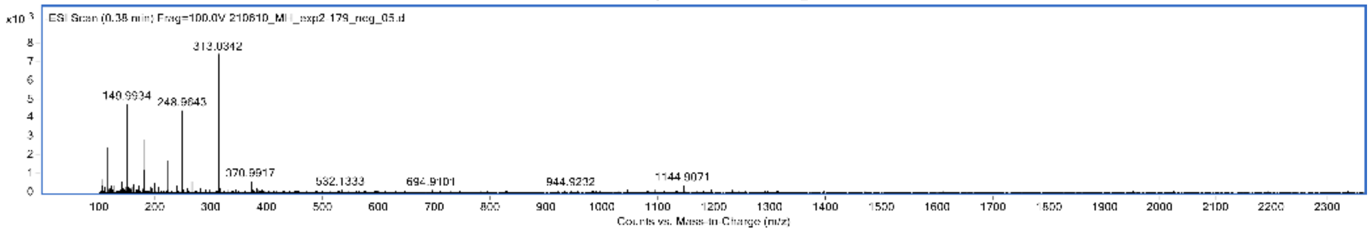

HPLC chromatogram of compound **20r** eluted using ACN/Water

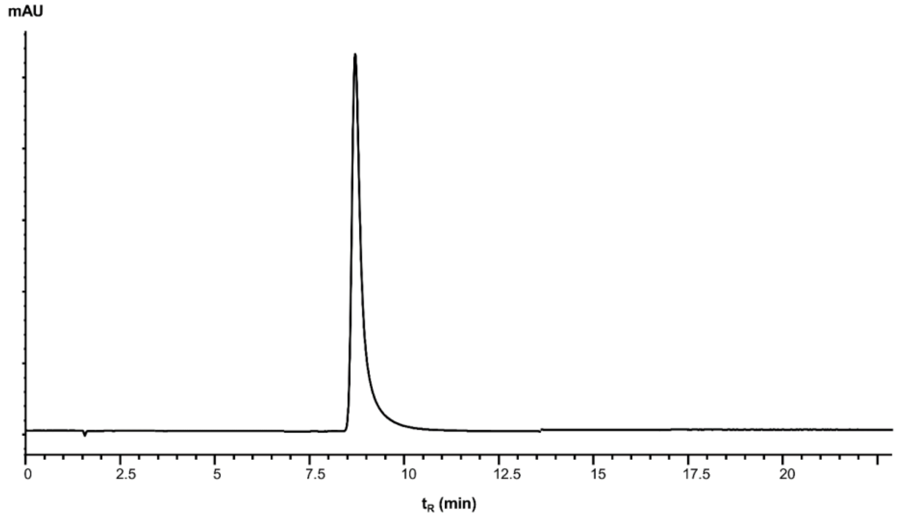

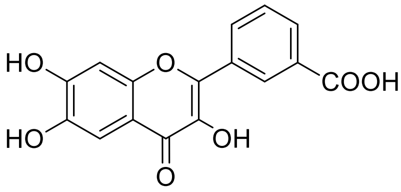

20s

^{1}H NMR spectra of compound **20s** measured in MeOD at 600 MHz

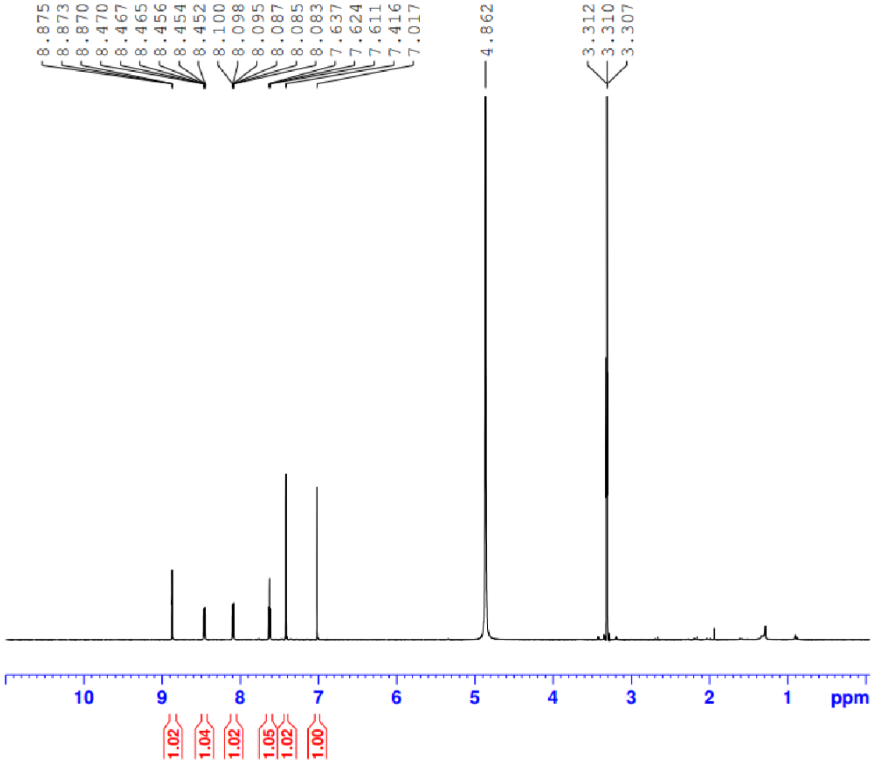

^{13}C NMR spectra of compound **20s** measured in DMSO at 600 MHz

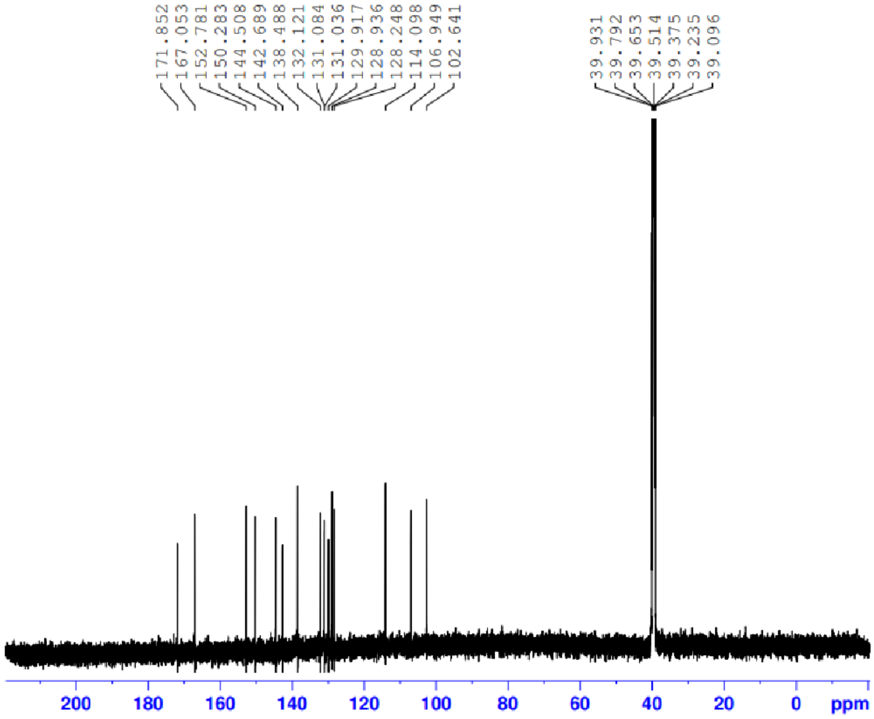

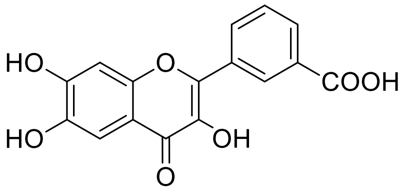

20s

HRMS chromatogram of compound **20s**

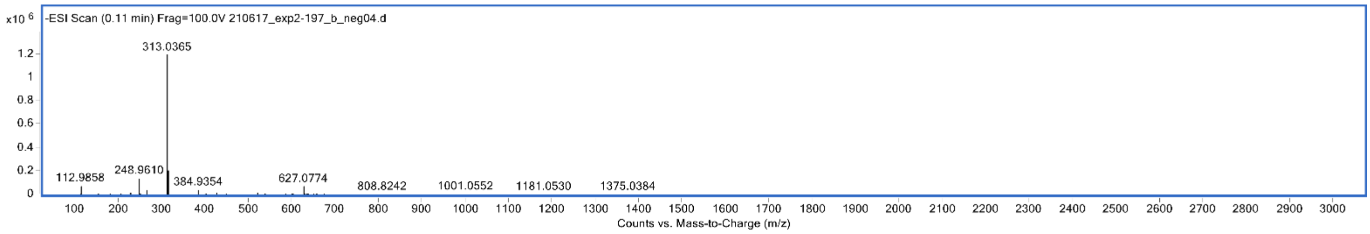

HPLC chromatogram of compound **20s** eluted using ACN/Water

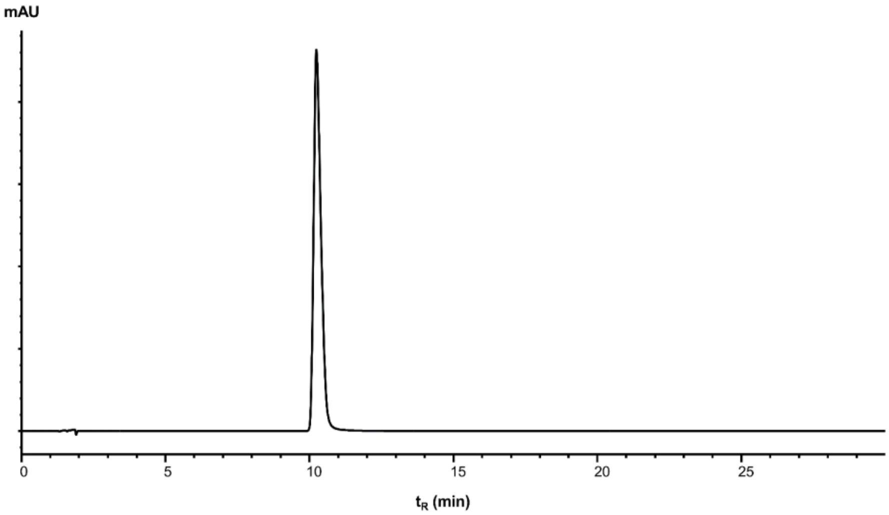

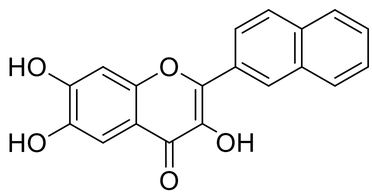

23a

^{1}H NMR spectra of compound **23a** measured in DMSO at 600 MHz

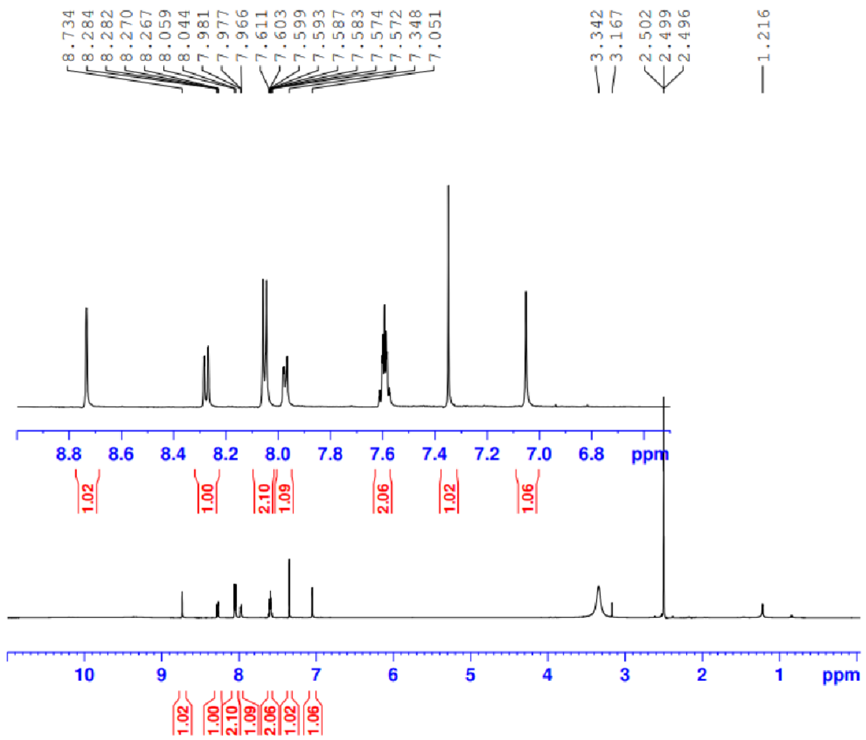

^{13}C NMR spectra of compound **23a** measured in DMSO at 600 MHz

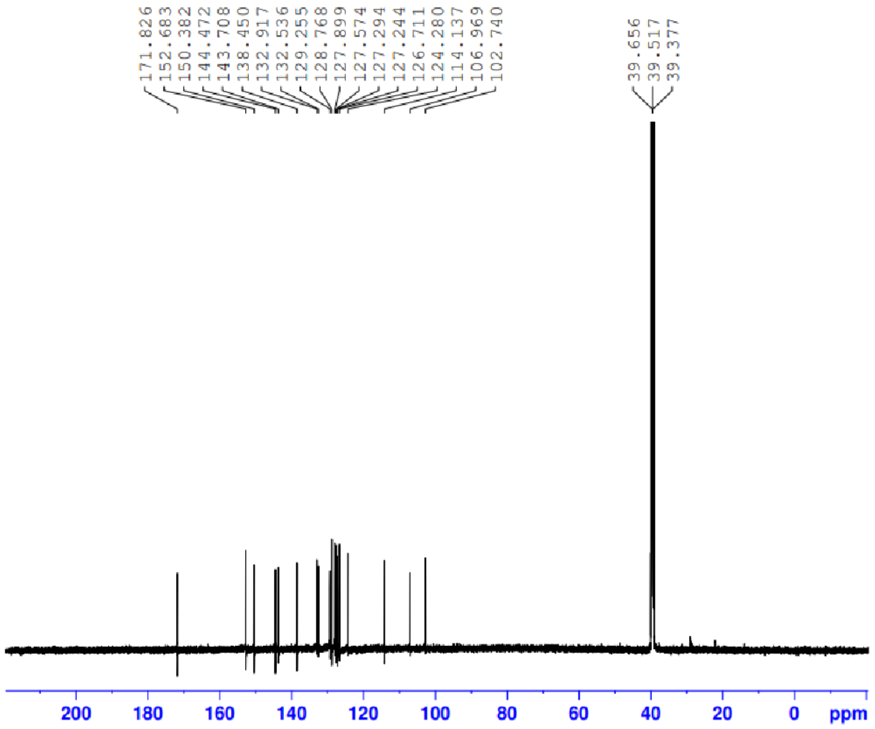

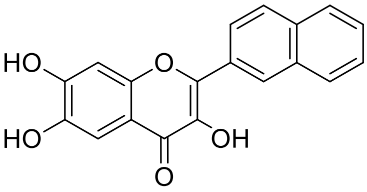

23a

HRMS chromatogram of compound **23a**

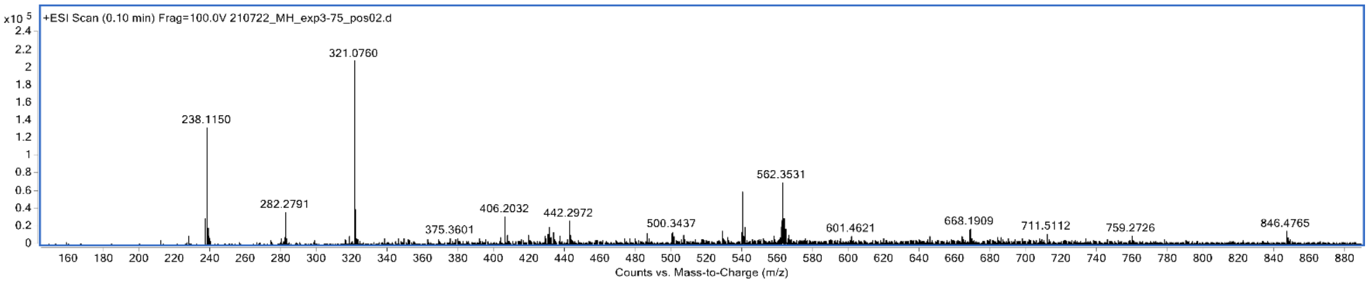

HPLC chromatogram of compound **23a** eluted using ACN/Water

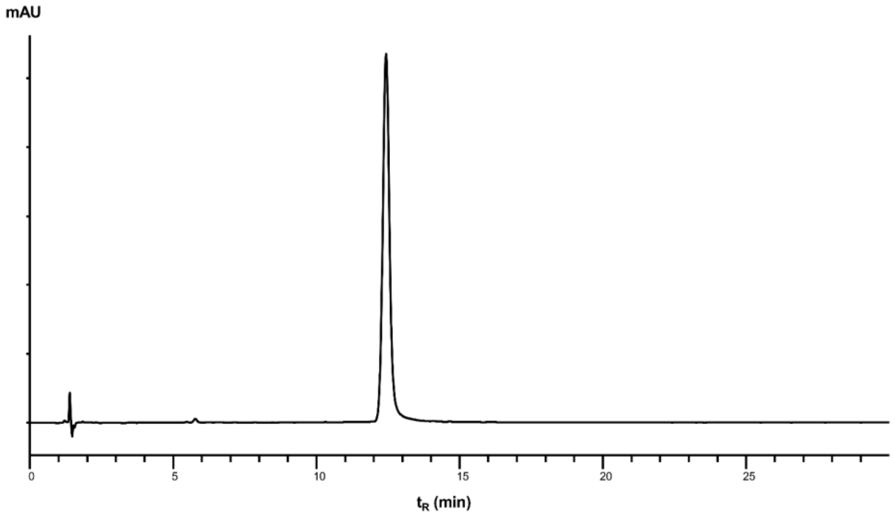

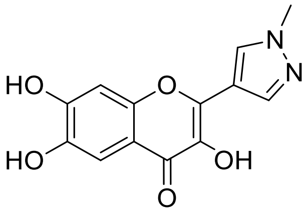

23b

^{1}H NMR spectra of compound **23b** measured in DMSO at 600 MHz

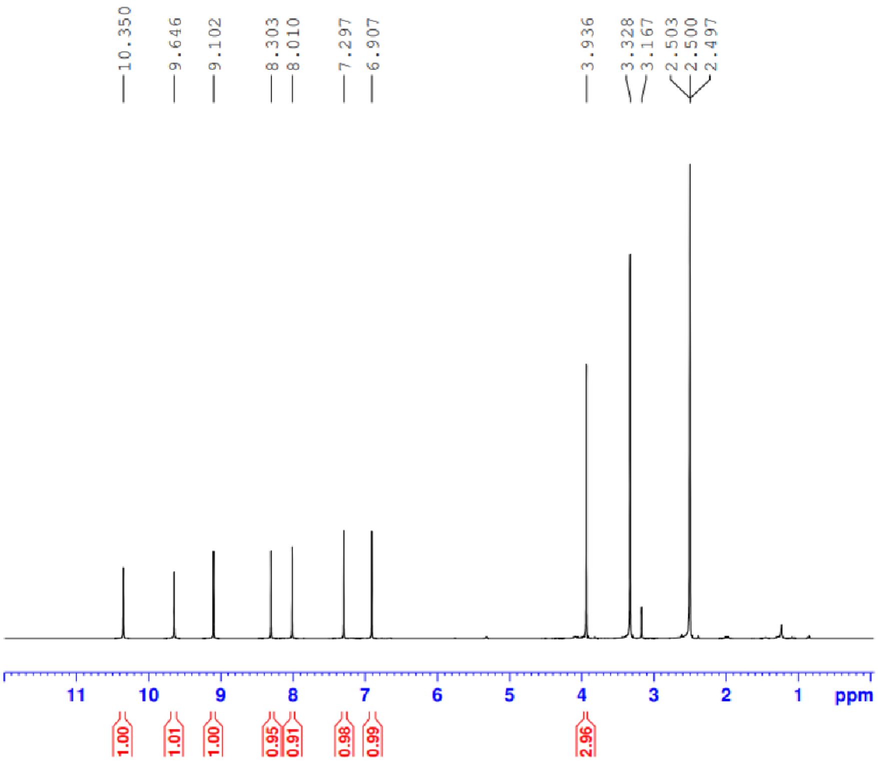

^{13}C NMR spectra of compound **23b** measured in DMSO at 600 MHz

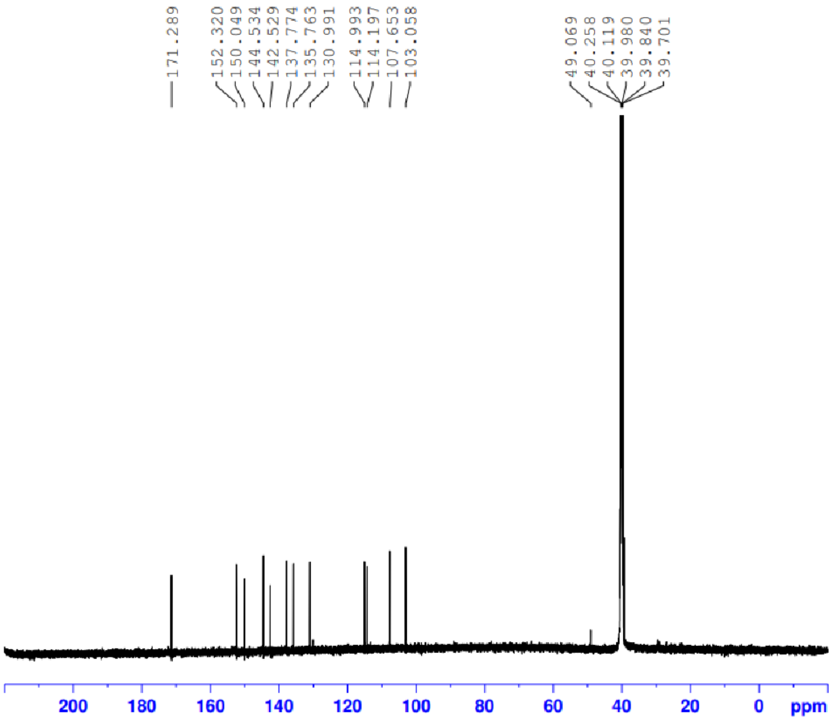

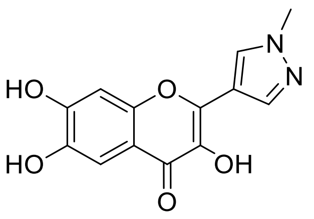

23b

HRMS chromatogram of compound **23b**

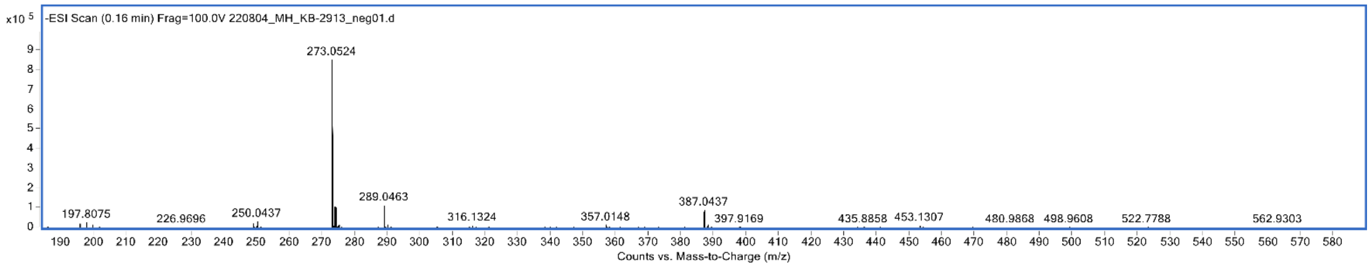

HPLC chromatogram of compound **23b** eluted using ACN/Water

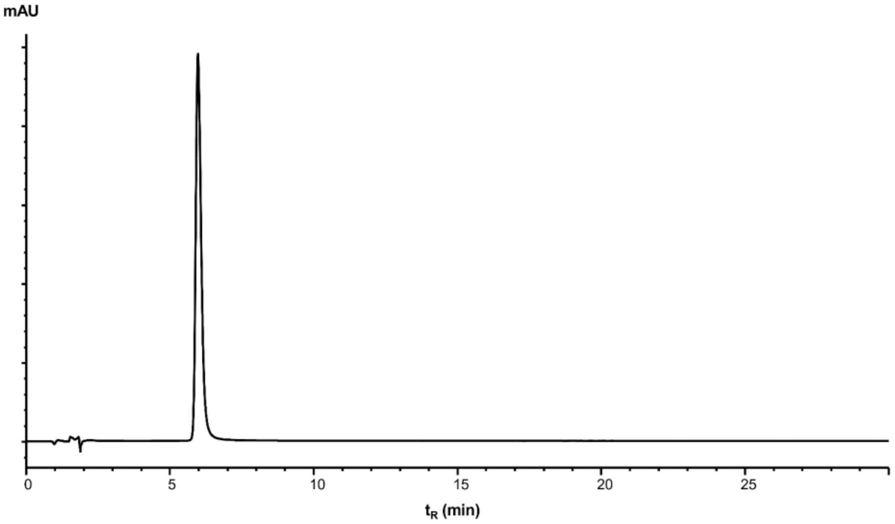

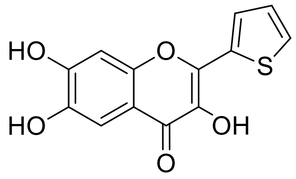

23c

^{1}H NMR spectra of compound **23c** measured in DMSO at 600 MHz

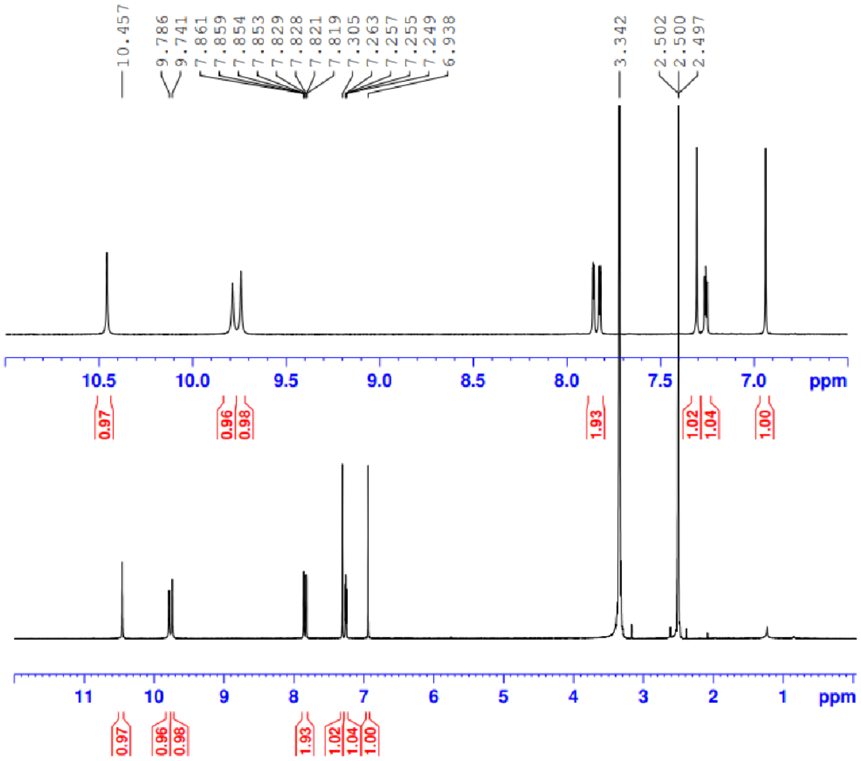

^{13}C NMR spectra of compound **23c** measured in DMSO at 600 MHz

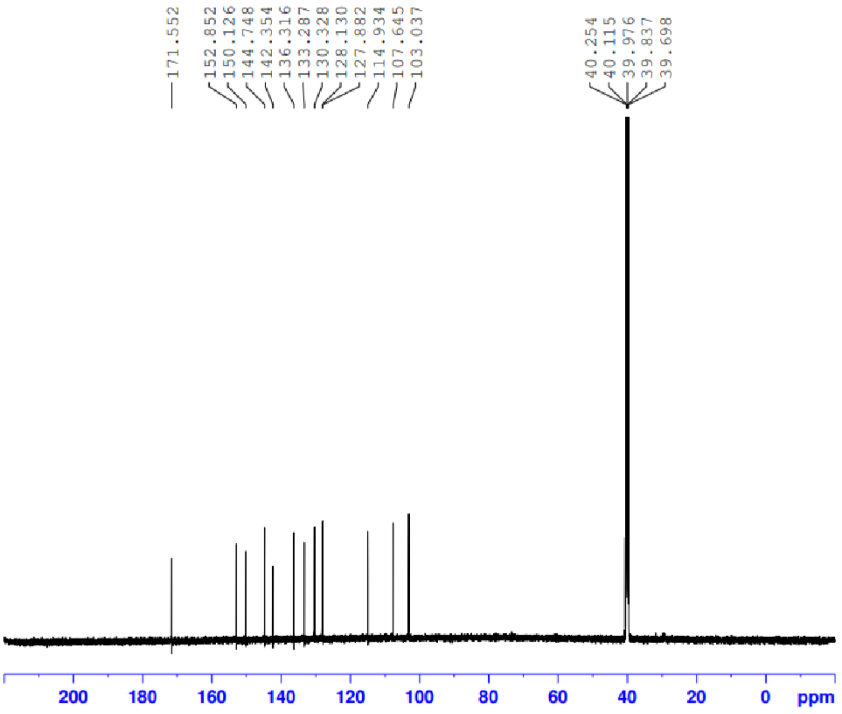

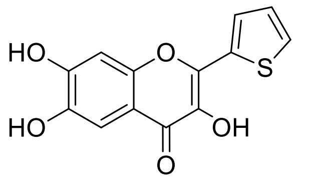

23c

HRMS chromatogram of compound **23c**

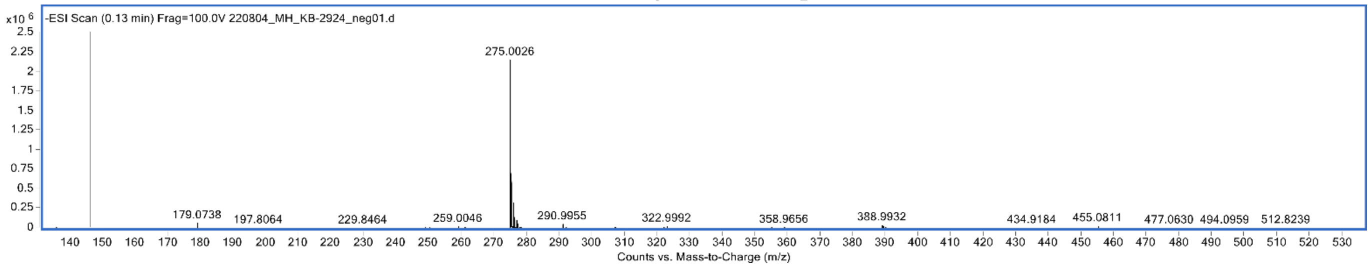

HPLC chromatogram of compound **23c** eluted using ACN/Water

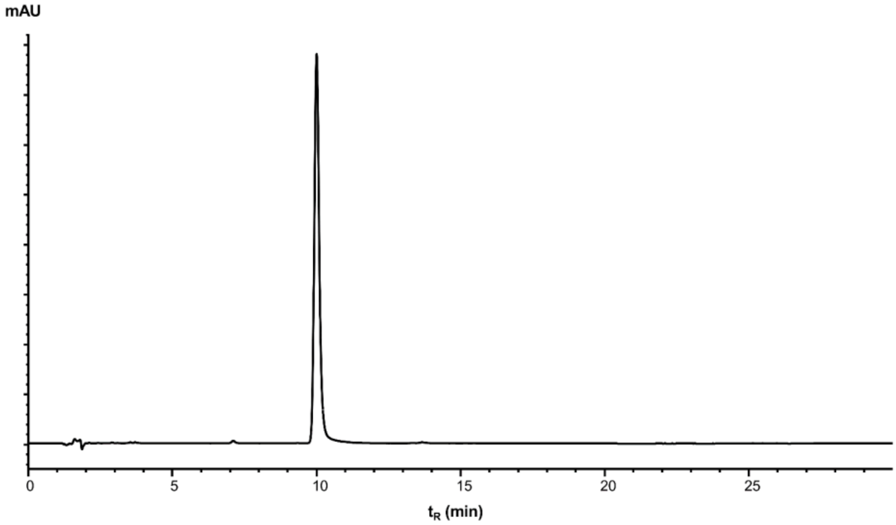

Supporting information Figure 1. IP6K2 inhibition of the synthesized compounds **9a-10i** at 10 and 50 μM concentrations

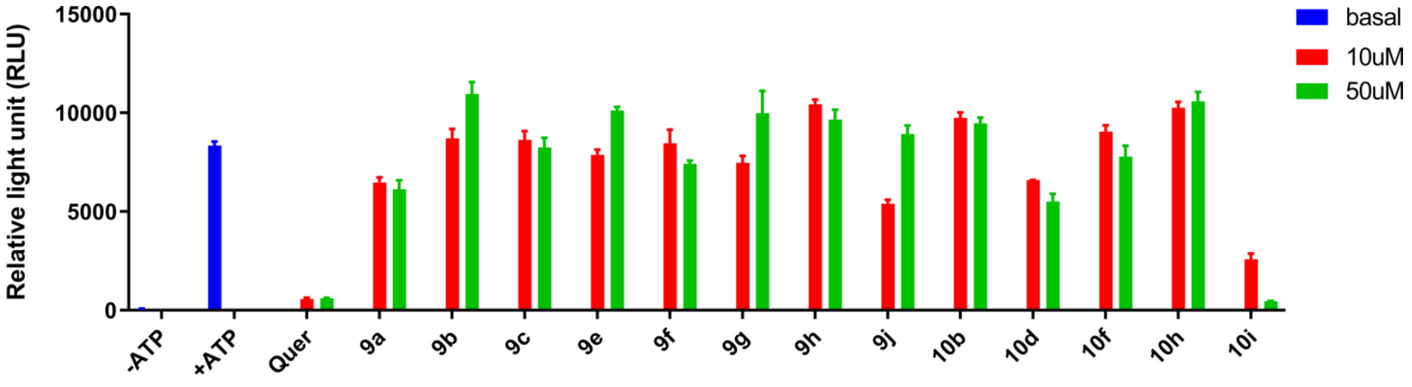

Supporting information Figure 2. Chemical structures of compounds **9a-10i**

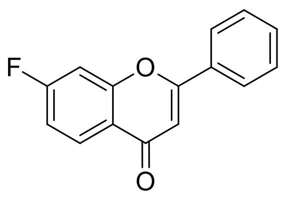

9a

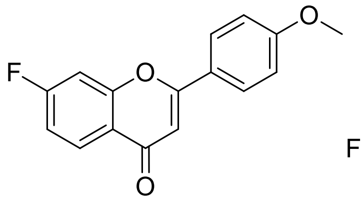

9b

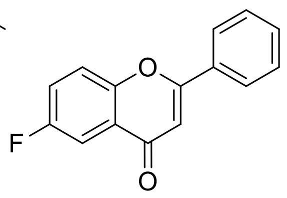

9c

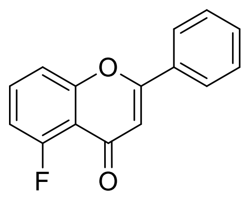

9e

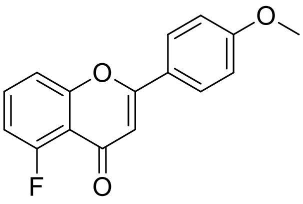

9f

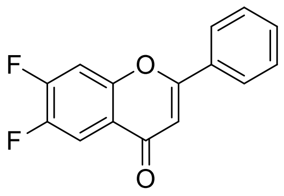

9g

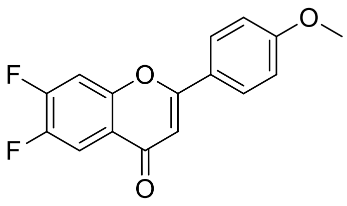

9h

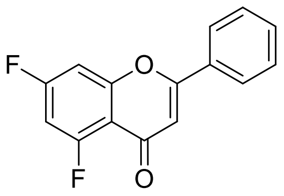

9j

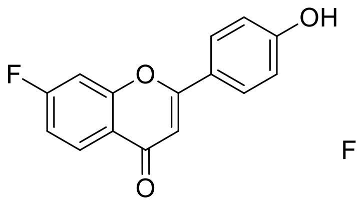

10b

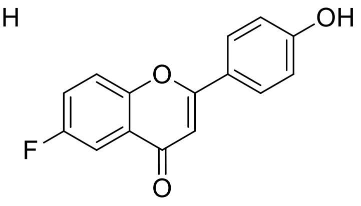

10d

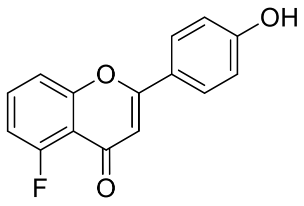

10f

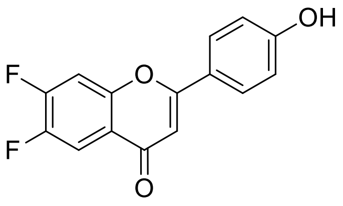

10h

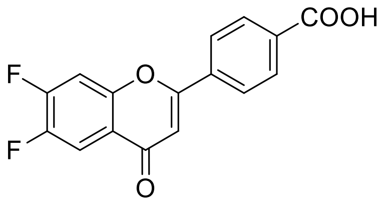

10i

Supporting information Figure 3. (a) Inhibition rate of compound **20s** and quercetin against IP6K1 (b) Inhibition rate of compound **20s** and quercetin against IP6K3

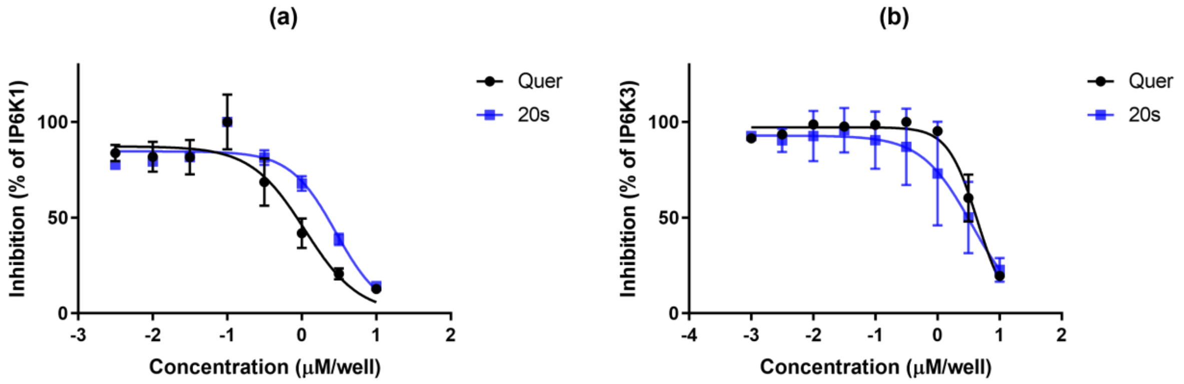

Supporting information Figure 4. Inhibition rate of compound **20s** against IP6K1, IP6K2 and IP6K3 with the addition of ATP and IP6 at final concentration of 10 μM.

IP6K3

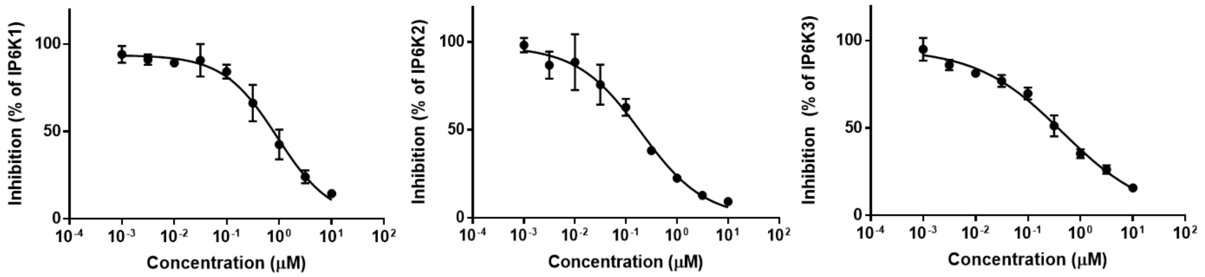

	20s
IP6K1 IC_{50} (μM)	0.9171 ± 0.21
IP6K2 IC_{50} (μM)	0.1965 ± 0.05
IP6K3 IC_{50} (μM)	0.457 ± 0.03

Supporting Information Figure 5. The cellular effects of **20s** on IP7 synthesis in the HCT116 cell line. HPLC analysis of cellular IPs (IP6, IP7) (a) and IP7 (b) from radiolabeled HCT116 cells. All experiments are prepared from HCT116 treated with DMSO, quercetin, and **20s** (10 μM) for 6 h. All values are presented as mean ± SEM. Student's *t* test was used for statistical analysis. **$P < 0.01$, N.S. non-significant.

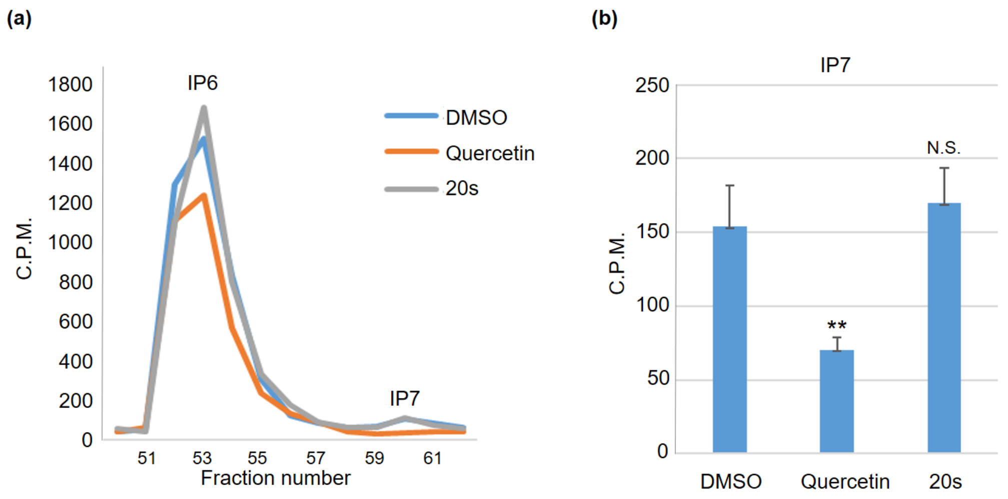

Supporting Information Figure 6. Purification of recombinant human IP6K proteins. FLAG-tagged, full-length, human IP6K1, IP6K2, or IP6K3 proteins were expressed in Sf9 insect cells and affinity purified using M2 agarose.

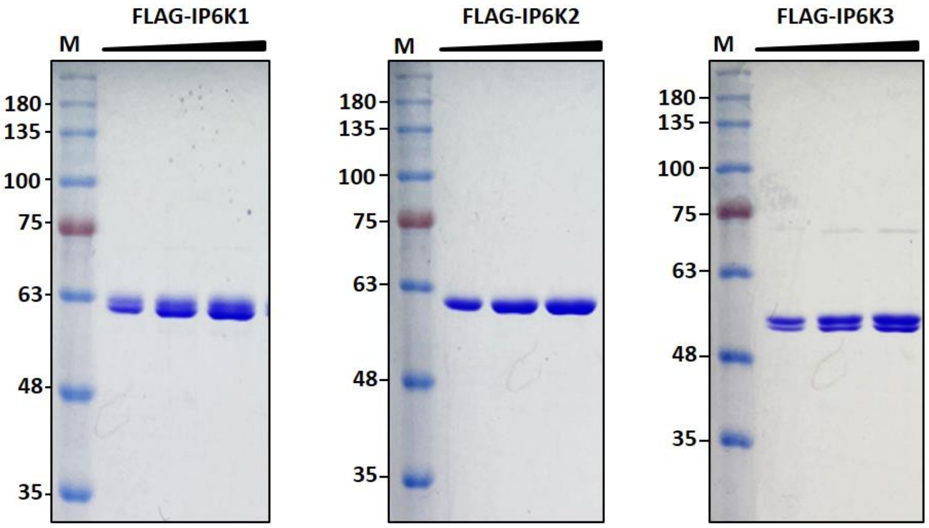

Supporting Information Figure 7. Aggregation test of **20s** and quercetin with IP6K2. Flag-tagged human IP6K2 was incubated with the compounds (**20s**, quercetin, and LI2232) at 10 and 100 μM concentrations.

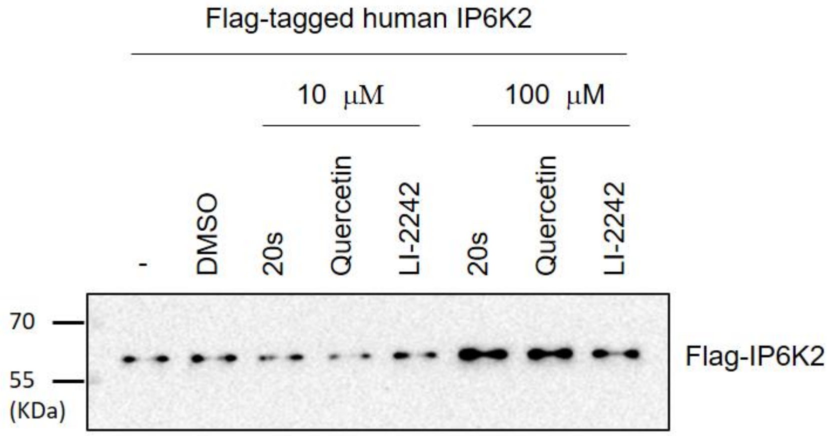

Supporting Information Table 1. PAMPA permeability of compound **20s** and quercetin

The membrane permeability of compound **20s** and quercetin along known control compounds (ranitidine, nadolol, hydrochlorothiazide, metoprolol, propranolol, chloramphenicol, verapamil, and ketoprofen) was determined in parallel artificial membrane permeability assay (PAMPA) as described previously (reference). The apparent permeability coefficient (P_{app}) of both compounds was <10 nm/sec similar to that of low permeability of control compounds (ranitidine, nadolol, hydrochlorothiazide).

**Compound**	**P_{app} (nm/s)**	**Permeability class**
Ranitidine	n.d. (<1.8)	Low
Nadolol	0.89±0.07	Low
Hydrochlorothiazide	n.d. (<1.8)	Low
Metoprolol	34.46±1.58	High
Propranolol	32.42±2.35	High
Chloramphenicol	46.72±0.63	High
Verapamil	24.79±0.46	High
Ketoprofen	31.28±2.63	High
**20s**	n.d. (<1.8)	Low
**quercetin**	5.40±0.07	Low

P_{app}: apparent permeability coefficient. n.d.: not detected in the receiver compartment (The P_{app} value was estimated to be <1.8 nm/s using the peak area ratio of 0.1 μM solution). Data are mean ± S.D. (n=3).

Reference: Oh, M.H., Lee, H.J., Jo, S.H., et al. Development of Cassette PAMPA for Permeability Screening. Biol Pharm Bull. 2017;40(4):419-424. doi: 10.1248/bpb.b16-00755.
